# Direct Light Storage with Controlled Dark Release in a Bipyridine-Based Covalent Triazine Framework

**DOI:** 10.1021/jacs.6c09757

**Published:** 2026-09-26

**Authors:** Xue Wang, Bibhuti Bhusan Rath, Nicolai Schröter, Kristina Gjorgjevikj, Rohit Mann, Shun Tokuda, Yang Wang, Viola Duppel, Lorenzo Tesi, Simon Krause, Bettina V. Lotsch

**Affiliations:** † Nanochemistry Department, 28326 Max Planck Institute for Solid State Research, Stuttgart 70569, Germany; ‡ Institute of Physical Chemistry, 9149 University of Stuttgart, Pfaffenwaldring 55, Stuttgart 70569, Germany; § Center for Integrated Quantum Science and Technology (IQST), University of Stuttgart, Pfaffenwaldring 57, Stuttgart 70569, Germany; ∥ Institut für Anorganische Chemie II, Universität Ulm, Albert-Einstein-Allee 11, Ulm 89081, Germany; ⊥ Department of Chemistry, Ludwig-Maximilians-Universität (LMU), Munich 81377, Germany; # E-Conversion and Center for Nanoscience, Garching 85748, Germany

## Abstract

Utilizing bifunctional materials capable of direct light-to-charge storage offers an attractive strategy to overcome solar intermittency and enable reliable power supply under dark conditions. Reported bifunctional materials are predominantly based on metal oxides and metal–organic compounds, whereas fully organic systems remain rare despite their rich structural diversity and tunability, which enable systematic structure–property–function investigations. Here, we reveal the light-induced, reversible electron storage properties of a two-dimensional (2D) 2,2′-bipyridine (bpy)-based crystalline covalent triazine framework (CTF), CTF-Bpy. Mechanistic studies suggest that the photoreduction of CTF-Bpy primarily proceeds through a proton-coupled, one-electron reduction of the bpy units, yielding bpyH^•^ and bpyH_2_^•+^ as the identified reduction products. Photoanodes based on CTF-Bpy show a stable photoinduced open-circuit potential (photo-OCP) of −0.92 V vs Ag/AgCl, which is so far the most negative potential reported for an organic bifunctional material. Under oxygen-free conditions, photogenerated electrons remain stable and can be controllably released on-demand in the dark, either by electrical discharge (for aqueous solar battery applications) or by reducing O_2_ to H_2_O_2_. Moreover, this work establishes CTF-Bpy as the first photochargeable covalent organic framework (COF) with stored electrons sufficiently reducing to drive H_2_ evolution in the dark in the presence of a Pt cocatalyst, highlighting the promise of using COFs for "dark photocatalysis". The systematic investigation of the light-induced electron storage in CTF-Bpy with a set of spectroscopic and (spectro-)­electrochemical techniques provides a framework for studying optoionic materials and guiding their rational design for tailored dark photocatalysis.

## Graphical abstract

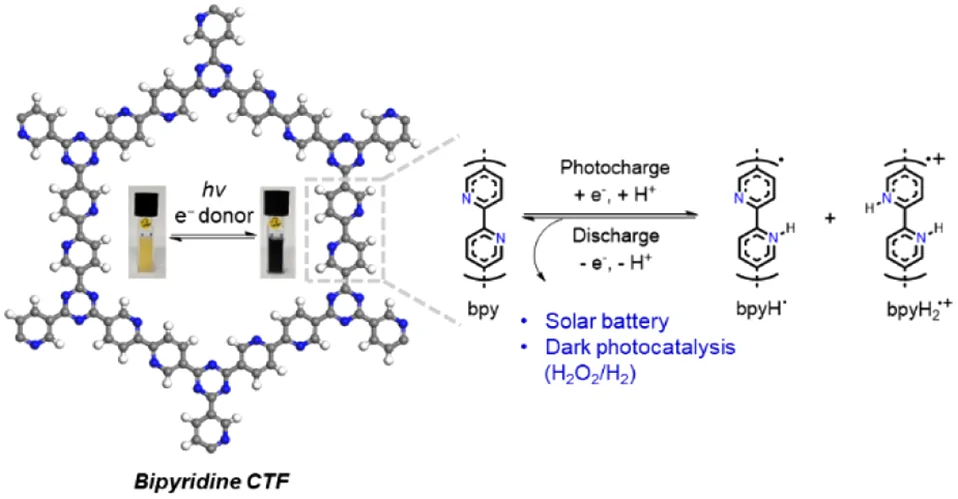

## 1 Introduction

To mitigate our over-reliance on fossil fuels, and facilitate sustainable energy solutions, converting solar energy into electricity and chemical fuels via photovoltaics and artificial photosynthesis are promising strategies. Due to the intermittent nature of solar irradiation, solar-derived electricity or fuels typically require additional storage systems to ensure a reliable 24 h energy supply.^1^ For example, photovoltaics is usually integrated with batteries as two independent operating parts by electric wires, termed as photovoltaics-battery (PV-battery) systems.^2^ Such configurations are often bulky, inflexible, and prone to energy losses through external wiring. In artificial photosynthesis, the produced chemical fuels, such as H_2_, need to be stored separately; however, limitations in current hydrogen-storage technologies remain a major barrier to adopting a large-scale hydrogen economy.^3^ Alternatively, recent advances demonstrate that direct solar-to-electrochemical storage (i.e., in a solar battery)^4–6^ and on-demand H_2_ production in the dark^7,8^ are possible by using bifunctional materials capable of simultaneous light harvesting and charge storage. Utilization of such materials can simplify wired PV-battery devices to minimize energy losses, and alleviates challenges associated with H_2_ storage.

As compared to conventional photovoltaic materials and photocatalysts, the key attribute of such bifunctional materials is their ability to stably trap charges after illumination ceases for minutes or even hours. Depending on the type of charge stored, charge-storage materials can be classified as hole-storage^9^ or electron-storage materials,^10^ and here, we focus on electron-storage materials. In a typical colloidal electron storage system, the presence of a sacrificial electron donor (SED) is generally required to scavenge photogenerated holes and enable the accumulation of photogenerated electrons within the system.^11^ To date, reported materials capable of electron storage under irradiation are largely restricted to inorganic metal oxides and metal–organic compounds. In metal oxides such as TiO_2_,^12,13^ SnO_2_,^14^ MoO_3_,^15^ WO_3_,^16,17^ V_2_O_5_ ^18^ and their derivatives,^19,20^ electron storage relies on the intrinsic transition of metal redox activity.^21^ In addition, for metal–organic complexes^22–24^ and metal–organic frameworks (MOFs),^25–29^ electron storage can further originate from a cooperative effect between the metal and organic ligand. More recently, zinc indium sulfide nanocrystals^30^ and a hybrid system based on photoactive Ru­(II) tris­(bipyridine) complexes^31^ have also been reported to store electrons under light. Poly­(heptazine imide) (PHI)-type 2D ionic carbon nitrides represent a prototypical carbon-based system for light-induced electron storage, where electron stabilization is achieved via cation intercalation (e.g., K^+^ in K-PHI).^4,7^ So far, these PHI-type 2D carbon nitrides have been used as photoanode in aqueous solar battery applications^4^ and as photocatalyst for time-delayed H_2_ and H_2_O_2_ production in the dark.^7,32,33^ While PHI-type 2D carbon nitrides represent a benchmark material for light-induced electron storage and dark photocatalysis, particularly in terms of stability, scalability and catalytic performance, their inertness and the resulting limited chemical functionality severely restricts their structural diversity to achieve tunable optoelectronic and charge storage properties.

Compared to PHI-type 2D ionic carbon nitrides, covalent organic frameworks (COFs) offer molecular-level tunability with rich structural diversity,^34^ making them promising candidates for next-generation electron storage materials. For example, COFs embedded with redox-active naphthalenediimide (NDI)^35,36^ and perylenetetracarboxylic diimide (PTCDI)^37^ have enabled direct solar-to-electrochemical energy storage, and a dihydroxybenzene containing COF integrated with BiVO_4_ has been employed to decouple artificial photosynthesis through photoelectrochemical energy storage.^38^ However, to date, no COF has been reported mirroring PHI-type 2D carbon nitride functioning both as photoanode for solar batteries and as a catalyst for dark photocatalysis, including H_2_ production. A key challenge in driving H_2_ evolution in the dark is that the photoinduced open-circuit potential (photo-OCP) of the electron-storage material needs to be more negative than the potential of the H^+^/H_2_ redox couple (−0.41 V vs NHE, at pH 7 and 25 °C).^39^ For instance, among the reported electron storage materials, (TBA)^+^(NbWO_6_)^−^,^20^ Ti-MOF^29^ and K-PHI^4,7^ with photo-OCP of ca. −0.50 V, −0.80 V and −0.60 V (all vs NHE) can drive dark H_2_ evolution, whereas NDI-COF (−0.45 V vs NHE) cannot,^36^ likely because the small redox potential difference (ΔE ≈ 0.04 V) is insufficient to drive the H^+^/H_2_ reaction. Aside from this, a more negative photo-OCP is also desired for photoanodes to deliver sufficient charging voltages for practical solar battery applications.^6^ Note that, in this work, we tentatively use the photo-OCP value as an approximate measure of the reducing power of the photochargeable electron-storage materials, while acknowledging that the measured OCP can be influenced by multiple factors beyond the intrinsic redox potential of the material, such as the FTO substrate, solvent properties, and recombination processes.^40^

In this work, we selected 2D covalent triazine frameworks (CTFs)—a subclass of COFs characterized by the triazine linkage and high chemical/thermal stability^41^—to study their light-induced electron storage properties. The nitrogen-rich, conjugated backbone of CTFs enhances light absorption,^42^ thus making 2D CTFs attractive candidates for time-delayed photocatalysis in the dark. To impart the required redox-activity to the framework, we incorporate 2,2′-bipyridine as a linker. Although 2,2′-bipyridine is a well-established two-electron redox unit,^43^ prior studies have largely focused on its incorporation into metal–organic systems.^24,26,27^ The fact that molecular 2,2′-bipyridine itself can undergo photoreduction in the presence of SEDs has remained largely overlooked in this field.^44,45^ Moreover, literature reports indicate that the one-electron reduced 2,2′-bipyridine is more reducing than the two-electron reduced NDI,^46^ highlighting the potential of 2,2′-bipyridine-embedded CTFs to extend solar battery charging voltages and possibly drive dark photocatalysis (i.e., H_2_ evolution).

Based on the above considerations, in this work, we investigate a known 2,2′-bipyridine-based 2D CTF—CTF-Bpy—to assess its ability for light-induced electron storage and controlled dark release. We show that, under illumination, CTF-Bpy acts as an electron reservoir in water in the presence of 4-methylbenzyl alcohol (4-MBA) as SED. The photoinduced electron accumulation is reversible, with air/O_2_ exposure quenching the stored electrons. Using a suite of spectroscopic and spectro-electrochemical methods, we elucidate that electron storage in CTF-Bpy arises from one-electron reduction of the 2,2′-bipyridine (bpy) units via a proton-coupled process, with bpyH^•^ and bpyH_2_^•+^ identified as the reduced species. Photoelectrochemical measurements of the CTF-Bpy photoanode show a photo-OCP of −0.92 V vs Ag/AgCl (−0.72 V vs NHE), which is more negative than the reported value of K-PHI (−0.60 V vs NHE). The stored electrons remain stable within the framework, allowing their controlled release in the dark as electrical energy for aqueous solar battery applications, or alternatively, to drive oxygen reduction to form H_2_O_2_ and proton reduction for the H_2_ evolution reaction (the latter with a Pt cocatalyst) in the dark. This represents the first example of bifunctional COFs where the stored electrons are reductive enough to drive H_2_ evolution in the dark. The systematic study of light-induced electron storage in CTF-Bpy lays out a general protocol for the identification and rational design of organic optoionic materials for direct solar energy conversion and storage.

## 2 Results and Discussion

### 2.1 Synthesis and Characterization

CTF-Bpy was synthesized following a literature procedure by reacting 2,2′-bipyridine-5,5′-dicarboximidamide dihydrochloride with 2,2′-bipyridine-5,5′-dicarbaldehyde under solvothermal conditions to afford CTF-Bpy as fluffy yellow powder (Figure 1a, see Supporting Information for details).^47,48^ PXRD analysis of CTF-Bpy reveals a pattern consistent with previous reports, suggesting a 2D **hcb** topology with AA stacking mode.^48^ The reflections at 4.7°, 8.4°, 9.8° and 26.4° can be assigned to the {100}, {21̅0}, {200} and {001} planes, respectively. Pawley refinement confirms a hexagonal lattice with space group *P*6/*m* and unit cell parameters *a* = *b* = 21.944(9) Å and *c* = 3.537(8) Å (Figure 1b). The Fourier transform infrared (FTIR) spectrum of CTF-Bpy exhibits the characteristic vibrations of the triazine unit at 1504 and 1335 cm^–1^, confirming the formation of triazine linkages. The disappearance of the amino and aldehyde absorption indicates near-complete reaction of the two precursors (Figure S3). The solid-state ^1^H magic-angle spinning (MAS) NMR spectrum of CTF-Bpy shows a sharp signal at 8.2 ppm, corresponding to the hydrogen atoms on the bipyridine units (Figure S4). In the ^13^C cross-polarization magic-angle spinning (CP MAS) NMR spectrum, the signal at 169.7 ppm represents the carbon atoms from the triazine ring. The signals at 157.5, 149.9, 135.3, 130.3, and 120.8 ppm can be assigned to the carbon atoms from the bipyridine units (Figure S5). N_2_ sorption measurement of CTF-Bpy at 77 K yields a total pore volume of 0.39 cm^3^ g^–1^ at *P*/*P*_0_ = 0.97 and a Brunauer–Emmett–Teller (BET) surface area of 794 m^2^ g^–1^. Fitting the quenched solid density functional theory (QSDFT) model to the N_2_ adsorption isotherm derives a pore size distribution centered at 1.50 nm, in good agreement with the reported value (Figure 1c, Figure S6).^48^ Scanning electron microscopy (SEM) images of CTF-Bpy reveal a morphology of aggregated particles (Figure S7). Transmission electron microscopy (TEM) images of CTF-Bpy exhibit crystalline domains, and the corresponding selected area electron diffraction (SAED) pattern displays a lattice spacing of 1.80 nm, indexed to the {100} plane of the CTF-Bpy structural model (Figure S8). Thermogravimetric analysis (TGA) reveals high thermal stability of CTF-Bpy, with no significant mass loss observed up to 550 °C under Ar (Figure S9).

**Figure 1:**
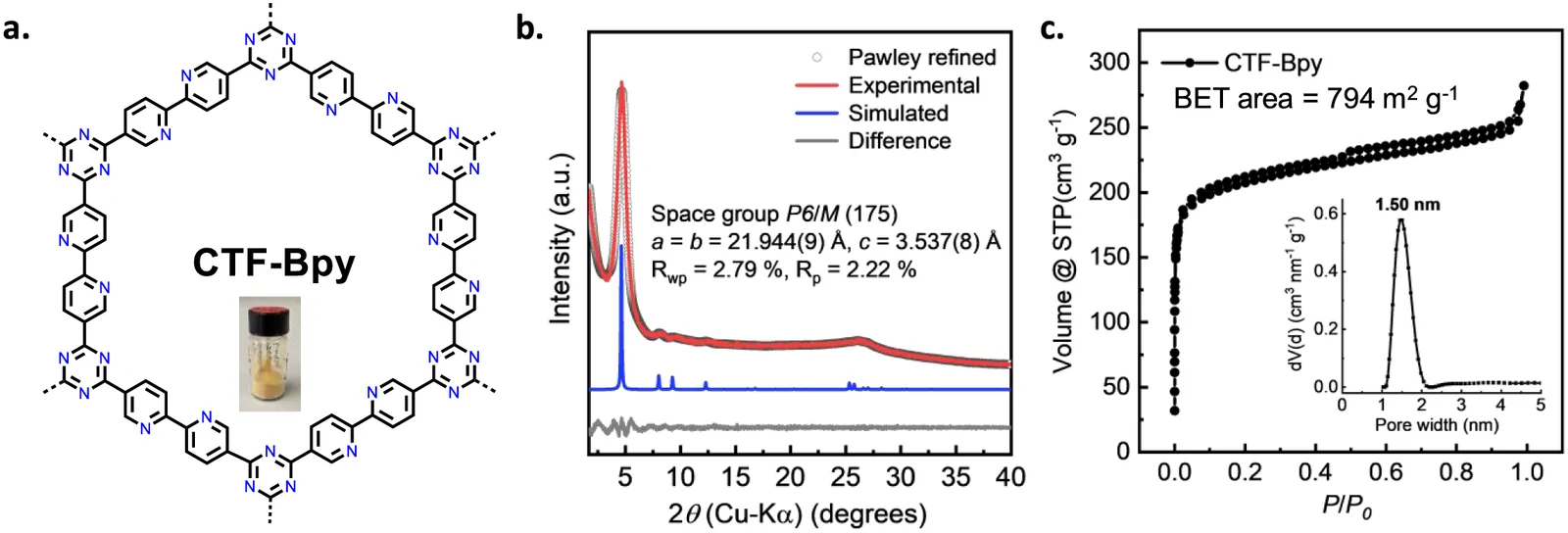
Structural characterizations of CTF-Bpy. (a) Chemical structure of CTF-Bpy. Inset is a photograph of CTF-Bpy powder sample. (b) PXRD patterns of CTF-Bpy are shown with the experimental pattern in red, Pawley refined data as black circles, calculated pattern from the simulated structural model in blue and the difference (observed minus refined data) in gray. (c) N_2_ sorption isotherm and the derived pore size distribution of CTF-Bpy.

### 2.2 Reversible Light-Induced Radical Formation

According to the literature, 2,2′-bipyridine (bpy) undergoes two consecutive one-electron reductions, yielding the radical monoanion (bpy^•–^) and the dianion (bpy^2–^), respectively.^49,50^ Because the two reduced states of molecular bpy exhibit a distinct fingerprint absorption spectrum in the visible and near-infrared (NIR) region,^43,51,52^ we first probed the photoreduction properties of CTF-Bpy using ultraviolet–visible-NIR (UV–vis–NIR) spectroscopy.

The photoreduction of CTF-Bpy was explored in an O_2_-free aqueous suspension in the presence of 20 mM 4-MBA as SED. The UV–vis–NIR spectrum of the CTF-Bpy suspension exhibits an intense absorption band between 280–400 nm, corresponding to the characteristic π–π* transitions of the triazine and pyridine rings.^52–54^ The optical bandgap of CTF-Bpy is calculated to be 2.3 eV (Figure S10). Upon 365 nm UV illumination (nominal power *P*_*nominal*_ ≈ 237 mW cm^–2^), the suspension shows a rapid color change from yellow to black in 1 min, accompanied by the evolution of new, broad absorption bands in the UV–vis–NIR spectrum between 430–1370 nm. This new absorption closely resembles that of bpy^•–^, as both exhibit absorption in the visible and NIR region^51,52,54^ and can be attributed to the singly occupied molecular orbital (SOMO)-involved low-energy transitions of bpy^•–^, which are responsible for the observed yellow-to-black color change of the suspension (Figure 2a).^52,55^ The new band is, however, more intense and red-shifted relative to molecular bpy^•–^, likely related to the extended π-conjugated framework of CTF-Bpy. The possible coexistence of bpy^2–^ cannot be excluded on the basis of the UV–vis–NIR spectrum alone owing to potential spectral overlap, as its absorption is expected to appear at shorter wavelength as compared to bpy^•–^.^43,51^ Under continuous illumination, the new absorption band reaches saturation after 10 min (Figure 2b). In addition, the signal at 290 nm increases slightly, which is attributed to *p*-tolualdehyde, the oxidation product of 4-MBA, as confirmed by control experiments (Figure S11). Upon air/O_2_ exposure, the photoreduced CTF-Bpy is reoxidized, evident from the disappearance of the absorption bands between 430–1370 nm, ultimately yielding the original spectrum and the yellow color (Figure S12). The reoxidation by air/O_2_ is accompanied by H_2_O_2_ formation (Figure S13), which will be discussed further below in more detail. The photoreduction and subsequent oxidation by air/O_2_ can be repeated over multiple cycles, indicating the reversibility of photoreduction and reusability of CTF-Bpy for photocharging and subsequent charge release (Figure 2b, inset).

**Figure 2:**
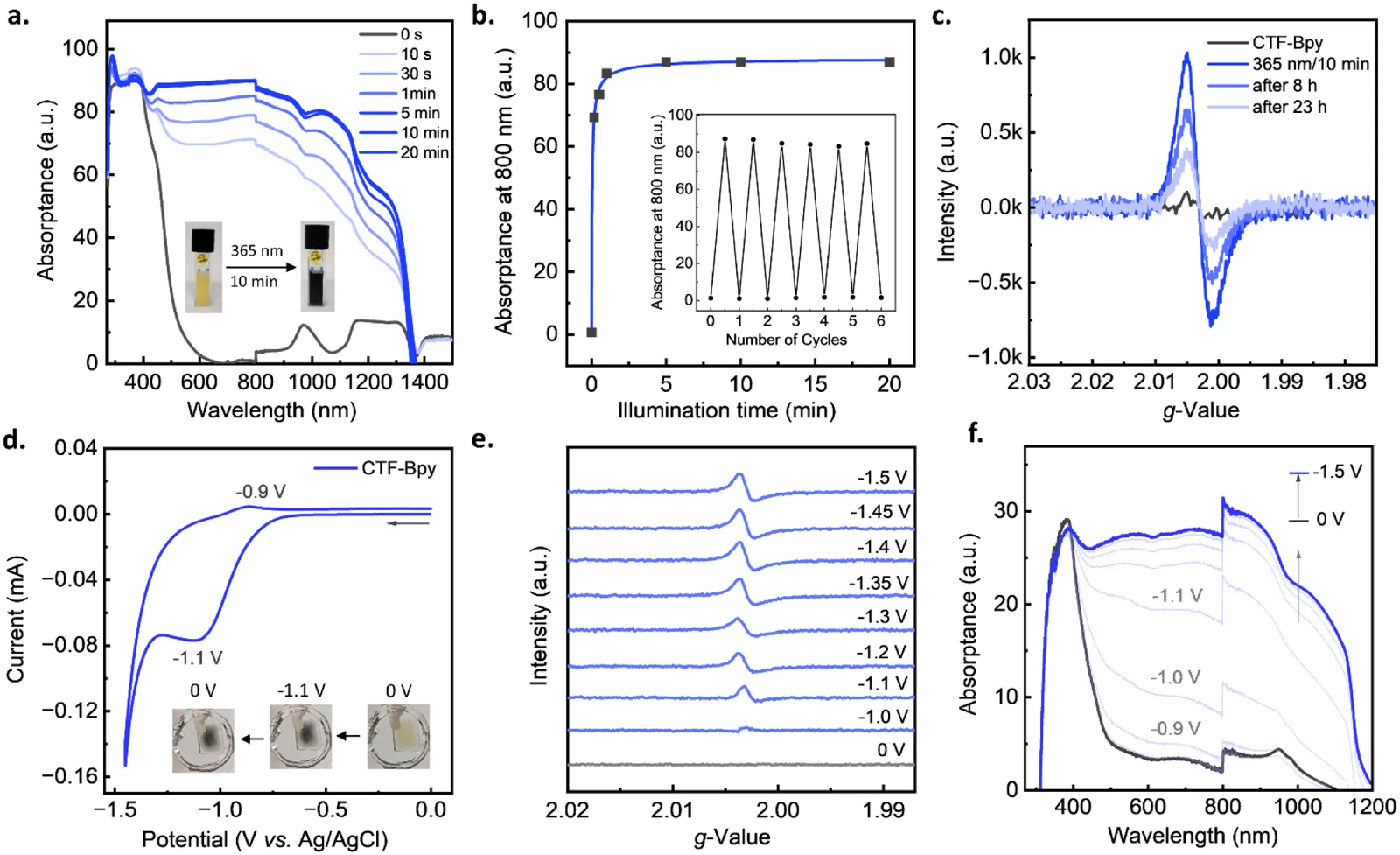
Determination of light-induced radical formation in CTF-Bpy. (a) UV–vis–NIR absorptance spectra of an aqueous CTF-Bpy suspension (1 mg/3 mL) in 20 mM 4-MBA under continuous 365 nm illumination. The inset shows photographs of the suspension before and after 10 min illumination. (b) Dependence of absorptance at 800 nm on illumination time. The inset shows the reversibility and recyclability after air exposure. (c) EPR spectra showing the light-induced radical formation of the suspension and the radical stability over time, after light is switched off. Microwave frequency = 9.6146 GHz. (d) CV curve of CTF-Bpy on an FTO-coated glass electrode at a scan rate of 10 mV s^–1^ in 0.1 M KCl aqueous electrolyte and associated color change of the film at marked potentials during the CV scan. (e) *In situ* EPR (microwave frequency = 9.4226 GHz) and (f) UV–vis–NIR spectro-electrochemical analysis of CTF-Bpy during a cathodic scan from 0 to −1.5 V. *The step at 800 nm in (f) is due to the default detector change of the spectrophotometer.

In addition to 4-MBA, 20 mM triethanolamine (TEOA) and methanol (MeOH) were also tested as SED for CTF-Bpy photoreduction in H_2_O. On comparing the color change and the intensity of the new absorption band after 5 min illumination, the efficiency of the SEDs follows the order of 4-MBA > TEOA > MeOH (Figure S14). Water can also work as a SED, but proved to be the least effective (Figure S15). Moreover, by conducting UV–vis–NIR studies with various 4-MBA concentrations under illumination, the optimum concentration of 4-MBA as the SED for CTF-Bpy is determined to be 20 mM in water (Figure S16), which was then used for subsequent experiments.

To verify the role of redox-active bpy units for the photoreduction property of CTF-Bpy, the photochromic behavior of the biphenyl analogue of CTF-Bpy, CTF-Bph, was investigated (Figure S17, S18–S21).^56^ Upon 5 min of irradiation, the absorption edge of the UV–vis–NIR spectrum of CTF-Bph red-shifted from approximately 430 to 480 nm. Extending the irradiation time to 30 min resulted in no additional absorption features compared to the spectrum observed after 5 min. Notably, the light-induced UV–vis–NIR spectral changes of CTF-Bph were irreversible and did not revert upon exposure to air, in marked contrast to the reversible change observed for CTF-Bpy (Figure S17). While the control study of CTF-Bph highlights the crucial role of the bpy units in the reversible photoreduction of CTF-Bpy, it is also important to note that the presence of the bpy units within a COF does necessarily result in photochromic behavior (Figure S22).

It is known that the paramagnetic one-electron reduced bpy^•–^ gives rise to an electron paramagnetic resonance (EPR) signal, whereas the diamagnetic two-electron reduced bpy^2–^ is EPR silent.^57^ To identify the reduced state of the bpy unit in CTF-Bpy upon illumination, we conducted EPR measurements with an aqueous suspension of CTF-Bpy with 4-MBA. As shown in Figure 2c, 10 min of illumination significantly increases the EPR signal compared to the dark, resulting in a clear single peak with a *g* value of 2.0030(1). This value is in agreement with the reported value for bpy^•–^,^50,57^ supporting light-induced radical formation. After illumination is stopped, the EPR signal intensity gradually decreases over time, possibly due to oxygen (air) leaking into the suspension, which quenches the radicals. Nevertheless, even after 23 h, there is still detectable amounts of radicals (ca. 35% intensity retention as compared to the signal after 10 min illumination), highlighting excellent radical stability in the dark. In summary, UV–vis–NIR and EPR results together confirm the formation of the one-electron reduced bpy^•–^ within CTF-Bpy under illumination, but do not rule out the possible coexistence of bpy^2–^, which is EPR silent.^57^

To check for the possible presence of bpy^2–^, we then conducted cyclic voltammetry (CV), *in situ* EPR- and UV–vis–NIR spectro-electrochemistry measurements. CV measurements of CTF-Bpy were performed using a three-electrode setup with CTF-Bpy on a fluorine-doped tin oxide (FTO)-coated glass substrate as working electrode, a reference electrode and a Pt counter electrode in O_2_-free electrolytes (Figure S23). The working electrode was prepared by drop-casting a CTF-Bpy suspension in *N*-methyl-2-pyrrolidone (NMP) onto the FTO-coated glass substrate, yielding a uniform film with an average thickness of ca. 2.7 μm (Figure S24). In acetonitrile containing 0.1 M tetrabutylammonium perchlorate (TBAClO_4_), two redox waves at −1.9 V and −2.4 V vs ferrocene/ferrocenium (Fc/Fc^+^) are observed, corresponding to the successive reductions of the bpy unit in CTF-BPy to bpy^•–^ and bpy^2–^ , respectively (Figure S25). In contrast, in 0.1 M KCl aqueous electrolyte, only one reduction wave appears at −1.1 V vs Ag/AgCl (saturated KCl), suggesting that only bpy^•–^ is accessible—likely due to the limited potential window of the aqueous electrolyte (Figure 2d, Figure S26). During the cathodic scan, the CTF-Bpy film shows an electrochromic color change from yellow to black at potentials more negative than −1.0 V and remains black even after the anodic sweep to 0 V (inset of Figure 2d). The retention of the black color, together with a substantially lower anodic peak current (i_pa_) relative to the cathodic peak current (i_pc_), indicates an electrochemically irreversible reduction of CTF-Bpy during the CV measurements in aqueous electrolyte. We propose the observed irreversible reduction of CTF-Bpy here is due to protonation of the reduced bpy species. This interpretation is supported by CV measurements of CTF-Bpy in dry acetonitrile containing 0.1 M TBAClO_4_ with 0, 0.1, 0.2, or 0.5 M H_2_O (Figure S27). In the first CV cycle, two distinct oxidation waves are observed following cathodic reduction in the absence of H_2_O (0 M). These oxidation features progressively weaken and nearly disappear as H_2_O concentration increases from 0.1 to 0.5 M, providing direct evidence that H_2_O promotes the irreversible reduction of CTF-Bpy. It is plausible that H_2_O acts as a proton source for protonation of the reduced bpy species, thereby stabilizing the reduced state, consistent with previous reports that protonation of radical anions or dianions can lead to irreversible electrochemical reduction.^58^ Nevertheless, this reduction is chemically reversible, as supported by the CTF-Bpy film reverting from black to its original yellow color upon air exposure after the CV measurement.

In addition to the CV results in aqueous electrolyte, which suggest the formation of the one-electron reduced bpy species (e.g., bpy^•–^) within the potential window of 0 ∼ −1.5 V, *in situ* EPR spectro-electrochemistry measurements were performed to further verify that the one-electron reduced bpy species is the sole species formed (Figure 2e, Figure S28). During the cathodic scan, no EPR signal is observed between 0 and −0.9 V (vs Ag/AgCl, 0.5 M KCl aqueous electrolyte). Upon reaching −1.0 V, a signal appears at *g* = 2.0030(1), and the intensity of this signal continuously increases without noticeable decrease upon further scanning to −1.5 V (Figure 2e, Figure S28), corroborating the formation of sole one-electron reduced bpy species during the CV experiments within the potential window of 0 ∼ −1.5 V. We further performed operando UV–vis–NIR spectro-electrochemical measurements to get a better understanding of the redox states involved in the photoreduced CTF-Bpy (Figure 2f). The pristine CTF-Bpy film shows an absorption band between 300–500 nm centered at 385 nm. As the cathodic sweep progresses, a new absorption band emerges at −0.9 V between 450–1200 nm and grows continuously as the scan proceeds to −1.5 V. This new absorption profile matches with the radical spectrum observed under illumination (Figure 2a), confirming that it is the one-electron reduced bpy radical species formed within CTF-Bpy under light.

### 2.3 Mechanism of Light-Induced Radical Formation

To probe structural and chemical changes of CTF-Bpy upon photoreduction, we conducted PXRD measurements. In molecular bpy, one-electron reduction strengthens the inter-ring bond (C_py_–C_py, py_ = pyridine) and reduces the bond length from 1.48 Å to 1.43 Å.^49^ However, this change is too subtle to be resolved by PXRD, hence the experimental and simulated patterns of the neutral and photoreduced CTF-Bpy are indistinguishable (Figure S29).

While the spectroscopic and spectro-electrochemical results in Figure 2 support the formation of one-electron reduced bpy species, previous studies have shown that photoreduction of molecular bpy in aqueous solution produces bpyH^•^ and bpyH_2_^•+^.^59^ To examine whether proton uptake occurs during the photoreduction of CTF-Bpy, the pH of the CTF-Bpy aqueous suspension (1 mg/mL) containing 20 mM 4-MBA was measured before and after 10 min of 365 nm irradiation, yielding pH values of 8.60 and 8.74, respectively. This pH increase corresponds to 0.7 × 10^–6^ mmol L^–1^ of proton loss from the aqueous medium. Because oxidation of 4-MBA to *p*-tolualdehyde releases protons, the amount of *p*-tolualdehyde produced during irradiation was also quantitatively determined by High-Performance Liquid Chromatography (HPLC) (Figure S30). After 10 min of irradiation, the *p*-tolualdehyde concentration increased by 0.28 mmol L^–1^, corresponding to 0.56 mmol L^–1^ of proton release from 4-MBA oxidation. This significant discrepancy in the supplied amount of protons and actual change in the proton concentration (+0.56 mmol L^–1^ versus −0.7 × 10^–6^ mmol L^–1^) indicates that the protons released from 4-MBA oxidation are nearly quantitatively consumed during CTF-Bpy photoreduction, likely to compensate the negative charges generated on the bpy moieties. Thus, these results suggest that the reduced bpy units in photoreduced CTF-Bpy exist in the protonated form (e.g., bpyH^•^ and bpyH_2_^•+^) rather than anion radicals (i.e., bpy^•–^), with protons being supplied by 4-MBA oxidation.

The proton-coupled nature of CTF-Bpy reduction in aqueous medium is further supported by CV measurements over a wide pH range (pH 1.63–13.54) in 0.1 M KCl aqueous electrolytes (Figure S31). While one reduction wave accompanied by a corresponding oxidation wave was observed over the entire pH range investigated, the half-wave potential (*E*_1/2_) shifted progressively toward more negative potentials with increasing pH, indicating the redox behavior of CTF-Bpy is pH-dependent. The measured *E*_1/2_ values were subsequently used to construct the Pourbaix diagram (Figure 3b, Figure S31b). Fitting the Nernst equation to the observed data points revealed three distinct linear regimes at pH 1.63–3.84, 3.84–6.54 and 6.54–13.54, with *m*/*n* ratios of 1.91, 1.16 and 0.43, where *m* and *n* represent the numbers of protons and electrons involved in the corresponding redox equilibrium. These values are roughly consistent with H^+^/e^–^ ratios of 2:1, 1:1 and 1:2 in the three pH regimes, respectively. In Figure 3b, we tentatively assigned the possible half-reaction products from neutral bpy corresponding to each *m*/*n* ratio. However, CTF-Bpy is an extended polymeric system containing multiple redox-active bpy units in proximity, and the derived *m*/*n* ratios do not necessarily reflect the redox equilibrium on a respective, single bpy unit. Therefore, the *E*_1/2_–pH analysis only confirms the involvement of proton transfer in the reduction process of CTF-Bpy, and provides a rough estimate of H^+^/e^–^ ratio involved in the (photo)­reduction.

**Figure 3:**
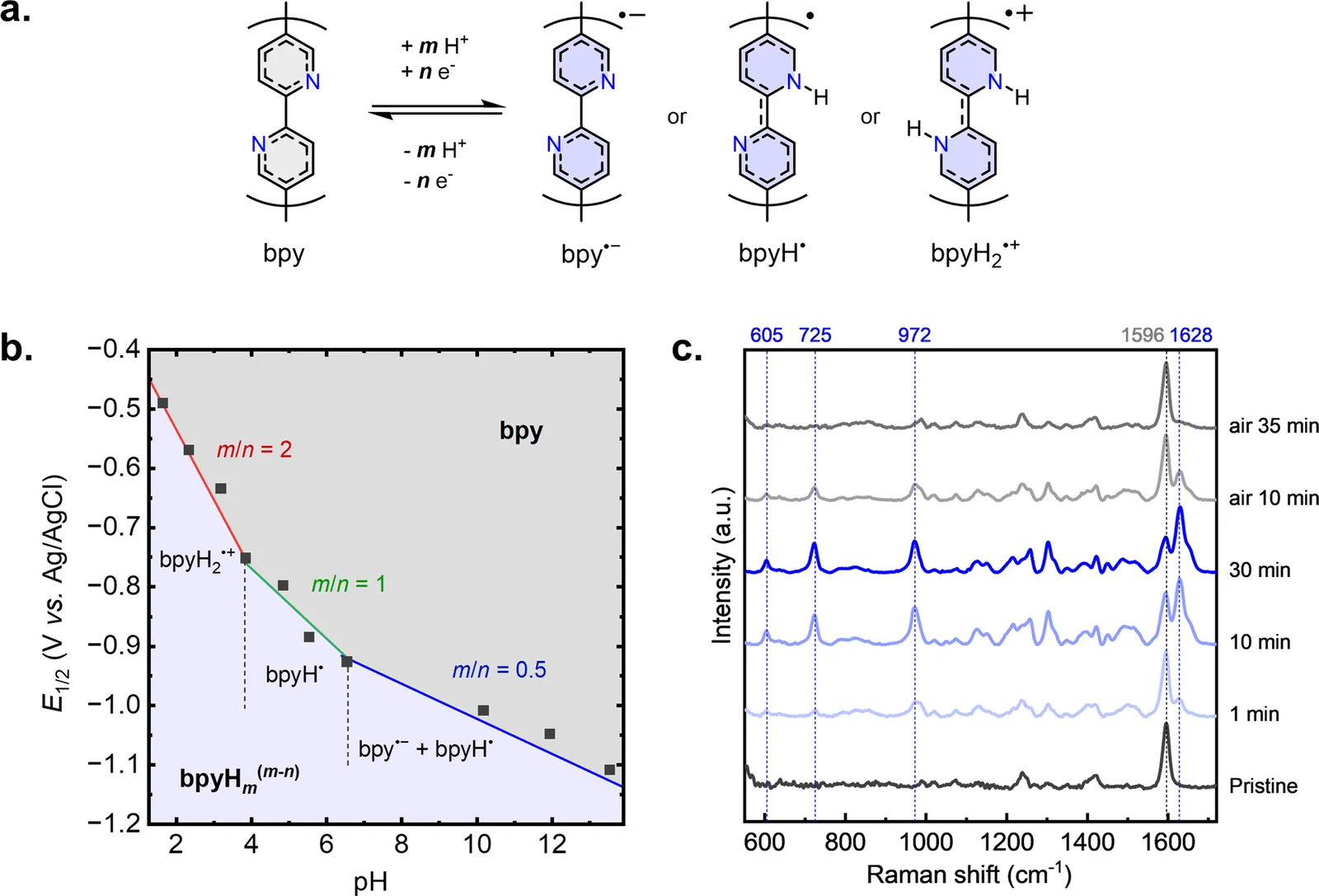
Mechanism of the photoreduction of CTF-Bpy. (a) Simplified scheme illustrating the proton-coupled reduction and possible protonation states of bpy units within CTF-Bpy. (b) Pourbaix diagram of CTF-Bpy constructed from pH-dependent CV measurements. Linear lines are overlaid as fitting with the Nernst equation. (c) Raman spectra of CTF-Bpy showing reversible radical formation under light. The CTF-Bpy aqueous suspension with 20 mM 4-MBA was measured under continuous illumination followed by air exposure. The spectra shown here are processed with background removal and normalized. Selected bands of pristine and photoreduced CTF-Bpy are indicated by gray and blue lines, respectively, with the corresponding Raman shift labeled.

As noted above, the typical photoreduction of CTF-Bpy is conducted at pH of 8.60, which falls within the pH regime associated with the 1H^+^/2e^–^ redox stoichiometry. This indicates that a proton-coupled, two-electron reduction redox equilibrium is thermodynamically accessible for CTF-Bpy under the photoreduction condition. In combination with the spectroscopic and spectro-electrochemical results in Figure 2 that support the formation of one-electron-reduced bpy species, we tentatively propose the formation of bpyH^•^ + bpy^•–^ from two bpy units within CTF-Bpy (Figure 3a–b). Given the high reported p*K*_a_ of bpyH^•^ (>14), subsequent protonation of bpy^•–^ is expected to be thermodynamically favorable under aqueous conditions. Yet, we do not exclude the possibility of other redox/protonation states, such as bpyH_2_^•+^, bpyH^–^, and bpyH_2_, as possible photoreduction products in CTF-Bpy, given the complexity arising from its polymeric nature.

Figure 3c shows the Raman spectra (785 nm excitation wavelength) of an aqueous CTF-Bpy suspension with 20 mM 4-MBA, recorded as a function of illumination time (365 nm UV light) and during subsequent air exposure. The pristine CTF-Bpy (Figure 3c, Pristine) shows the expected bands from the triazine/pyridine rings, whose full assignment is summarized in Figure S32 and Table S1. Upon illumination, we observe gradual emergence of new bands at 605, 725, 972, and 1628 cm^–1^ as well as less clear enhancement of Raman intensities in the range of 1000–1550 cm^–1^. The emergence of these new bands is accompanied by the weakening of the major band in the pristine CTF-Bpy at 1596 cm^–1^. The observed spectral changes, except for the two main bands at 1628 and 1596 cm^–1^, agree well with the reported spectral changes associated with the photoreduction of bpy to bpyH^•^.^45^ Exposing the suspension of the photoreduced CTF-Bpy to air leads to the gradual recovery of the pristine CTF-Bpy spectrum, with complete recovery after 35 min in air, indicating that the photoreduction/reoxidation and the associated local structural changes of CTF-Bpy are fully reversible.

Raman spectra of CTF-Bpy were also recorded after 30 min of irradiation under deuterated (MeOH-*d*_4_ as SED/D_2_O as solvent) and nondeuterated conditions (MeOH/H_2_O) (Figure S33). Both spectra were very similar to that obtained in the 4-MBA/H_2_O system (Figure 3c), supporting the role of methanol as SED for this system, consistent with our previous observation (Figure S14). Importantly, the most intense band at 1628 cm^–1^ in the photoreduced CTF-Bpy was red-shifted to 1612 cm^–1^ in the deuterated system, suggesting the presence of N-protonated species in the photoreduced CTF-Bpy.

DFT calculations (using ORCA 6.0.1, B3LYP/def2-TZVP) were then performed on model molecules to simulate their Raman spectra, aiding the assignment of the bands at 1596 and 1628 cm^–1^ and further elucidating the observed deuteration effect (Figure S34). As the results in Figure 2 support the formation of one-electron reduced bpy species, calculations were performed for a neutral structure model (“Neutral”) and one-electron-reduced bpy models with different protonation states, namely, one-proton one-electron-reduced (“1H1e”) and two-proton one-electron-reduced (“2H1e”) species, together with their deuterated counterparts (“1D1e” and “2D1e”). The calculated Raman spectrum of the neutral fragment agrees well with the experimental spectrum of CTF-Bpy, showing the validity of the DFT theory applied. In comparison to the experimental spectra, we found that both 1H1e and 2H1e models can explain the emergent bands in the photoreduced CTF-Bpy; the band at 972 cm^–1^ matches with the deformation modes of triazine rings bonded to the reduced bpy unit (968 cm^–1^ in 1H1e and 972 cm^–1^ in 2H1e), and the band at 725 cm^–1^ corresponds to the deformation modes of reduced bpy unit in 1H1e model. Notably, these simulated bands also appear in the deuterated models (1D1e and 2D1e), in good agreement with the consistency of these bands in the experimental spectra toward deuteration (Figure S33). Furthermore, the 2H1e model explains the highest-frequency band of photoreduced CTF-Bpy appearing at 1628 cm^–1^, and its deuterated counterpart, 2D1e, as it reproduces the observed isotopic red-shift to 1612 cm^–1^ (from 1595 to 1577 cm^–1^ in the simulated spectra). Therefore, we conclude that the majority of Raman-active species in the photoreduced CTF-Bpy are described as bpyH^•^ and bpyH_2_^•+^ (+ unreacted bpy) species, whereas their relative population remains elusive. See Supporting Information for the detailed assignment of the Raman bands (Table S1).

Based on the above results, here we propose a mechanism for the reversible photoreduction of CTF-Bpy in aqueous suspension with 4-MBA. Above-bandgap photoexcitation induces electron–hole separation in CTF-Bpy. The holes irreversibly oxidize 4-MBA to form *p*-tolualdehyde, whereas the electrons are stored and localized on the protonated reduced bpy units within CTF-Bpy, as observed from DFT calculations (Figure S35a). Photoreduction of CTF-Bpy predominantly proceeds through one-electron reduction of the bpy units. The comparison of the suspension pH before and after irradiation, quantitative analysis of the 4-MBA oxidation product, and pH-dependent CV measurements collectively suggest that the (photo)­reduction of CTF-Bpy involves proton-coupled redox chemistry, consistent with a proton-coupled electron transfer (PCET) pathway.^60^ However, whether proton and electron transfer occur in a concerted or sequential manner cannot be determined from the present data. The protons involved in the photoreduction of CTF-Bpy are predominantly supplied by 4-MBA oxidation. Combined with the experimental and simulated Raman spectra, these observations support the presence of bpyH^•^ and bpyH_2_^•+^ as the reduction species, although their relative populations remain unclear. The possible coexistence of two-electron reduced bpy species (e.g., bpyH^–^ and bpyH_2_) cannot be excluded at this stage.

After photoreduction of CTF-Bpy, it retains the reduced state for a long period of time unless it is exposed to air and being quenched by O_2_. We also monitored the radical decay kinetics in the dark under a nominally O_2_-free condition using UV–vis–NIR spectroscopy (Figure S36). We observed a mixed character of first- and second-order kinetics of the decay rate, similar to the molecular bpy system in solution.^59^ However, a more detailed kinetic analysis is necessary to substantiate the discharge mechanism. Nevertheless, EPR studies shown in Figure 2c suggest a high stability of the radicals formed from the photoreduction of CTF-Bpy; in other words, a high stability of the photocharged state of CTF-Bpy. Inspired by this, we next investigate the properties of the light-induced electrons in CTF-Bpy and explore their applications in aqueous solar batteries and dark photocatalysis.

### 2.4 (Photo)­Electrochemistry

The (photo)­electrochemical (PEC) properties of the CTF-Bpy photoanode were assessed using the same three-electrode setup described above, with Ag/AgCl (saturated KCl) as the reference electrode (Figure 4a). The photoanodes were photocharged by illuminating from the front (coated) side in an O_2_-free aqueous electrolyte with or without SED 4-MBA. First, we investigate the open circuit potential (OCP) under illumination and its dark stability in different electrolytes. As shown in Figure 4b, upon 1 min illumination, the OCP reaches −0.90 V in the electrolyte containing 4-MBA, indicating a pronounced photovoltaic effect of CTF-Bpy (Figure 4b). Under continuous illumination for 10 min, the photo-OCP value saturates at ca. −0.92 V and remains stable thereafter for more than 40 h in the dark, implying highly robust electron trapping in the framework. During photocharging in 20 mM 4-MBA aqueous electrolyte, the color of CTF-Bpy on FTO changes from yellow to black (Figure S37), suggesting one-electron reduction of bpy to occur, consistent with the spectro-electrochemical experiments described above (Figures 2, 3). In the absence of 4-MBA, the photo-OCP reaches −0.76 V after 10 min illumination in water and decays much faster, signifying water as a weak SED, in line with previous UV–vis–NIR experiments (Figure S15). This underscores the critical role of efficient hole extraction to facilitate effective charging of the CTF-Bpy photoanode and to stabilize the charged state.^4^ The transient photocurrent response of the CTF-Bpy photoanode in the absence and presence of 4-MBA are measured using chronoamperometry (CA) under dark/light cycles. As shown in Figure 4c, the photocurrent density is higher with 4-MBA than in pure water, indicating efficient hole quenching by 4-MBA. The spikes near the onset of photocurrent under illumination likely originate from recombination of photogenerated charge carriers.^61^ After three dark/light cycles, the photocurrent stabilizes at ca. 4.0 μA cm^–2^ and is reproducible in subsequent cycles, indicating stable transfer of photogenerated electrons from CTF-Bpy to the FTO upon illumination. Photocurrent responses were further examined by applying a gradually more negative bias to the photoanode (Figure 4d). During the sweep from 0 to −1.0 V, the photocurrent remains positive down to −0.9 V with 4-MBA and −0.7 V in pure H_2_O, but becomes negative at more negative potentials, suggesting the crossover from an electron injection from CTF-Bpy to the FTO substrate to the reversed injection from FTO into the material or to the electrolyte.^4^

**Figure 4:**
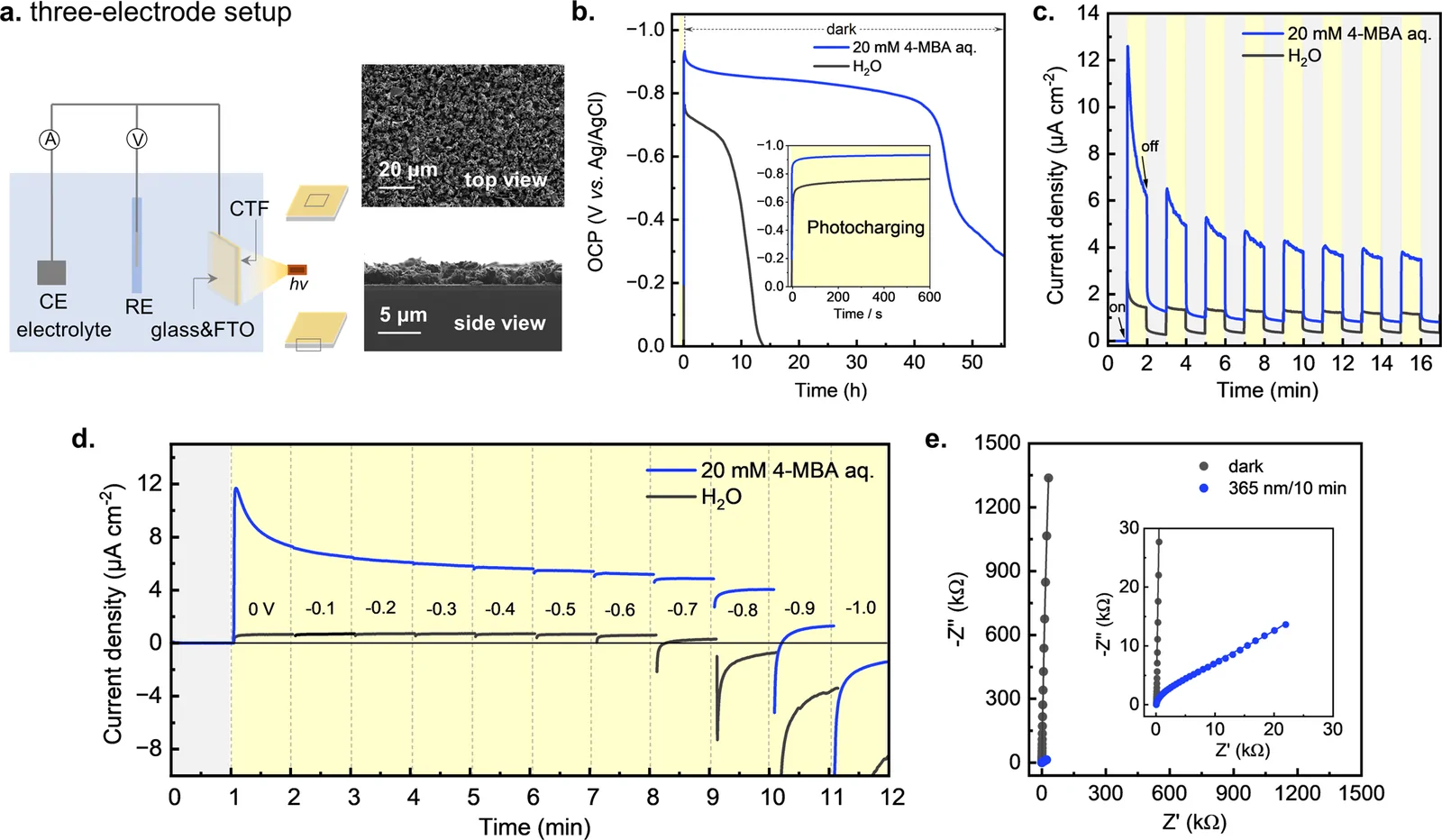
Photoelectrochemical properties of CTF-Bpy photoanode. (a) Schematic diagram of the three-electrode setup and SEM images showing the top-view and side-view of the CTF-Bpy photoanode. (b) Open circuit potential stability after 10 min 365 nm UV light illumination in the absence and presence of 20 mM 4-MBA as SED, showing the stability of stored electrons in the dark. The inset shows the OCP during 10 min photocharging. (c) Transient photocurrent response in the absence and presence of 20 mM 4-MBA. (d) Chronoamperometry measurements at different potentials under illumination in the absence and presence of 20 mM 4-MBA, which is a measure of electron generation and self-discharge current. (e) Impedance measurements in 20 mM 4-MBA aqueous solution in the ground state (dark) and in the light-induced radical state, showing significantly lower charge transfer resistance in the latter. The inset shows the magnified high-frequency region of the Nyquist plots in the dark and after illumination.

We then performed impedance spectroscopy measurements to assess the charge transfer characteristics of CTF-Bpy in the dark and under illumination. The experimental data collected before and after 10 min illumination were fitted using two equivalent circuit models (Figure 4e, Figure S38). In the dark, the equivalent circuit consists of a serial resistance (R_S_, 64.1 Ω), accounting for electrolyte resistance, in series with a parallel combination of a resistance R_C_ (1.61 × 10^8^ Ω), which will be discussed below in more detail, with a constant-phase element (CPE) representing the double-layer capacitance at the electrode/electrolyte interface.^62^ After 10 min illumination, in addition to the R_S_ and R_C_–CPE parallel elements used to fit the dark data, a charge transfer resistance (R_CT_, 3.68 × 10^3^ Ω) and a Warburg impedance (W_S_), assigned to ion diffusion (i.e., H^+^) within the electrode material, appear in parallel with a second CPE, which is utilized to reflect the emergence of the charge transfer process in the photocharged state. Upon illumination, as expected, R_S_ remains essentially unchanged (45.4 Ω), whereas R_C_ decreases by 4 orders of magnitude (1.03 × 10^4^ Ω), suggesting a significant conductivity increase. Notably, the apparent conductivity enhancement should not be interpreted as a direct measurement of the intrinsic electronic conductivity of CTF-Bpy. Rather, the substantial reduction in R_C_, together with the emergence of distinct charge-transfer and ion-diffusion contributions under illumination, indicates a pronounced change in the overall charge-transport properties of the photocharged electrode. The charge trapping process corresponds to the electronic transport channel, while ion diffusion reflects ionic/proton transport, with interfacial charge transfer and capacitive charging contributing throughout. Accordingly, the conductivity values derived from R_C_ should be interpretated as relative changes in the resistive response of the same electrode before and after illumination. Details of the fitted parameters of equivalent circuits in the dark and under light are provided in Tables S2 and S3.

### 2.5 Solar Battery Function

Encouraged by its light-induced electron-storage capability, we employed CTF-Bpy as a solar battery photoanode with the three-electrode setup described above (Figure 4a). Prior to all photocharging experiments, the photoanode was immersed into aqueous electrolyte with 4-MBA for 1 h to stabilize the OCP (ca. −0.20 V). After 10 min illumination under OCP conditions, the light was switched off and the photoanodes were discharged immediately by applying a galvanostatic discharging current (1, 1.5, 5, 10, 50, and 100 mA g^–1^). The capacity increases upon decreasing the discharge current density, and an optimum capacity of 8.2 mAh g^–1^ is achieved at 1.5 mA g^–1^, as shown in Figure 5a. Further decreasing the discharge current density to 1 mA g^–1^ causes a capacity drop to 6.7 mAh g^–1^, likely due to competitive charge loss via self-discharge of the material.^4^ Extending the illumination duration from 1 to 10 min shifts the OCP slightly more negative (−0.89 to −0.92 V) and doubles the capacity (4.1 mAh g^–1^ to 8.2 mAh g^–1^, at a discharge current density of 1.5 mA g^–1^). Further extending illumination to 30 min causes no OCP shift and only minor capacity enhancement (8.6 mAh g^–1^), indicating saturation of photogenerated charges by 10 min illumination (Figure 5b). This behavior stems from progressively slowed photocharging due to concomitant electron accumulation and recombination within the material, which limits the photocharging efficiency, as previously observed for K-PHI.^11,63^ This effect also partly explains the relatively low experimental charge storage capacity of 8.6 mAh g^–1^, which accounts for only 6.6% of the theoretical value (130 mAh g^–1^), assuming one electron is stored per bpy unit in CTF-Bpy (Figure S39). Control experiments reveal a negligible contribution of bare FTO to electron storage (Figure S40), supporting that the observed photocharging behavior, long-lived electron storage, and measured capacity of the CTF-Bpy photoanodes originate primarily from CTF-Bpy rather than the FTO substrate. Meanwhile, electrolyte-exposed FTO of CTF-Bpy photoanodes may act as a discharge channel for photoaccumulated electrons in CTF-Bpy.^4,36^ Moreover, considering the narrow optical bandgap of CTF-Bpy (2.3 eV, Figure S10), photocharging was also performed by standard solar illumination (AM 1.5 G, nominal power *P*_nominal_ ≈ 100 mW cm^–2^). Under solar illumination, the OCP rises rapidly from −0.20 to −0.77 V in 1 min, and saturates at −0.85 V after 2 h. Subsequent galvanostatic discharge at 1.5 mA g^–1^ yields a capacity of 4.7 mAh g^–1^ (Figure S41). Although this capacity is lower than that achieved by 10 min UV photocharging (8.2 mAh g^–1^), this result demonstrates the capability of direct solar-to-electrical energy conversion in CTF-Bpy.

**Figure 5:**
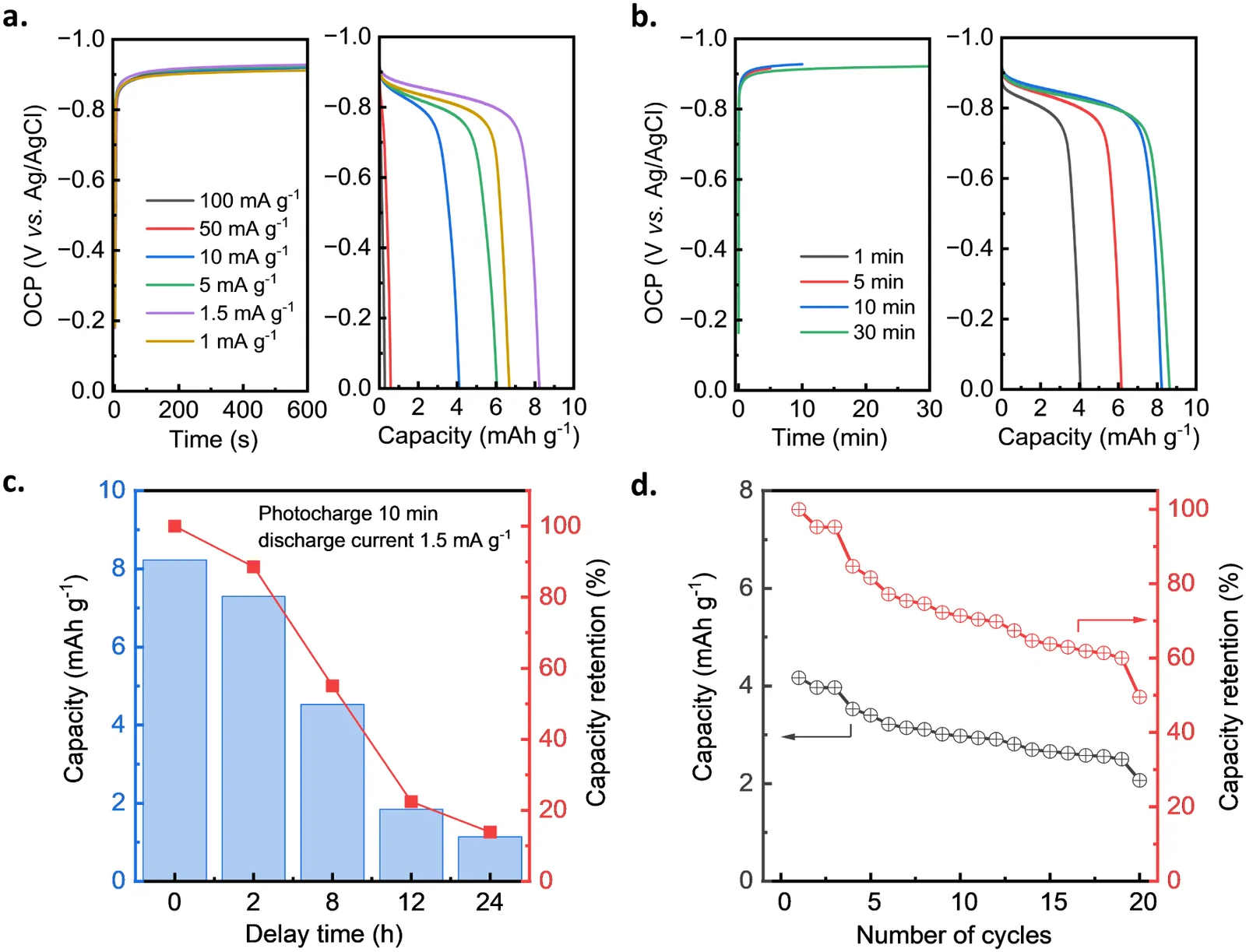
Characterizations of the solar battery half-cell with CTF-Bpy photoanode. (a) Photocharging of solar battery photoanode under 10 min 365 nm UV illumination and subsequent electric discharging under different discharge current densities. (b) Photocharging of solar battery photoanode with different illumination times and subsequent electric discharging under a fixed discharge current density of 1.5 mA g^–1^. (c) Comparison of capacity and capacity retention for delayed discharge at 1.5 mA g^–1^ after 10 min of photocharging. (d) Cycling stability of the photoanode after 1 min photocharging followed by electric discharge at a 10 mA g^–1^. The electrolyte used for PEC measurement is oxygen-free 20 mM 4-MBA aqueous solution for (a–d). Photocharging is conducted with 365 nm UV light.

Photocharging and discharging of CTF-Bpy photoanodes were also performed in 20 mM 4-MBA aqueous solution containing 0.1 M LiCl, NaCl and KCl to investigate the charge-compensation mechanism. As shown in Figure S42, neither the photocharging behavior nor the discharging capacity exhibited an apparent dependence on the presence of Li^+^, Na^+^ or K^+^, further supporting a predominantly proton-mediated charge compensation mechanism in CTF-Bpy, consistent with our mechanistic studies above. We then investigated the photocharging of CTF-Bpy photoanodes in aqueous buffer solutions containing 20 mM 4-MBA at pH 3.81, 6.65, and 9.33. As shown in Figure S43, the photo-OCP progressively shifted toward more positive potentials with decreasing solution pH, from −0.93 V at pH 9.33 to −0.77 V at pH 6.65 and −0.60 V at pH 3.81. This pH-dependent shift in the photo-OCP is consistent with the observed shift of the electrochemical reduction potential of CTF-Bpy toward more positive potentials with decreasing pH (Figure S31). The OCP of the CTF-Bpy photoanode is constant under dark condition even in the acidic buffer solution at pH 3.81, supporting that the observed photoreduction of CTF-Bpy is primarily associated with the intrinsic redox properties of the material rather than protonation effects.

To evaluate the stability of the trapped electrons and their on-demand release, we delayed the galvanostatic discharging (at 1.5 mA g^–1^) by a specified time interval after UV photocharging at 365 nm for 10 min (Figure 5c, Figure S44). After a 2 h delay, 89% of the capacity was retained, and 55% after 8 h, indicating relatively stable electron storage in CTF-Bpy and controllable release for solar battery applications. Even after 24 h, during which the OCP decreases from −0.92 V to −0.83 V, 14% of the capacity is retained. As suggested previously, Faradaic charge transfer from the partially uncovered FTO substrate (Figure S24) to the electrolyte may contribute to the time-dependent capacity decay.^4,36^

We then evaluated the cycling stability of the CTF-Bpy photoanode by repeating 1 min photocharging (365 nm) followed by galvanostatic discharging at 10 mA g^–1^ for 20 cycles (Figure 5d, Figure S45). Note that, at this current, the photoanode could not be fully discharged to 0 V, owing to intrinsic resistive losses arising from limited conductivity under high-current conditions, consistent with previous observations for the K-PHI photoanode.^4^ Therefore, after each discharge cycle the photoanode was removed from the electrolyte and exposed to air to complete discharging. Even after 20 photocharge–discharge cycles, 50% capacity was retained (Figure 5d), highlighting the relative robustness and reversibility of CTF-Bpy as an electron reservoir for solar battery applications. Comparing of the SEM images of the CTF-Bpy photoanode before and after cycling reveals increased exposure of the FTO substrate (Figure S46), suggesting partial delamination of CTF-Bpy during the cycling, which may contribute to the observed performance decay.

### 2.6 Dark Photocatalysis

Controlled release of light-induced trapped charges in the dark can potentially be exploited for dark photocatalysis, helping overcome the intermittency of solar irradiation and challenges of chemical fuel storage (especially H_2_) via on-demand release. In electron-storage materials, dark photocatalysis typically involves generating and storing photoelectrons under illumination, followed by their release in the dark, for example to reduce O_2_ to H_2_O_2_ ^32^ or H^+^ to H_2_, the latter typically triggered by addition of a Pt cocatalyst.^7^ Motivated by the above results demonstrating the capability of CTF-Bpy for stable electron storage in the dark, we evaluated its performance for H_2_O_2_ production and H_2_ evolution in the dark using our previously reported experimental setup (Figure S47).^7,20^

#### 2.6.1 Dark H_2_O_2_ Production

As mentioned above, during the UV–vis–NIR experiments, we observed H_2_O_2_ formation after quenching the photocharged CTF-Bpy suspension by air (Figure S13), which prompted us to examine the dark H_2_O_2_ production performance of CTF-Bpy. Photocharging of CTF-Bpy was performed by irradiating an O_2_-free aqueous CTF-Bpy suspension with 20 mM 4-MBA for 36 min (365 nm), followed by O_2_ purging into the suspension at a flow rate of 100 mL min^–1^ in the dark (Figure 6a). Prior to illumination, a small amount of H_2_O_2_ (108 μmol g^–1^) was present in the suspension; this could be reduced by preparing the sample in the dark under Ar (Figure S48), likely because the material is already active under ambient light and O_2_. Upon O_2_ introduction after the photocharging, the suspension rapidly changed from black to the pristine yellow color (inset of Figure 6a), indicating fast discharge, which is also reflected by significant H_2_O_2_ production in the system. The H_2_O_2_ concentration reached 1100 μmol g^–1^ within 12 min after O_2_ introduction and then remained constant over the next 2 h under continuous O_2_ flow in the dark, suggesting complete consumption of the stored electrons by O_2_ in 12 min. We then investigated the dependence of dark H_2_O_2_ production on the irradiation sources by photocharging CTF-Bpy for 36 min using 405, 505 nm LED and a full-spectrum solar simulator, and compared the results with those obtained using a 365 nm LED. As shown in Figure S49, after switching off the light and purging with O_2_ for 12 min in the dark, the amount of H_2_O_2_ produced followed the order 365 nm > 405 nm > solar simulator, yielding 1109, 598, and 497 μmol g^–1^, respectively. In contrast, photocharging of CTF-Bpy with 505 nm irradiation was unsuccessful. These results indicate that the 365 nm LED is the most effective irradiation source for photocharging CTF-Bpy, likely because its emission closely matches the absorption maximum of CTF-Bpy (Figure S49b). The cycling stability of CTF-Bpy for dark H_2_O_2_ production was evaluated over three consecutive cycles. As shown in Figure S50, CTF-Bpy could be repeatedly photocharged in each cycle, while the amount of H_2_O_2_ produced remained nearly constant across the three cycles, indicating good stability of CTF-Bpy for repeated dark H_2_O_2_ production.

**Figure 6:**
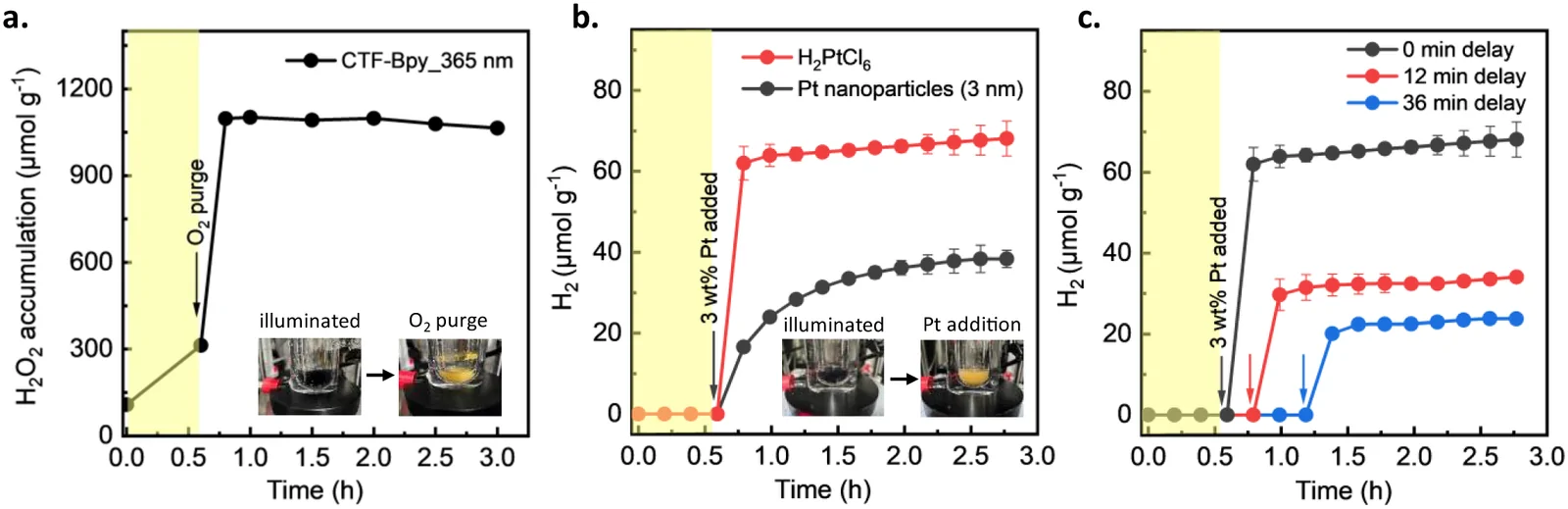
Dark photocatalytic performance of CTF-Bpy. (a) H_2_O_2_ production in the dark after purging the photocharged CTF-Bpy suspension with O_2_. The inserts show the color reversal of the CTF-Bpy suspension from black to yellow after O_2_ purge. (b) H_2_ evolution as a function of time with 3 wt % Pt as the cocatalyst. Pt cocatalyst is provided by H_2_PtCl_6_ and Pt nanoparticles, respectively. The inserts show the color reverse of CTF-Bpy suspension from black to yellow after Pt addition. (c) H_2_ evolution in the dark with various delay times. Error bars represent the averages and standard deviations of two independent experiments from two batches of CTF-Bpy. The amount of evolved H_2_ is normalized per gram of CTF.

To probe whether dark H_2_O_2_ production from O_2_ reduction proceeds via a stepwise 1e^–^ pathway in the overall 2e^–^ reduction or via a direct 2e^–^ route (Figure S51), we performed a control experiment using *p*-benzoquinone (*p*-BQ)—a known O_2_^•–^/^•^OOH radical scavenger—as O_2_^•–^ is a characteristic intermediate of the stepwise 1e^–^ pathway.^64^ Although no H_2_O_2_ was formed in the presence of *p*-BQ, this result alone does not confirm a stepwise 1e^–^ pathway, as *p*-BQ suppresses photocharging of CTF-Bpy by scavenging photogenerated electrons and undergoing self-reduction to hydroquinone (Figures S52, S53). A spin-trapping experiment using 5,5-dimethyl-1-pyrroline N-oxide (DMPO) was further performed to probe the possible formation of O_2_^•–^ and/or its protonated form, ^•^OOH, as their corresponding DMPO adducts are expected to exhibit characteristic six-line EPR spectra (Figure S54).^65^ In a CTF-Bpy aqueous suspension containing 20 mM 4-MBA and 100 mM DMPO, only the EPR signal of CTF-Bpy was observed after irradiation under Ar. Upon introducing air in the dark, no six-line signal corresponding to the DMPO-O_2_^•–^/DMPO-^•^OOH adduct was observed. Instead, a characteristic four-line EPR spectrum with an intensity ratio of approximately 1:2:2:1 was detected, consistent with the formation of DMPO-^•^OH. This detection of DMPO-^•^OH may suggest a stepwise 1e^–^ pathway for the dark H_2_O_2_ formation, as the DMPO-O_2_^•–^/DMPO-^•^OOH adduct has been reported to undergo rapid decay to DMPO-^•^OH in aqueous media.^66,67^

#### 2.6.2 Dark Hydrogen Evolution

As shown earlier, the photo-OCP of CTF-Bpy photoanode reaches −0.92 V vs Ag/AgCl (−0.72 V vs NHE) in 20 mM 4-MBA aqueous electrolyte upon 10 min 365 nm UV illumination (Figures 4b, 5b), corresponding to the one-electron reduction of the bpy unit. Based on the H^+^/H_2_ redox potential of −0.41 V vs NHE (pH 7, 25 °C),^39^ the one-electron reduced species is thermodynamically capable of driving the H_2_ evolution reaction (with a redox potential difference of ΔE ≈ 0.31 V, corresponding to a Gibbs free energy of ΔG ≈ −60 kJ mol^–1^). We thus tested CTF-Bpy for H_2_ evolution in the dark.

Photocharging of CTF-Bpy follows the same procedure as for dark H_2_O_2_ production (365 nm, 36 min), but instead of O_2_, 3 wt % Pt cocatalyst was introduced into the suspension to drive H_2_ evolution in the dark. As for the cocatalyst, we tested Pt nanoparticles (∼3 nm) and H_2_PtCl_6_ (H_2_PtCl_6_·6H_2_O was used) (Figure 6b). In both cases, addition of the Pt cocatalyst after switching off the light resulted in H_2_ evolution, accompanied by the color reversal of the suspension from black to pristine yellow (inset of Figure 6b). Notably, the introduction of H_2_PtCl_6_ induces faster electron release and higher maximum H_2_ evolution as compared to Pt nanoparticles over a 3-h test (68.1 vs 38.4 μmol g^–1^, corresponding to a turnover frequency of 0.20 and 0.11 h^–1^, respectively). This can be attributed to a more uniform distribution of Pt nanoparticles (Pt^0^) across CTF-Bpy, enabled by i*n situ* reduction of H_2_PtCl_6_ (Pt^4+^), although the reduction itself also consumes electrons.^68,69^ To distinguish the amounts of stored electrons consumed by H_2_ evoution and H_2_PtCl_6_ reduction, inductively coupled plasma (ICP) analysis was performed on CTF-Bpy after the dark H_2_ evolution experiment. While 3 wt % of Pt, supplied by H_2_PtCl_6_, was added to the system, ICP analysis of the recovered CTF-Bpy revealed a Pt content of 2.16 wt %. As TEM images show that the generated Pt nanoparticles (Pt^0^) remain associated with the recovered CTF-Bpy (Figure S55), and assuming that all unreacted H_2_PtCl_6_ was removed during water washing (H_2_PtCl_6_ is highly water-soluble), the measured Pt content corresponds to an estimated consumption of 443 μmol e^–^ g^–1^ CTF-Bpy for Pt^4+^ reduction to Pt^0^.

Next, we investigated the dark H_2_ evolution of CTF-Bpy in detail with 3 wt % H_2_PtCl_6_ as the cocatalyst. By varying the illumination time, the maximum H_2_ production was achieved at 36 min illumination, with no further increase upon extending the illumination time to 1 h (Figure S56). After the dark H_2_ evolution follows 1 h of irradiation, we observed a decrease in the intensity of the first peak ({100} plane) (Figure S57a). Since the {100} reflection in 2D layered COFs/CTFs represents in-plane periodicity, this decrease is most likely associated with the decrease in the stacking order between layers under the reaction conditions (i.e., slippage of layers), which affects the out of plane pore arrangement.^70^ It should be also noted that the {001} reflection at around 26.4° did not change before and after the dark H_2_ evolution, which further indicates that the stacking distance was maintained. Nevertheless, despite the slight decrease in crystallinity, TEM supports the local retention of the CTF-Bpy framework structure (Figure S58a–c), while FTIR measurements confirm that its chemical structure is retained after the dark H_2_ evolution experiment (Figure S57b). The corresponding Energy-dispersive X-ray (EDX) analysis further supports that the generated Pt nanoparticles from H_2_PtCl_6_ reduction remain associated with the material after the dark H_2_ evolution experiment (Figure S58d).

Inspired by the stability of the stored electrons in the solar battery photoanode (Figure 5c), we also tested dark H_2_ production by delaying the addition of 3 wt % H_2_PtCl_6_ after 36 min UV illumination. Delaying Pt cocatalyst addition by 12 and 36 min yielded maximum dark H_2_ production of 34.1 and 23.8 μmol g^–1^, corresponding to 50.1% and 34.9% of the undelayed performance (68.1 μmol g^–1^), respectively (Figure 6c). While the performance loss upon delayed Pt addition likely arises from radical quenching via O_2_ (i.e., possible air leaking into the suspension) and self-decay^59,71^ during the waiting period, the delayed experiments nonetheless indicate stable charge retention, highlighting the potential of CTF-Bpy for controlled, on-demand H_2_ production in the dark. Preloading Pt onto CTF-Bpy inhibits the efficient accumulation of electrons under illumination because the photogenerated electrons are competitively and immediately consumed for H_2_ evolution, as observed both in system with H_2_PtCl_6_ (Figure S59), and the system with Pt nanoparticles as the cocatalyst (Figure S60). CTF-Bpy represents the first example of a COF that is capable of driving H_2_ evolution in the dark (Table S4), thereby opening this rich materials library for the rational development of more efficient COFs-based systems for dark photocatalysis.

### 2.7 The Number of Accumulated Electrons in CTF-Bpy

To estimate the fraction of bpy units involved in light-induced electron accumulation in CTF-Bpy, we compared the number of accumulated electrons determined by spectroscopic methods (EPR and UV–vis) and functional applications (solar battery and dark H_2_O_2_/H_2_ production) with the theoretical maximum. Quantitative EPR measurements were performed to determine the spin concentration of photocharged CTF-Bpy. By fitting the measured EPR intensity to a calibration curve established using 4-hydroxy-2,2,6,6,-tetramethylpiperidine-1-oxyl (Tempol), the electron storage amount was determined to be 0.10 mmol g^–1^ (i.e., 0.10 mmol e^–^ per gram of CTF) (Figures S61–S 62). Using methyl viologen (MV^2+^) as an electron acceptor, the accumulated electrons in photocharged CTF-Bpy were quantified by monitoring MV^2+^ reduction using UV–vis spectroscopy, yielding an electron storage amount of 0.285 mmol g^–1^ (Figure S63). For the functional applications, the number of accumulated electrons was estimated from the maximum performance achieved in each case. Accordingly, electron-storage amounts of 0.306 mmol g^–1^, 2.20 mmol g^–1^ and 0.579 mmol g^–1^ were estimated from the solar battery discharge capacity (8.2 mAh g^–1^), dark H_2_O_2_ production (1100 μmol g^–1^), and dark H_2_ evolution (68.1 μmol g^–1^ H_2_ plus 0.443 mmol g^–1^ for H_2_PtCl_6_/Pt^4+^ reduction to Pt^0^), respectively. Assuming one-electron reduction of each bpy unit in CTF-Bpy, the theoretical maximum electron-storage capacity is 4.85 mmol g^–1^ (Figure S39). Thus, the electron-storage amounts determined by quantitative EPR, MV^2+^ reduction, solar battery discharge, dark H_2_O_2_ production, and dark H_2_ evolution correspond to 2.06%, 5.88%, 6.31%, 45.36%, and 11.94% of the theoretical maximum, respectively. These results are summarized in Table S5 in the Supporting Information.

The relatively lower electron-storage values estimated from MV^2+^ reduction, solar battery discharge, and dark H_2_ evolution may be partly related to possible air/O_2_ leakage. The substantially lower value obtained from quantitative EPR may additionally reflect underestimation of the total electron population due to EPR-silent two-electron reduced species, whose presence cannot yet be excluded. Among the different approaches, dark H_2_O_2_ production yielded the highest estimated electron-storage amount, partly due to the undesired O_2_ quenching in other applications directly contributed to H_2_O_2_ formation. As the dark H_2_O_2_ production was initiated immediately after irradiation without delay, we propose that this method provides the closest estimate of the actual electron-storage capacity of CTF-Bpy under irradiation. Assuming one-electron reduction per bpy unit, this value corresponds to approximately 45% of the bpy units in CTF-Bpy participating in electron storage upon irradiation.

## 3 Conclusion

Herein, we report and systematically investigate the light-induced electron storage properties of a known COF, CTF-Bpy, and explore their utility for aqueous solar batteries and dark photocatalysis. The light-induced electron accumulation is reversible and is quenched by exposure to air/O_2_. A combination of spectroscopic, electrochemical, and spectro-electrochemical experiments, together with DFT calculations, reveals that the electron storage arises from the one-electron reduction of the redox-active bpy unit via a proton-coupled process, with bpyH^•^ and bpyH_2_^•+^ identified as the photoreduction product. The photogenerated electrons stored in CTF-Bpy can be released on demand by electrical discharge in the dark, delivering capacities up to 8.2 mAh g^–1^ when employed as a photoanode for aqueous solar battery application. The stored electrons can be released by reaction with O_2_ to produce H_2_O_2_ in the dark, with a maximum production of 1100 μmol g^–1^. Moreover, enabled by the sufficiently negative photo-OCP (−0.92 V vs Ag/AgCl), the photocharged CTF-Bpy can drive dark H_2_ evolution with a maximum production of 68.1 μmol g^–1^ in the presence of Pt cocatalyst, representing the first example of COFs for dark H_2_ evolution. Comparison of the number of accumulated electrons estimated from different spectroscopic methods and functional applications reveals that dark H_2_O_2_ production yields the highest estimated electron-storage capacity. Assuming that only one-electron reduction of the bpy units occurs in photoreduced CTF-Bpy, this value corresponds to approximately 45% of the bpy units undergoing one-electron reduction under irradiation.

This systematic investigation of light-induced electron storage in CTF-Bpy establishes a general protocol for studying charge accumulation in related systems and highlights the potential of structurally diverse COFs as bifunctional materials. Moreover, the inherent molecular tunability of COFs is advantageous to elucidate the structure–property–function relationships for the rational design of more stable, sacrificial reagents free, multielectron/hole storage under visible light irradiation, advancing efficient direct solar-to-electrochemical/chemical energy storage to mitigate solar intermittency in future energy systems.

## Supplementary Material



## Notes

Detailed experimental methods, along with corresponding supporting Figures S1–S63 and supporting Tables S1–S5 mentioned in the main text (PDF)
