# Supplementary material for: Direct Light Storage with Controlled Dark Release in a Bipyridine-Based Covalent Triazine Framework
Source: J Am Chem Soc. 2026 Sep 26;148(39):42121–35. doi: 10.1021/jacs.6c09757 (PMC13651528; doi:10.1021/jacs.6c09757)
Supplement: Supplementary file 1 [file ja6c09757_si_001.pdf]

Supporting Information for

Direct Light Storage with Controlled Dark Release in a Bipyridine-based Covalent Triazine Framework

Xue Wang,*[1] Bibhuti Bhusan Rath,[1] Nicolai Schröter,[2,3] Kristina Gjorgjevikj,[1,4] Rohit Mann,[1] Shun Tokuda,[1] Yang Wang,[1] Viola Duppel,[1] Lorenzo Tesi,[2,3] Simon Krause,[1,4] Bettina V. Lotsch*[1,5,6]

[1] Nanochemistry Department, Max Planck Institute for Solid State Research, 70569 Stuttgart, Germany

[2] Institute of Physical Chemistry, University of Stuttgart, Pfaffenwaldring 55, 70569, Stuttgart, Germany

[3] Center for Integrated Quantum Science and Technology (IQST), University of Stuttgart, Pfaffenwaldring 57, 70569 Stuttgart, Germany

[4] Institut für Anorganische Chemie II, Universität Ulm, Albert-Einstein-Allee 11, 89081 Ulm, Germany

[5] Department of Chemistry, Ludwig-Maximilians-Universität (LMU), 81377 Munich, Germany

[6] E-Conversion and Center for Nanoscience, 85748 Munich, Germany

Table of Contents

1. Materials and Methods 3

1.1 Nuclear magnetic resonance (NMR) spectroscopy 3

1.2 Elemental Analysis 3

1.3 High-performance liquid chromatography 3

1.4 Powder X-ray diffraction 3

1.5 Fourier-transform infrared spectroscopy 4

1.6 Gas sorption analysis 4

1.7 Scanning electron microscopy 4

1.8 Transmission electron microscopy 4

1.9 Thermogravimetric analysis 4

1.10 Ultraviolet-visible-NIR spectroscopy 4

1.11 Electron paramagnetic resonance spectroscopy 4

1.12 Raman spectroscopy 5

1.13 DFT calculations 5

1.14 (Photo)Electrochemical measurements 6

1.15 Dark photocatalysis 6

2. Synthetic procedures 8

2.1 Synthesis of 2,2'-bipyridine-5,5'-dicarboximidamide dihydrochloride 8

2.2 Synthesis of CTFs 10

3. Characterization of CTF-Bpy 12

4. Reversible light-induced radical formation 18

4.1 Band gap of CTF-Bpy 18

4.2 UV-vis-NIR spectra 19

4.3 Characterization of CTF-Bph 26

4.4 Cyclic voltammetry measurements 30

4.5 *In situ* EPR spectro-electrochemistry measurements 35

5. Mechanistic investigation of the light-induced radical formation 37

5.1 PXRD under illumination 37

5.2 Raman spectra 40

6. Photoelectrochemistry investigation 46

7. Solar battery function 48

8. Dark photocatalysis 56

9. Structure modelling 74

10. References 78

1. Materials and Methods

The following chemicals were purchased from commercial sources and used in this work without further purification. 2,2'-bipyridine-5,5'-dicarbaldehyde (BLD, 97%), 2,2'-bipyridine-5,5'-dicarbonitrile (BLD, 97%), 1,1'-biphenyl-4,4'-bis(carboximidamide) dihydrochloride (BLD, 97%), 1,1'-biphenyl-4,4'-dicarbaldehyde (BLD, 95%), lithium bis(trimethylsilyl)amide solution (1 M in THF, Sigma-Aldrich), acetyl chloride (Alfa Aesar, 98%), Cs_2CO_3 (Sigma-Aldrich, 99%), potassium chloride (KCl, ROTH, ≥99%), 4-methylbenzyl alcohol (4-MBA, TCI, >99.0%), triethanolamine (TEOA, Sigma-Aldrich), methanol (VWR, ≥99.8%). methanol-d_4 (CD_3OD, Sigma-Aldrich, ⩾99.8 atom % D), meuterium oxide (D_2O, Sigma-Aldrich, 99.9 atom % D). The fluorine doped tin oxide (FTO) coated glass slide (735159-5EA, Sigma-Aldrich), nafion (Ion Power, D521CS), carbon black (Vulcan XC72R, QUINTECH), PTFE (powder (free-flowing), 1 μm particle size, Sigma-Aldrich), N-methyl-2-pyrrolidone (NMP, Acros Organics, 99% extra pure). Pt nanoparticles (3 nm, 1000 ppm, US Research Nanomaterials, Inc, 99.99%), chloroplatinic acid hexahydrate ($H_2PtCl_6.6H_2O$, Sigma-Aldrich, ≥37.50% Pt basis). peroxide test strips, colorimetric (Sigma-Aldrich). anhydrous tetrahydrofuran (Sigma-Aldrich, ≥99.9%), anhydrous EtOH (Sigma-Aldrich). Other non-anhydrous solvents: DMSO (Fisher Scientific), 1, 2-dichlorobenzene (Sigma-Aldrich, ≥98%), DMF (Sigma-Aldrich), tetrahydrofuran (THF, Sigma-Aldrich), acetone (VWR), isopropanol (VWR), EtOH (ROTH), distilled water was used for washing, milli-Q water was used for electrochemical test.

1.1 Nuclear magnetic resonance (NMR) spectroscopy

Solution NMR spectra were recorded on a JEOL ECZ 400S 400 MHz spectrometer at 25 °C, operating at frequencies of 400 MHz (^1H) and 100 MHz (^{13}C) and referenced against the residual ^1H or ^{13}C signal of the solvent (DMSO-d_6).

Solid-state proton magic-angle spinning (^1H MAS) and carbon-13 cross-polarization/magic-angle spinning (^{13}C CP/MAS) NMR spectra were recorded at room temperature in 3.2 mm OD ZrO_2 rotors on a Bruker Avance III 400 MHz and Neo 600 MHz spectrometers using Bruker BL3.2 double resonance MAS probes. Standard instrument library pulse sequences were used. Chemical shifts were referenced relative to Tetramethylsilane (TMS) (^1H, 0.0 ppm; ^{13}C, 0.0 ppm).

1.2 Elemental Analysis

CHN analysis was performed on an Elementa vario EL using Helium as carrier gas. Cs content in CTFs was confirmed by analytical service from Pascher (analytical lab in Remagen, Germany).

1.3 High-performance liquid chromatography

High-performance liquid chromatography (HPLC) measurements were conducted on an UHPLC with DAD-UV-detector from Agilent using a C_{18} column. Mobile phase A was 0.1% formic acid in water and mobile phase B was 0.1% formic acid in acetonitrile. The gradient program was applied as follows: 0-1 min, 90% A; 1-5 min, 90-10% A; 5-6 min, 10% A; 6-7 min, 10-90% A. Flowrate was 0.6 mL/min. All samples were prepared in miliQ water.

HPLC Calibration: Standard solutions of *p*-tolualdehyde at concentrations of 0.1, 0.25, 0.5, 1 and 2 mM were prepared by serial dilution of a 5 mM stock solution in HPLC grade acetonitrile. Each standard was injected into the HPLC system using an injection volume of 1 µL. The chromatographic peak areas were recorded and automatically integrated using the HPLC software (ChemStation). A calibration curve was generated by plotting peak area against analyte concentration, and linear regression analysis was performed to obtain the calibration equation used for quantitative analysis.

1.4 Powder X-ray diffraction

PXRD patterns were collected using a Stoe Stadi P diffractometer with a Cu Kα1 source monochromatized with Ge(111) in a Debye-Scherrer geometry at room temperature (RT). Each sample was securely sealed in glass capillaries with inner diameter of 1.0 mm, and the measurements were performed with spinning to enhance particle statistics. PXRD patterns were measured over the 2θ range 1.5-40° over 1 hour. For the preparation of the photocharged sample for PXRD measurement, the capillary was prepared inside the glovebox from a CTF aqueous suspension with 20 mM 4-MBA. Photocharging of the sample was conducted

by directly illuminating the sealed capillary outside of the glovebox. The capillary was rotated during the illumination to ensure uniform photocharging of the sample.

1.5 Fourier-transform infrared spectroscopy

IR spectra were obtained in attenuated total reflection (ATR) geometry using a PerkinElmer UATR Two apparatus equipped with a diamond crystal. The measurements were conducted with a resolution of 4 cm^{-1}. The spectra were corrected for the ATR effect (contact parameter: 0), subtracted with automatically determined baselines, and normalized to the highest intensity peak.

1.6 Gas sorption analysis

Nitrogen sorption measurements were conducted at 77 K using a Quantachrome Instruments Autosorb iQ 3. The BET surface area calculations from the nitrogen sorption isotherms were performed by using the BETSI program.[1] The pore size distribution (PSD) was determined by employing the N_2 adsorption data at 77 K and applying the QSDFT model (cylindrical pores, adsorption branch) within ASiQwin software version 3.01. Prior to measurement, the CTF samples were subjected to activation under high vacuum conditions at 120 °C for a duration of 12 hours.

1.7 Scanning electron microscopy

SEM images of CTFs were recorded on a Zeiss Merlin microscope under the electron voltage of 1.5 kV.

1.8 Transmission electron microscopy

HR-TEM images were collected using a Philips CM30 ST instrument operating at 300 kV with a LaB6 cathode. The samples were distributed onto a holey carbon/copper grid. Images were taken with a TVIPS TemCam-F216 CMOS camera. Image analysis and fast Fourier transform (FFT) were performed with DigitalMicrograph software.

1.9 Thermogravimetric analysis

TGA were performed out on a TG50 analyzer (Mettler-Toledo) and a SDT Q600 TG-DTA analyzer in Ar at a heating rate of 10 °C min^{-1} within a temperature range of 30 - 950 °C.

1.10 Ultraviolet-visible-NIR spectroscopy

UV-vis-NIR spectra were recorded on a Cary 5000 UV-vis-NIR spectrophotometer (Agilent) with a 1 cm cuvette at room temperature. Unless otherwise specified, UV-vis-NIR spectra were recorded by dispensing 1 mg of CTFs in 3 mL water with, or without the presence of a sacrificial electron donor.

UV-vis-NIR spectro-electrochemical experiments were conducted by combining Metrohm Autolab potentiostat (PGSTAT302) with Nova 2.1.4 software electrochemistry setup with the above spectrophotometer. A cuvette was employed as the electrochemical cell, with Pt wire as pseudo-reference and 0.5 M KCl aqueous solution as the electrolyte, all other conditions remained the same as above. The electrode for (photo)electrochemical measurements was used for this measurement after cutting to fit the cuvette.

1.11 Electron paramagnetic resonance spectroscopy

EPR measurements were conducted on a Bruker EMX spectrometer at room temperature with X-band frequencies. A Bruker ER 4105DR Double rectangular resonator with a TE_{104} mode equipped with an Oxford Instruments ESR900 Cryostat was used except for the illumination saturation experiment, where a Bruker ER 4102ST Standard Rectangular resonator with a TE_{102} mode was used. The magnetic field shift of the spectrometer was determined by using DPPH. The spectrum fitting was conducted with the EasySpin (Version 6.0.6) software package[2] for Matlab (Version R2024b) using a spin Hamiltonian with isotropic g tensors ($H = \mu_B \boldsymbol{SgB}_0$) and a Voigtian line broadening for the illumination experiments and a spin Hamiltonian with anisotropic g tensor and anisotropic Gaussian line broadening for the SEC experiments.

The electrochemical cell for the in situ EPR spectro-electrochemistry was a basic three electrode setup of Pt/Pt/AgCl fitted into an EPR tube under Ar atmosphere with 0.5 M aqueous KCl electrolyte.

Electrochemical potentials were applied using a Keithley 2450 SMU and a home-written program in Labview for cyclovoltammetry experiments.

Sample preparation. The Ar-purged suspension of CTF is put in a 2 mm (outer diameter) glass capillary, which was then transferred to the bottom of a capped 4 mm quartz EPR tube (outer diameter) for the measurement. Sample transfer to the EPR tube was performed under Ar protection. Photocharging was performed by directly illuminating the CTF suspension in the EPR tube. During the radical lifetime measurement, the capped EPR tube was stored in air.

Preparation of the CTF-Bpy working electrode for the EPR spectro-electrochemistry measurements was performed by dip-coating method to coat CTF-Bpy onto the Pt wire. 5 mg of CTF-Bpy dispersed in a mixture of 80 μL NMP and 20 μL Nafion (5 wt%) was used for dip-coating. The dip-coating was repeated for 6 times with 1 h interval in between to get dried. The prepared electrode was then put to dry inside a fume hood for 2 days before the measurement.

Quantitative EPR and the data analysis. Quantitative EPR measurements were performed using a Bruker ER 4105DR double rectangular resonator equipped with an Oxford Instruments ESR900 Cryostat in the main sample chamber. The second sample chamber was loaded with a powder ultramarine standard sample. A calibration curve was created by measuring aqueous solutions of 4-hydroxy-2,2,6,6-tetramethylpiperidin-1-oxyl (Tempol) radical with concentrations between 0.01 and 1 mM. To account for changes in the Q-factor, for every sample, the ultramarine standard in the second sample chamber was measured just before or after the sample in the same tuning conditions. The intensity is obtained from the double integration of the simulated EPR signals, and experimental deviations due to the tuning condition of the resonator were compensated by normalizing the obtained intensity to that of the corresponding ultramarine standard.

For quantitative EPR measurements, the mass of CTF-Bpy loaded into the capillary was calculated from the concentration of the CTF-Bpy suspension and the volume of suspension introduced into the capillary.

Spin trap experiments. For the spin trap experiments, the CTF samples were prepared in the same way as for the normal EPR experiments with illumination (see section 1.11 in the SI), except that DMPO (100 mM) was added to the suspension under Nitrogen as well, so DMPO was already present during the illumination. After illumination, the glass capillary was exposed to air and additional air was purged through the sample with a syringe to induce the electron release, before the sample was inserted again into the resonator and measured as quickly as possible, approximately after 1-2 minutes.

1.12 Raman spectroscopy

All Raman spectra were recorded on a home-built system equipped with a Coherent TR-Raman probe (785 nm excitation laser and 300 mW power at sample port) connected to an Andor Kymera 328i spectrograph with an Andor iDUS 420 CCD detector (1200 g/mm grating). No more than 40 % of laser power was used in the measurements to reduce the possibility of sample degradation, which was not detected in any measurements. The measurements were carried out in a closed quartz cuvette.

All spectra have been processed by removal of cosmic rays, baseline corrected and normalized to the highest intensity peak using Spectragryph 1.2 by Spectroscopy Ninja. This has not affected the signals disposition and all extracted information are valid to the raw data.

1.13 DFT calculations

DFT calculations were performed using ORCA 6.0.1 at the B3LYP/def2-TZVP level of theory. Specifically, DFT calculations were performed with a molecular fragment of CTF-Bpy containing five bipyridine units and two triazine units, and protonation was introduced only on the central bpy unit for the sake of stable convergence of the calculations. For representing the pristine (bpy) and protonated one-electron reduced bpy species (bpyH$^{\bullet}$ and bpyH$_2^{\bullet+}$), calculations were carried out for a neutral structure (“Neutral”), one-proton one-electron-reduced (“1H1e”), two-proton one-electron-reduced (“2H1e”), and their deuterated counterparts (“1D1e”, “2D1e”) by assigning corresponding number of H/D atoms, system charges, and spin multiplicity (**Figure S34a**).

1.14 (Photo)Electrochemical measurements

Electrode preparation. A suspension of 5 mg of CTF-Bpy in 0.5 mL *N*-methyl-2-pyrrolidone (NMP) was vigorously stirred at room temperature for 12 h, yielding a dispersion concentration of 10 mg/mL. To prepare the CTF-Bpy ink for drop-casting, the dispersion was diluted by NMP to obtain an ink of 35 µg/10 µL. Then, 20 µL of this CTF-Bpy ink was drop-casted (at once) onto a precleaned (acetone × 3, isopropanol × 3, each time with 10 min sonication), dry, FTO coated glass substrate at a dimension of 12 × 10 mm. The mass loading of CTF-Bpy on each FTO coated glass substrate is 70 µg. NMP was allowed to evaporate slowly inside the fume hood over 3 days and the resulted films were further dried at room temperature under air flow for 12 h, followed by 2 days in the fume hood. The obtained CTF film was scratched a little at the corner of the substrate and silver paste was used to make contact with isolated copper wire. To dry the silver paste, the films were heated in oven at 60 °C for 3 h. Afterwards, the contact was properly sealed with epoxy (3 M Scotch-Weld DP410) and the films were again dried in an oven at 60 °C for 0.5 h, leaving an exposed electrode area of approximately 10 × 10 mm^2. Unless otherwise specified, the film preparation remained same for all the (photo)electrochemistry measurements.

(Photo)electrochemical experiments. All the (photo)electrochemical measurements were performed in a custom-made closed glass reactor equipped with a quartz window for illumination. A conventional three electrode setup with RE-1CP reference electrode (Ag/AgCl/saturated KCl) and platinum wire as counter electrode (**Figure S23**). Prior to every measurement, the electrolyte was purged with >99% pure Ar through a porous glass frit to remove oxygen to ensure an O_2-free environment. Water, or 20 mM 4-MBA aqueous solution was used as electrolytes for different (photo)electrochemical measurements.

Electrochemical measurements were recorded and analyzed using a multichannel potentiostat (Autolab M204, Metrohm) and the NOVA software. The 365 nm UV illumination was provided by a Thorlabs M365LP1 LED coupled to a COP4-A collimation adapter (Zeiss) and operated at an operating current of 1700 mA. Simulated sunlight was provided by a SCIENCETECH LightLine A4 solar simulator (class AAA) fitting the ASTM standard G138 (AM 1.5G). The illumination intensity was measured using a THORLABS PM100D compact power and energy meter equipped with a THORLABS S120VC photodiode sensor for 365 nm UV illumination and a THORLABS S310C thermal power sensor head for solar illumination. The intensity was set to $P_{nominal} \approx 237$ mW cm^{-2} for the 365 nm UV irradiation (the emission is circle-shaped with a diameter of 2 cm. Inside the circle is a square-shaped emission spot, with an area of 0.7 × 0.7 cm^2.) and $P_{nominal} \approx 100$ mW cm^{-2} for the sunlight irradiation. Electrochemical impedance measurements were performed in a frequency range of 100 kHz ~ 0.01 Hz.

Cyclic voltammetry (CV) measurements in aqueous electrolyte (0.1 M KCl) were performed in a potential window of 0 ~ −1.5 V vs Ag/AgCl (saturated KCl) under continuous Ar purging. CV measurements in 0.1 M $TBAClO_4$ acetonitrile solution were performed using a WaveDriver 200 EIS Pine Bipotentiostat at a potential window of 0 ~ −2.8 V under continuous Ar purging. The experimental setup involved a three-electrode configuration, with $Ag/AgNO_3$ (0.1 M $TBAClO_4$ acetonitrile solution) serving as the reference electrode and platinum wire as the counter electrode. CV test of the ferrocene reference was performed by dissolving ferrocene in 0.1 M $TBAClO_4$ acetonitrile solution at a concentration of 5 mM with a Pt wire as the working electrode. pH was measured by Mettler-Toledo pH meter. The pH meter was calibrated before the measurements.

1.15 Dark photocatalysis

H_2O_2 production in the dark. The dark H_2O_2 production experiments were conducted in a home-made glass reactor with a quartz window on the top for illumination and thermostated at 25 °C as previously described (**Figure S47**).[3] The 365 nm UV illumination was provided by a Thorlabs M365LP1 LED coupled to a COP4-A collimation adapter (Zeiss) and operated at an operating current of 1700 mA (237 mW cm^{-2}). In a typical dark H_2O_2 production experiment, 5 mg of CTF-Bpy powder was dispersed in 5 mL 20 mM 4-MBA aqueous solution and sonicated for 10 min prior to the reaction. The reactor headspace was evacuated and refilled with argon three times to remove air (O_2). The suspension was continuously stirred at 300 rpm throughout the reaction. During measurement, the reactor was illuminated from the top for a specified duration (36 min), after which the light was turned off and oxygen (O_2) was purged into the suspension at a purging rate of 100 mL min^{-1} to discharge the stored electrons. The quantification of H_2O_2 was conducted through a spectrophotometric method based on the triiodide procedure, as previously reported.[4, 5]

H_2 evolution in the dark. For dark H_2 evolution, the sample preparation and photocharging procedures were the same as for dark H_2O_2 production experiments. During measurement, the reactor was illuminated from the top for a specified duration (12 min, 24 min, 36 min and 1h, respectively), after which the light was turned off and the system was allowed to stand for a particular delay period (for the delayed discharge experiments). Afterwards, a 3 wt% Pt co-catalyst aqueous solution was injected into the photocharged suspension and the system was kept under dark for the following measurements. Both the syringe and the Pt solution were Ar-purged for 10 min to minimize the O_2 content. The 3 wt% Pt co-catalyst was provided either as a Pt nanoparticle aqueous dispersion (0.15 mL) or as a $H_2PtCl_6.6H_2O$ aqueous solution (0.4 mL, 1 mg mL^{-1}). The total volume of the 20 mM 4-MBA aqueous solution was maintained as 5 mL after Pt addition (i.e., the concentration of CTF-Bpy was maintained as 1 mg mL^{-1} in the solution). The H_2 evolution following Pt introduction was monitored by periodically sampling the reactor headspace and quantifying the evolved gases via gas chromatography (Shimadzu GC-2030). This gas chromatograph is equipped with a Barrier Discharge Ionization detector (BID) and Thermal conductivity detector (TCD) using argon as the carrier gas. Unless stated differently, evolution of gases in the headspace is measured in a closed system (i.e., batch measurements).

For the calculation of the turnover frequency (TOF), we assume all Pt (0.15 mg = 0.77 μmol) functions as the active sites for hydrogen evolution in the dark. Regarding the reaction time, we only count in the time after Pt been added over the ca. 3-h measurement (2.2 h).

2. Synthetic procedures

2.1 Synthesis of 2,2'-bipyridine-5,5'-dicarboximidamide dihydrochloride

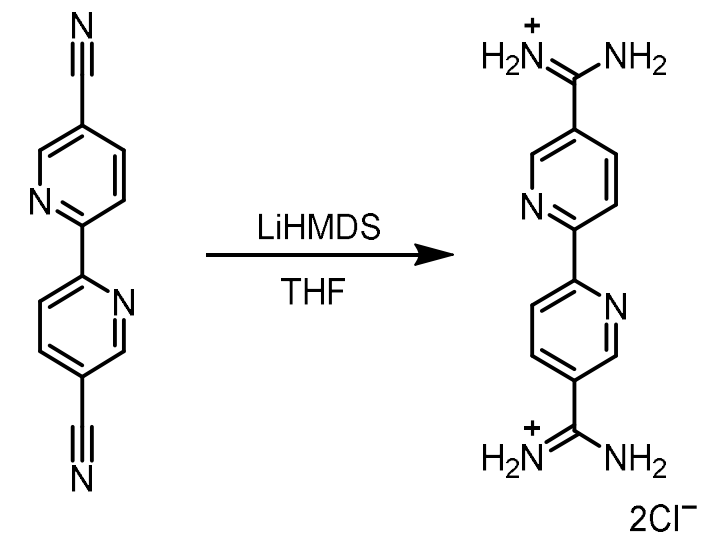

Scheme S1. Synthesis of 2,2'-bipyridine-5,5'-dicarboximidamide hydrochloride.[6]

Under argon atmosphere in a dried round bottom flask, at 0 °C, Lithium bis(trimethylsilyl)amide (LiHMDS) solution (13.6 mL, 1 M in THF) was added dropwise to a suspension of 2,2'-bipyridine-5,5'-dicarbonitrile (700 mg, 3.4 mmol) in 11 mL anhydrous THF. Upon complete addition, the ice bath was removed and the solution was then stirred at room temperature for 3 h. In a separate, dried flask under argon, acetyl chloride (2 mL, 27.6 mmol, 98%) was added to 13 mL anhydrous ethanol to create a ~ 2 M HCl solution in ethanol. Then, the reaction mixture was cooled to 0 °C, and the freshly prepared HCl solution in ethanol was carefully added. The mixture was stirred at room temperature overnight. Product was collected by filtration, washed thoroughly with THF, acetone and EtOH. The product was then refluxed briefly in a mixture of 50 mL EtOH + 3 mL water, hot filtrate. Repeat this reflux procedure once more for further purification. The product was dried at 60 °C in vacuum oven overnight to obtain pure 2,2'-bipyridine-5,5'-dicarboximidamide dihydrochloride as off-white powders in 56 % yield (590 mg).

^{1}H NMR (400 MHz, DMSO-d$_6$): δ = 9.76 (s, 4H), 9.47 (s, 4H), 9.17 (s, 2H), 8.64 (dd, *J* = 8.0 Hz, *J* = 4.0 Hz, 2H), 8.46 (d, *J* = 8.0 Hz, 2H) ppm. ^{13}C NMR (100 MHz, DMSO-d$_6$): δ = 163.72, 157.51, 149.08, 138.00, 125.39, 120.77 ppm. Anal. Cald for $C_6H_7N_3Cl$: C, 46.02; H, 4.51; N, 26.83. Found: C: 44.18; H: 4.43; N: 25.25.

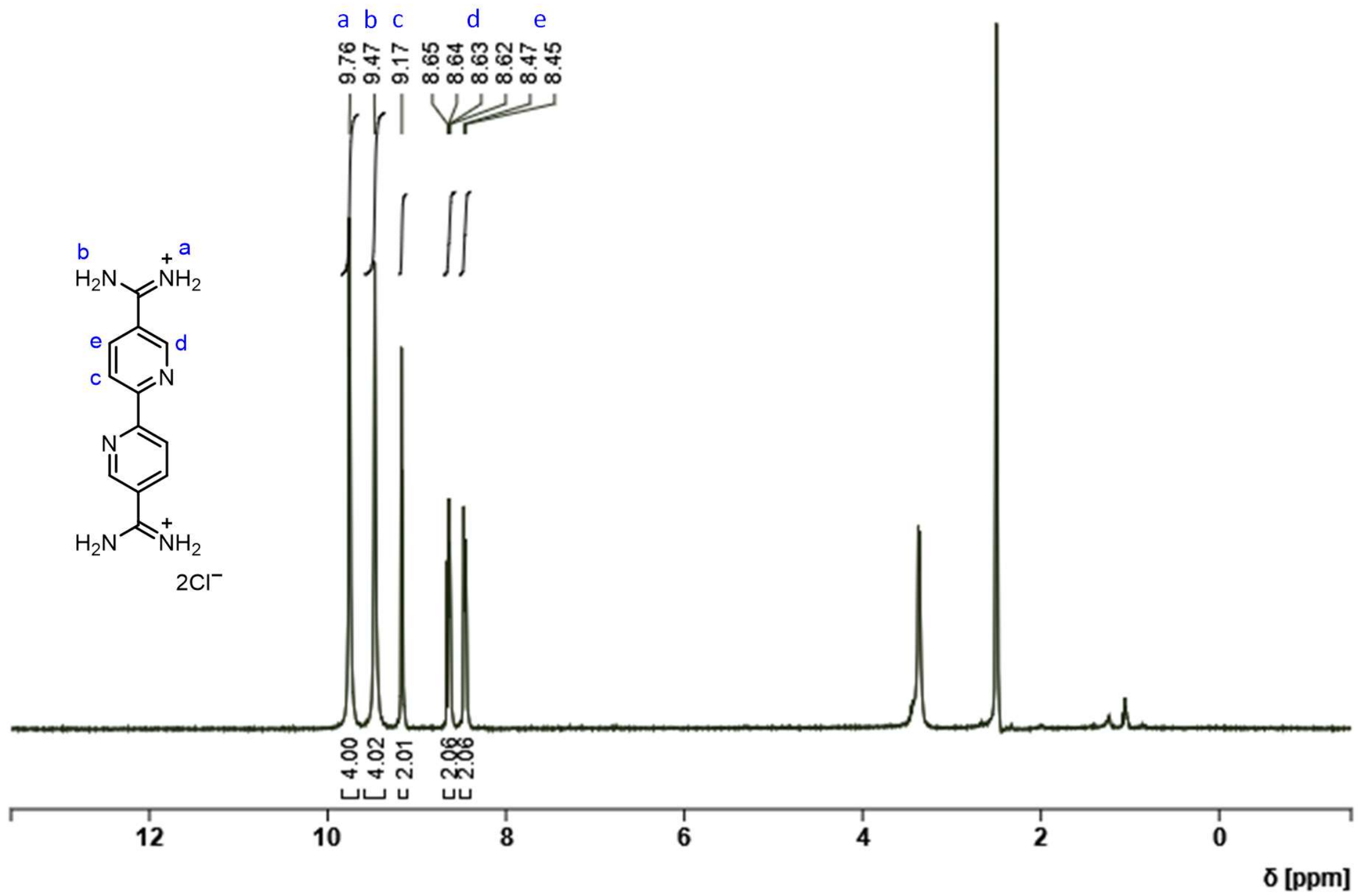

Figure S1. ^{1}H NMR spectrum of 2,2'-bipyridine-5,5'-dicarboximidamide dihydrochloride in DMSO-d_6.

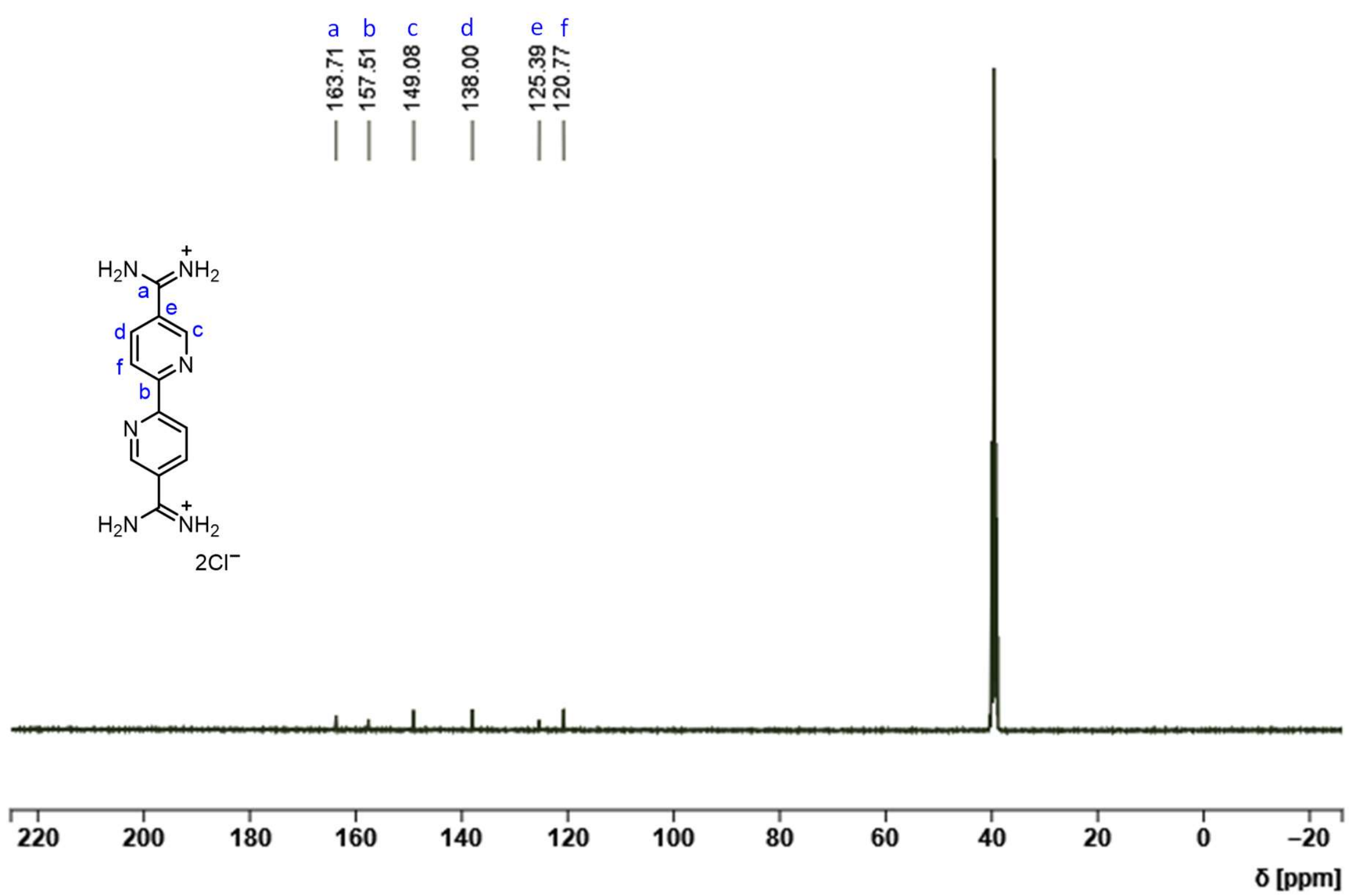

Figure S2. ^{13}C NMR spectrum of 2,2'-bipyridine-5,5'-dicarboximidamide dihydrochloride in DMSO-d_6.

2.2 Synthesis of CTFs

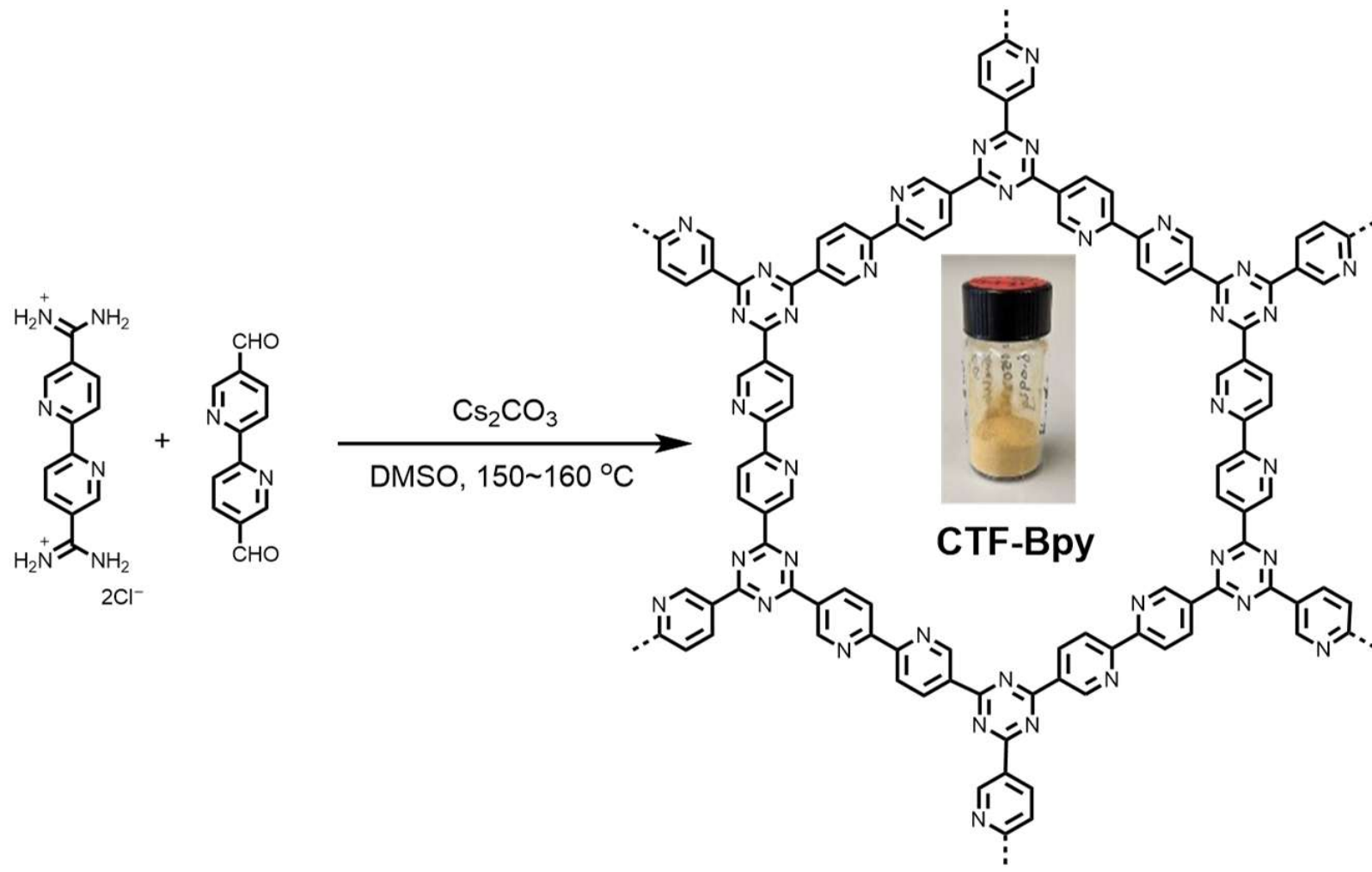

Scheme S2. Synthesis of CTF-Bpy.[6]

Synthesis of CTF-Bpy: The synthesis of CTF-Bpy was conducted following a previously published literature procedure.[6] Specifically, a flask was charged with 2,2'-bipyridine-5,5'-dicarboximidamide dihydrochloride (312 mg, 1.0 mmol), Cs_2CO_3 (490 mg, 1.5 mmol), 1, 2-dichlorobenzene (10 mL), DMSO (10 mL) and this mixture was heated to 100 °C with constant stirring. In a separate flask, 2, 2'-bipyridyl-5, 5'-dialdehyde (106 mg, 0.5 mmol) was dissolved in 1, 2-dichlorobenzene (10 mL) and DMSO (10 mL). Then, the solution of 2,2'-bipyridine-5,5'-dicarbaldehyde was added into 2,2'-bipyridine-5,5'-dicarboximidamide dihydrochloride solution by a syringe pump with a rate of 20 μL min^{-1}. Upon complete addition, the mixture was heated at 100 °C for another 24 h. The flask was then fitted with a water-cooled condenser and the reaction mixture was heated to 180 °C and kept at this temperature for 48 h. The precipitate from the yellow suspension was washed sequentially with DMF (3 × 100 mL), then with deionized water (3 × 100 mL). The filtered solid was freeze-dried for 48 h yielding CTF-Bpy as bright yellow powder (250 mg, yield 81 %). Anal. Calcd for $(C_6H_3N_2{\cdot}H_2O)_n$: C, 59.50; H, 4.16; N, 23.13; O, 13.21. Found: C, 59.95; H, 3.88; N, 22.84. Test service from Pascher shows a Cs content of 0.01 wt.%.

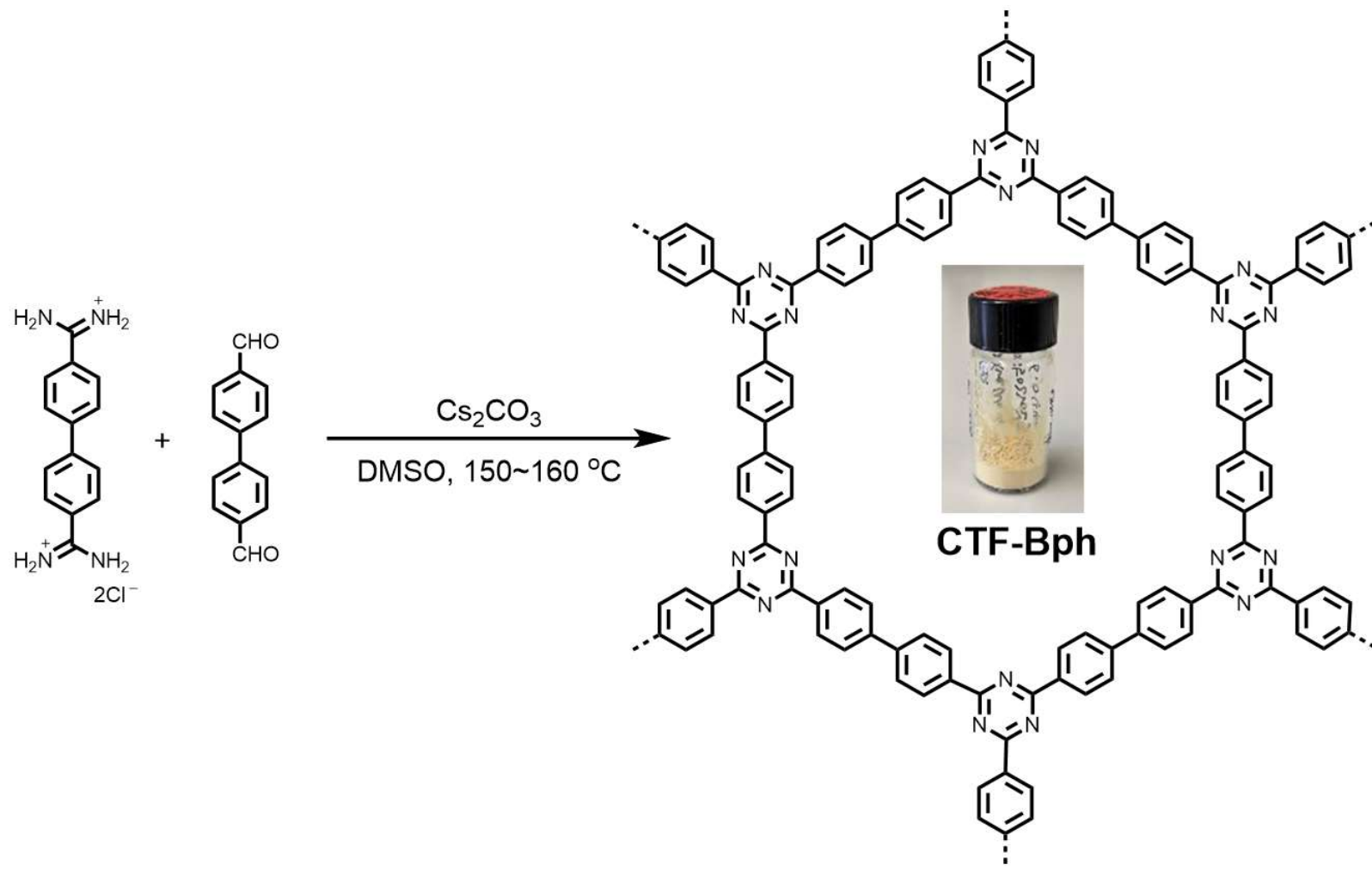

Scheme S3. Synthesis of CTF-Bph.[7]

Synthesis of CTF-Bph: The synthesis of CTF-Bph was conducted following a previously published literature procedure.[7] Specifically, a flask was charged with 1,1'-biphenyl-4,4'-bis(carboximidamide) dihydrochloride (311 mg, 1.0 mmol), Cs_2CO_3 (490 mg, 1.5 mmol), DMSO (20 mL), and this mixture was heated at 100 °C with constant stirring for 30 min. In a separate flask, 1,1'-biphenyl-4,4'-dicarbaldehyde (106 mg, 0.5 mmol) was dissolved in 20 mL DMSO, and stirred at 50 °C for 30 min. Then, the solution of 1,1'-biphenyl-4,4'-dicarbaldehyde was added into 1,1'-biphenyl-4,4'-bis(carboximidamide) dihydrochloride solution by a syringe pump with a rate of 30 μL min^{-1} at 100 °C over 12 h and the reaction mixture was kept at 100 °C for total 24 h. The flask was fitted with a water-cooled condenser and the reaction mixture was heated at 150 °C for 12 h, then at 180 °C for 48 h. The precipitate from the yellow suspension was washed sequentially with DMF (3 × 100 mL), then deionized water (3 × 100 mL). The filtered solid was freeze-dried for 48 h yielding CTF-Bph as pale-yellow powder (200 mg, yield 65 %). Anal. Calcd for $(C_7H_4N)_n$: C, 82.33; H, 3.95; N, 13.72. Found: C, 76.91; H, 4.41; N, 12.43. Test service from Pascher shows a Cs content of 0.001 wt.%.

3. Characterization of CTF-Bpy

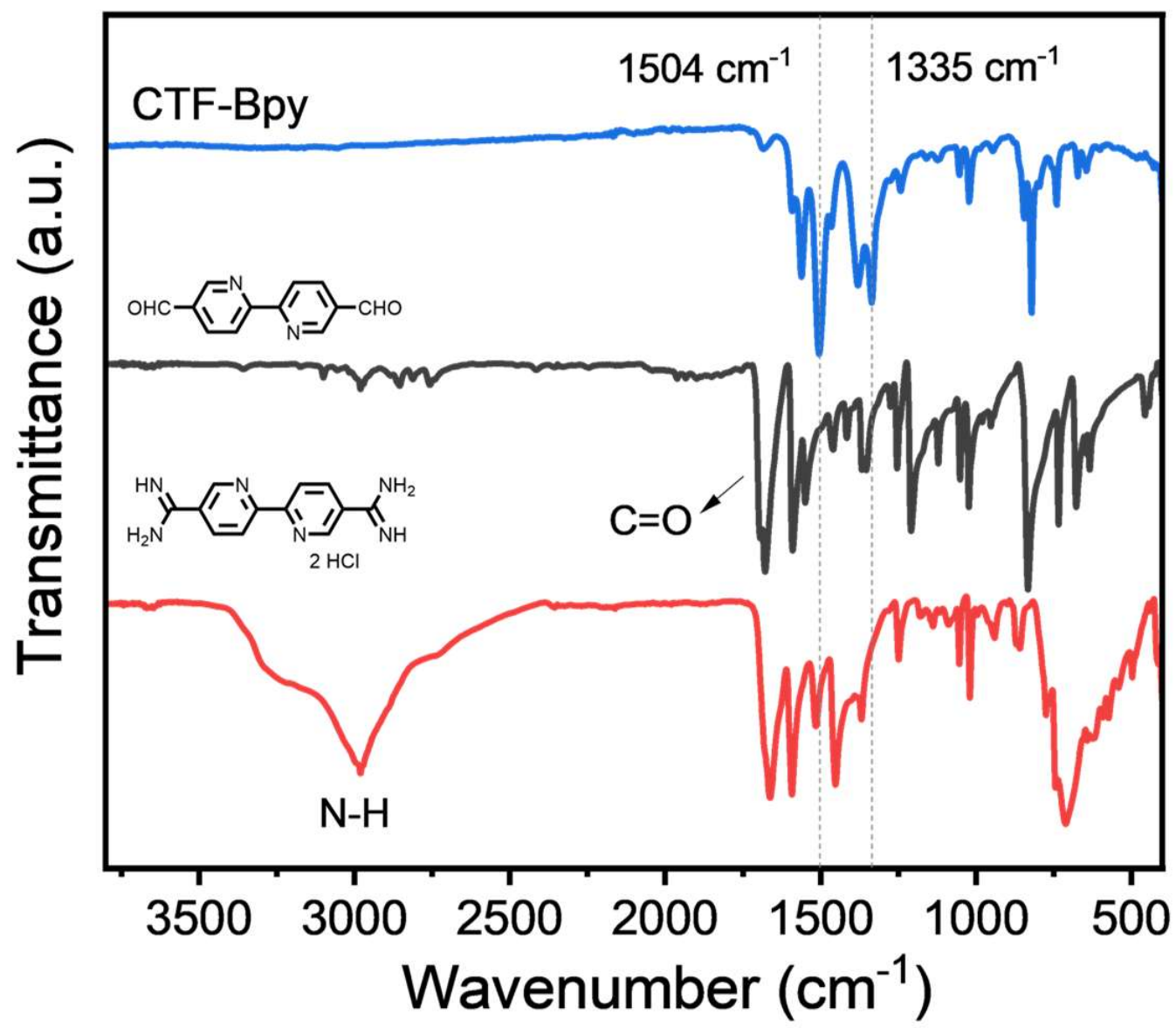

Figure S3. FTIR spectra of CTF-Bpy and the precursors. The characteristic vibrations of triazine unit at 1504 and 1335 cm^{-1} are indicated.

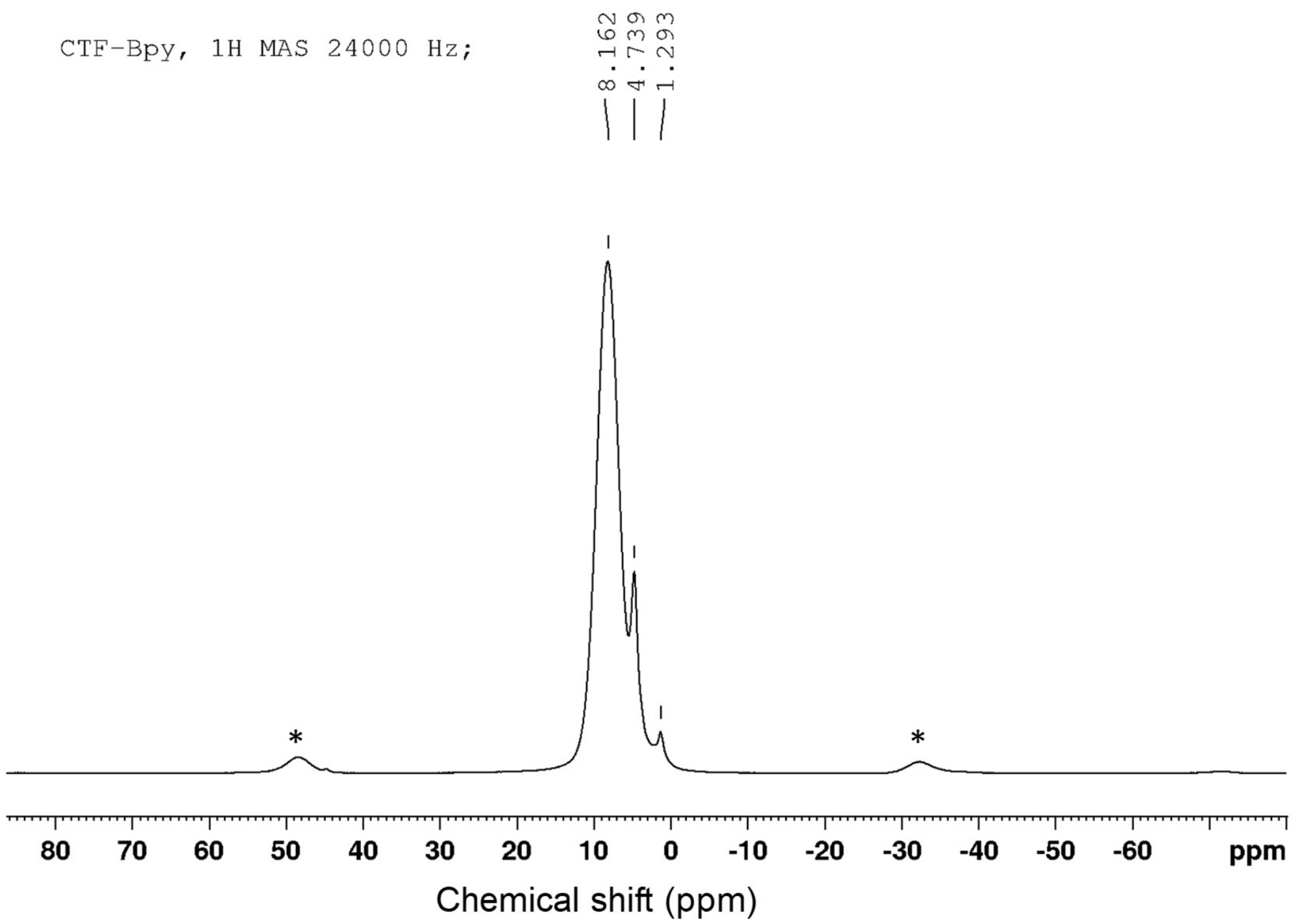

Figure S4. Solid-state ^{1}H MAS NMR spectrum of CTF-Bpy. Signal at 8.162 ppm corresponds to bipyridine protons. Signals at 4.739 and 1.293 ppm can be ascribed to water and organic solvent residues, respectively. Asterisks denote spinning sidebands.

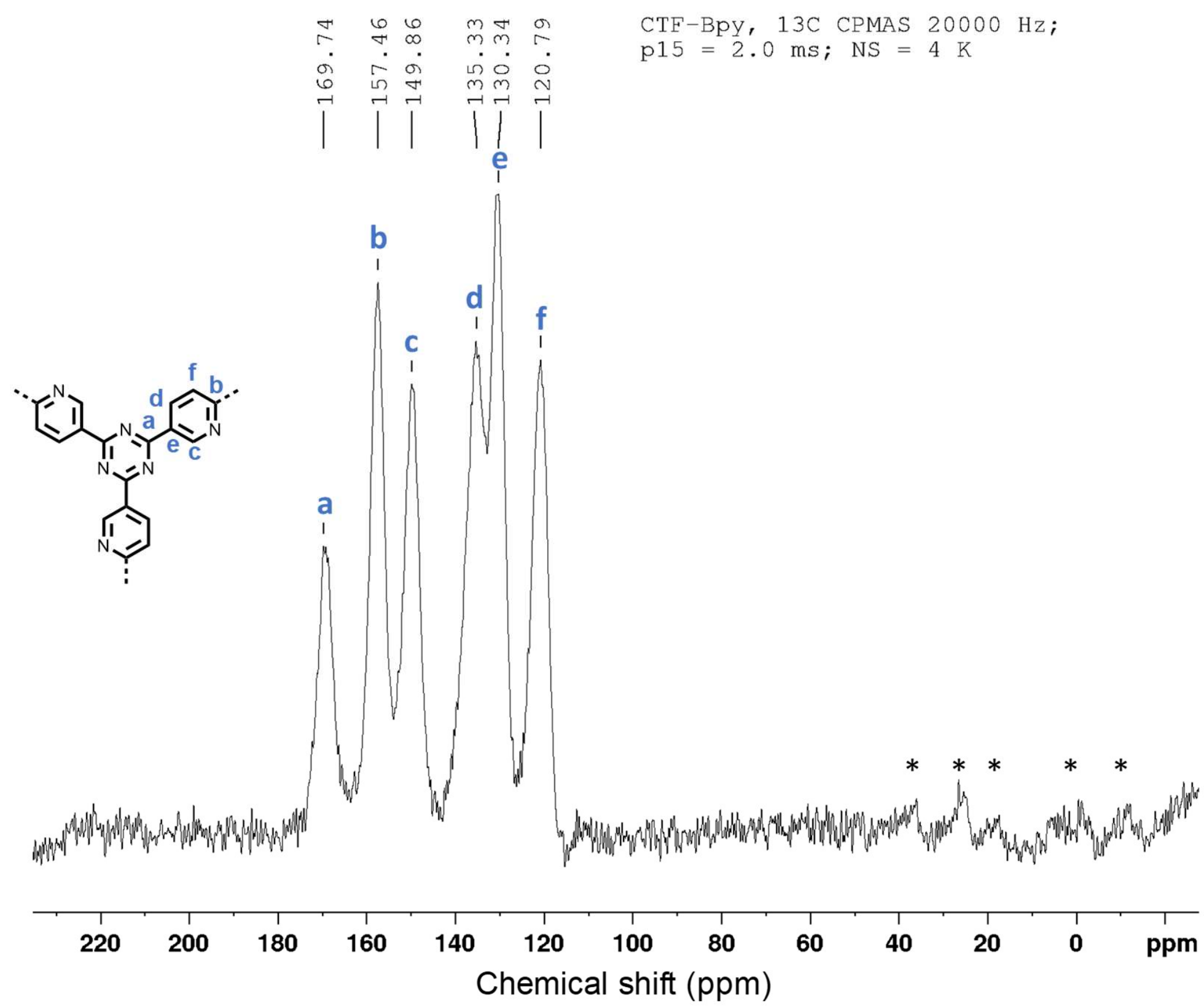

Figure S5. Solid-state ^{13}C CP/MAS NMR spectrum of CTF-Bpy with assigned signals in the molecular fragment. Asterisks denote spinning sidebands.

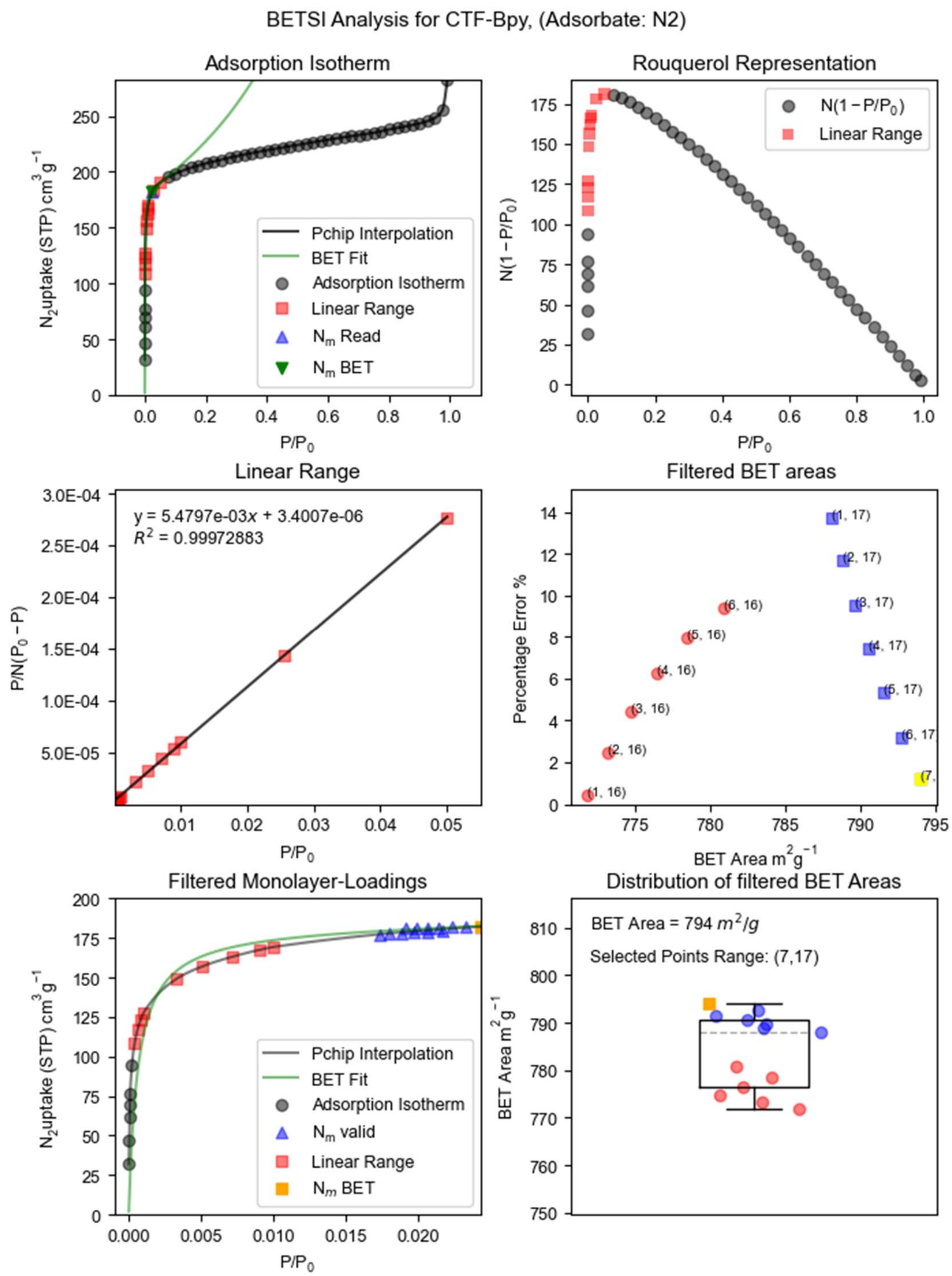

Figure S6. N_2 sorption isotherm analysis of CTF-Bpy using the BETSI software.[1]

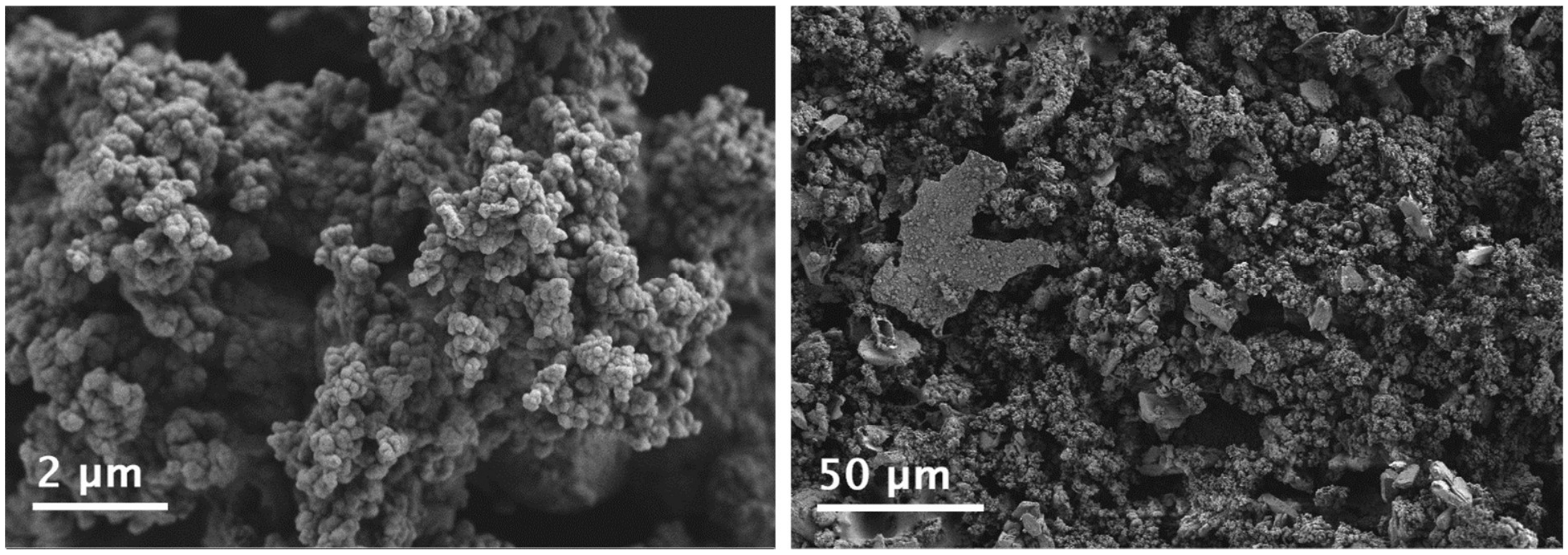

Figure S7. SEM images of CTF-Bpy at different magnifications.

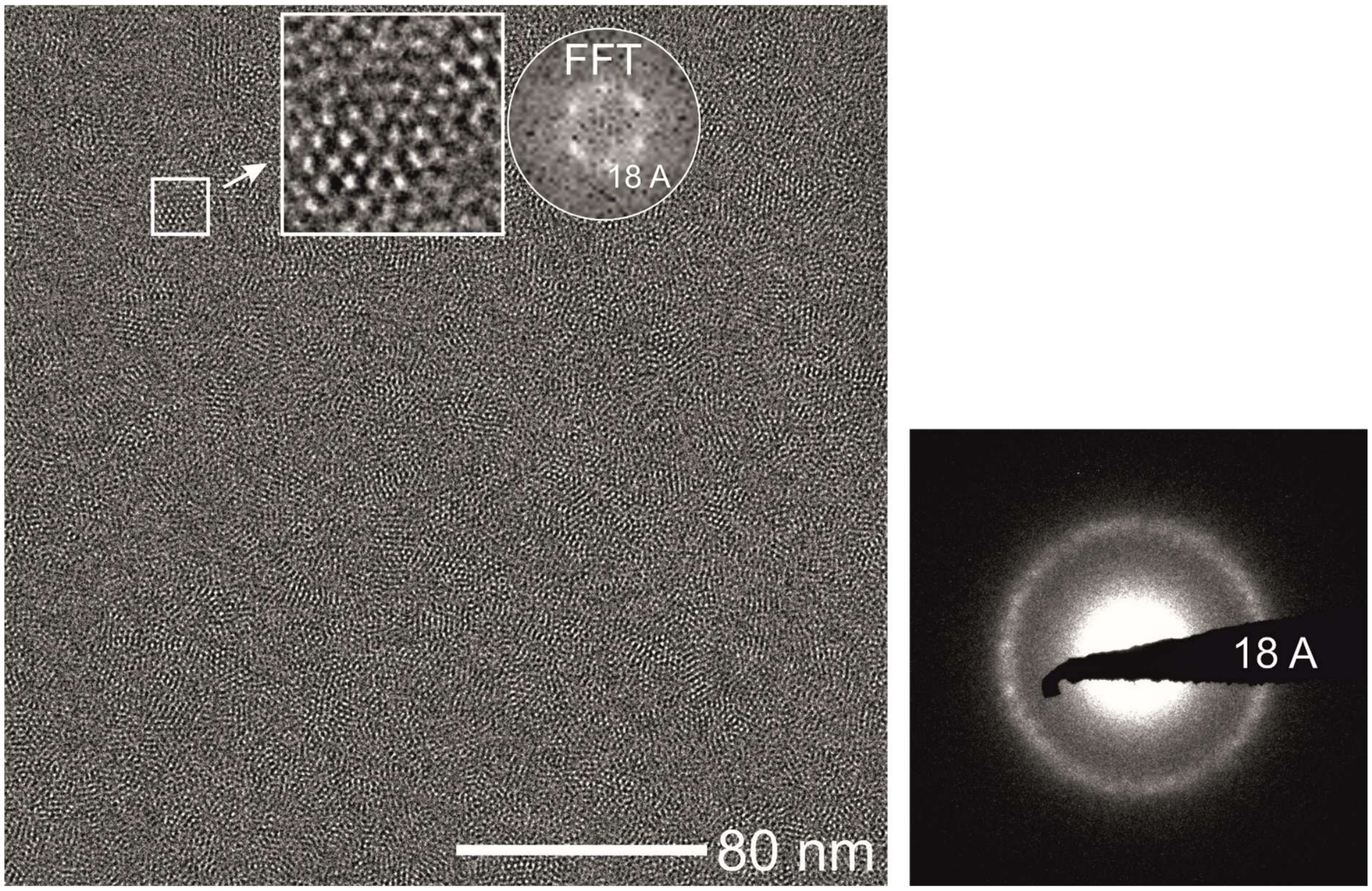

Figure S8. HR-TEM image (left) and the selected area electron diffraction (SAED, right) of CTF-Bpy. Inset of the image on the left is the enlarged image of the selected area showing the pores and the corresponding FFT (Fast Fourier Transform) of the selected area.

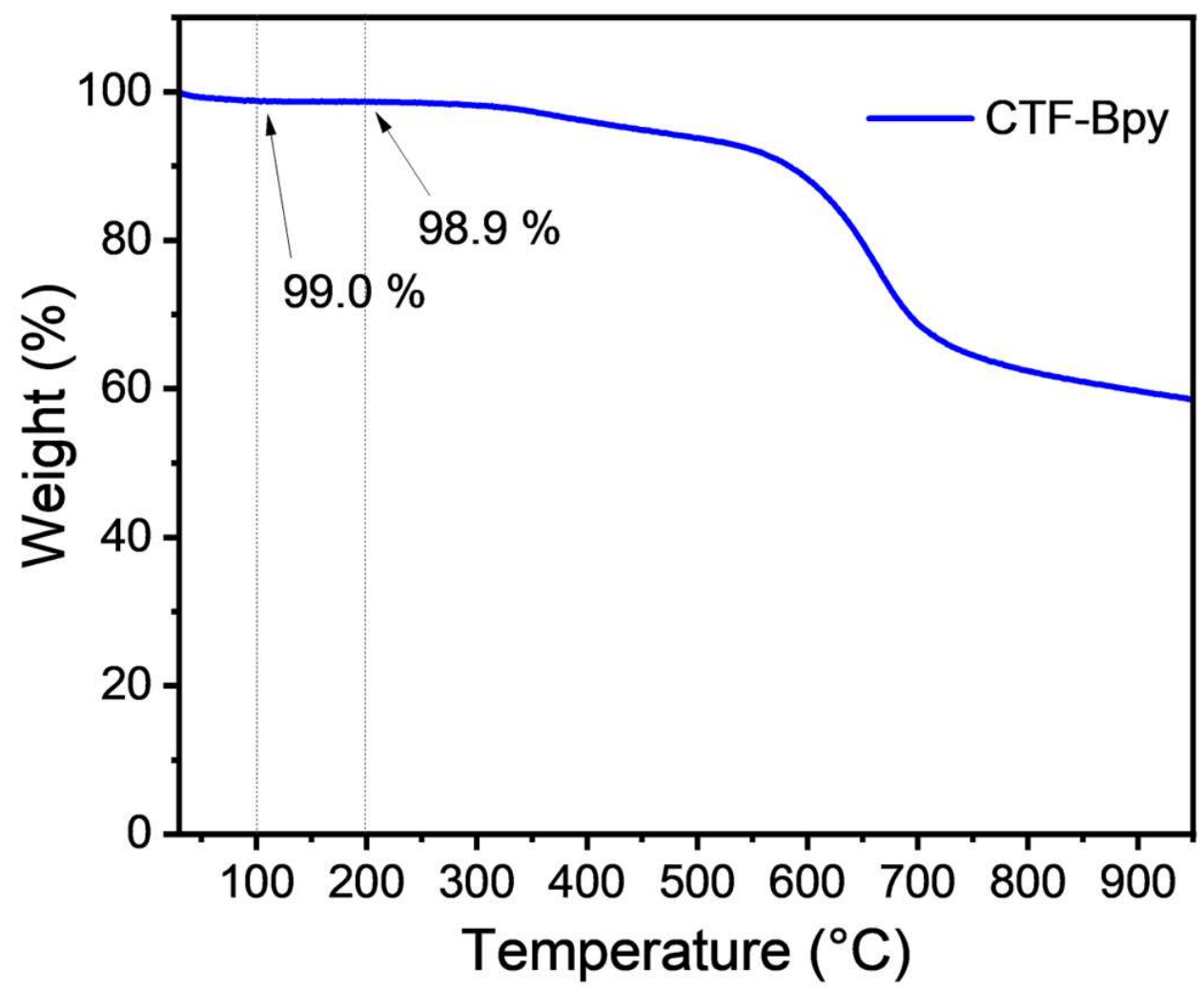

Figure S9. TGA curve of CTF-Bpy under Ar atmosphere.

4. Reversible light-induced radical formation

4.1 Band gap of CTF-Bpy

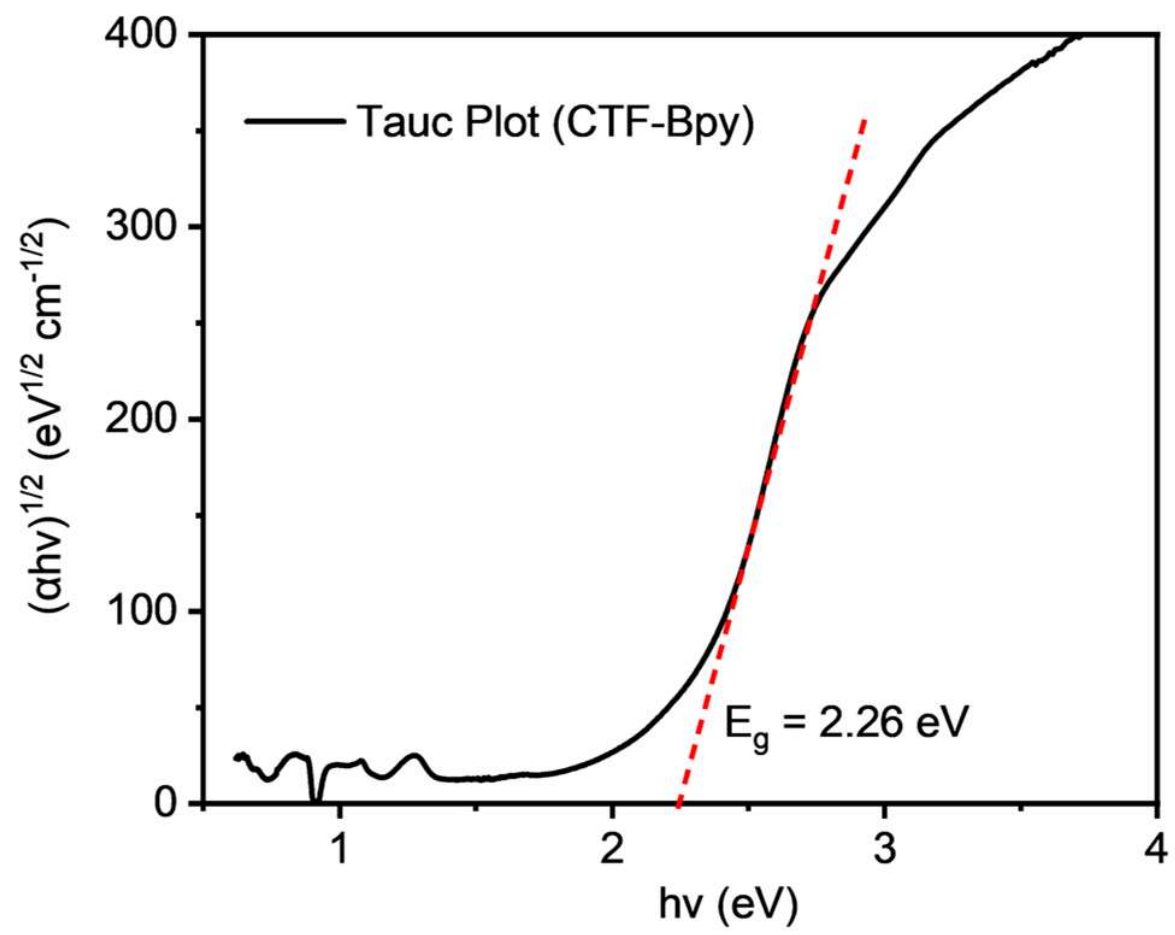

Figure S10. The Tauc plot of CTF-Bpy obtained from UV-vis-NIR spectrum and extracted optical band gap.

4.2 UV-vis-NIR spectra

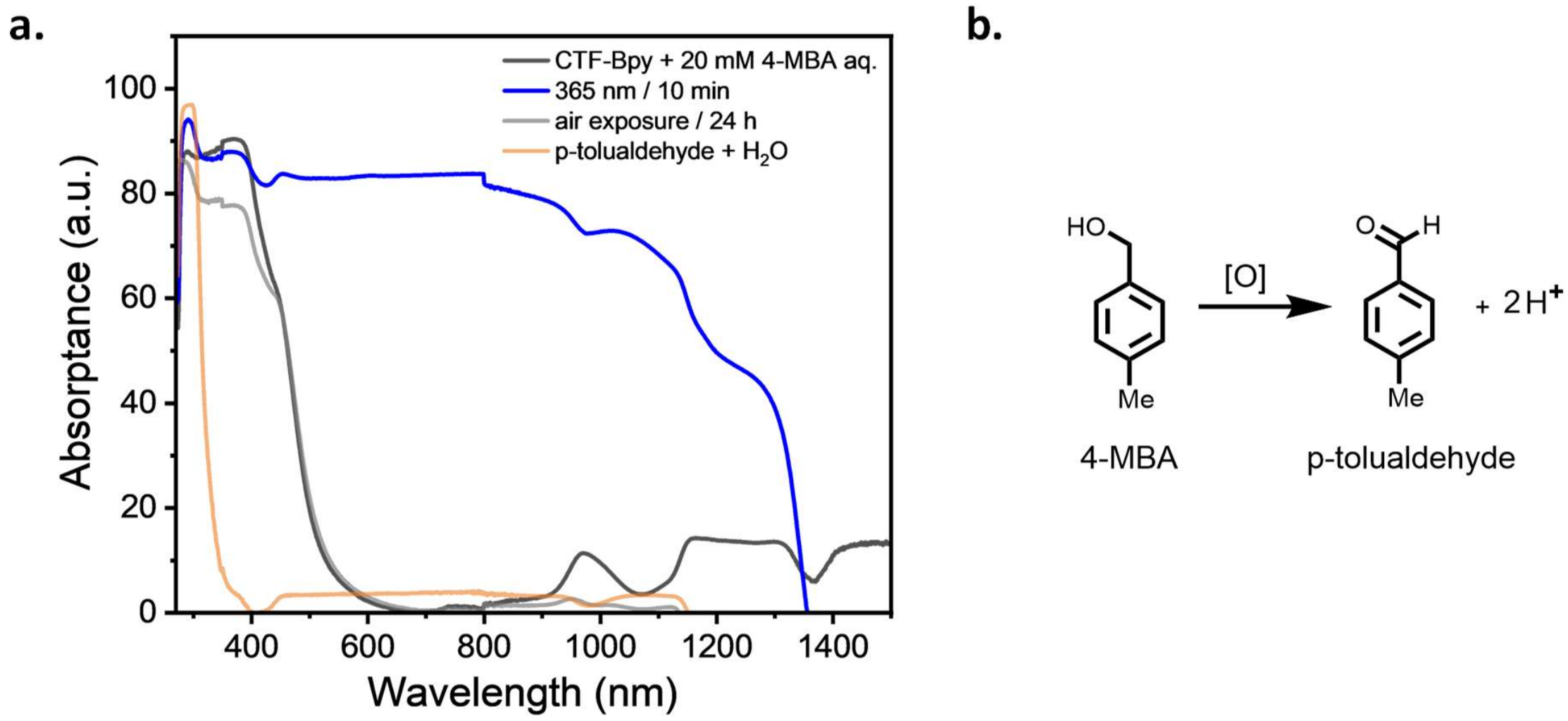

Figure S11. (a) UV-vis-NIR absorptance spectra of CTF-Bpy aqueous suspension in the presence of 20 mM 4-MBA before and after 10 min UV illumination, after exposing the photocharged suspension to air for 24 h, and the spectrum of *p*-tolualdehyde dispersion in H_2O. (b) Schematic representation of the oxidation of 4-MBA to *p*-tolualdehyde.

The intensity mismatch between the spectrum after 24 h air exposure and the spectrum upon illumination may arise from differences in baseline correction. Although the radical signal between 430-1370 nm was quenched after 5 min of air exposure, but the original spectrum was not fully restored, hence we collected the spectrum after 24 h. See below **Figure S12** for more details.

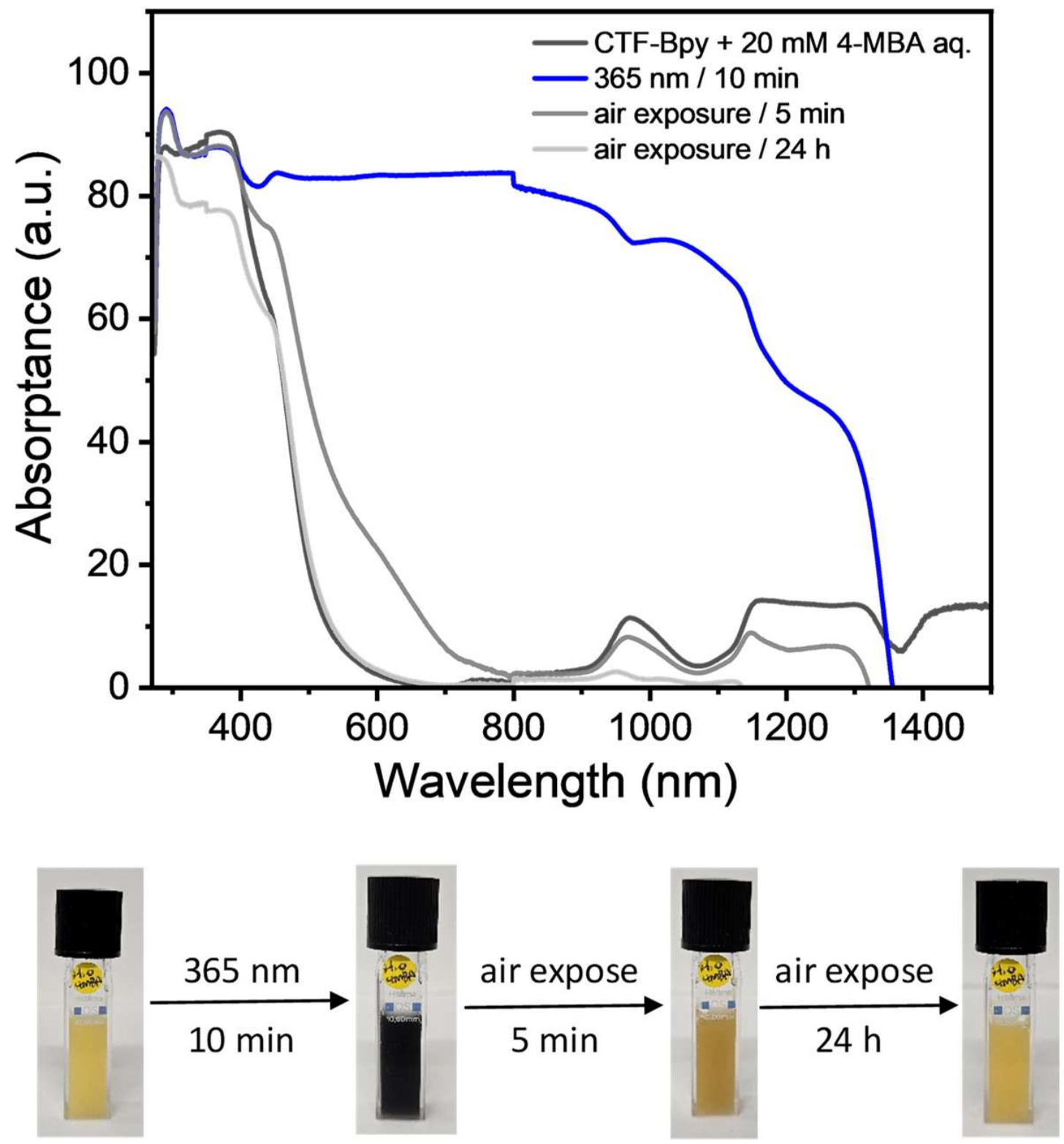

Figure S12. UV-vis-NIR absorptance spectra of CTF-Bpy in 20 mM 4-MBA aqueous suspension before and after 10 min illumination, and after exposing the photocharged suspension to air for 5 min, or 24 h. The photographs of corresponding suspensions are shown at the bottom.

a.

No.	1	2	3
sample	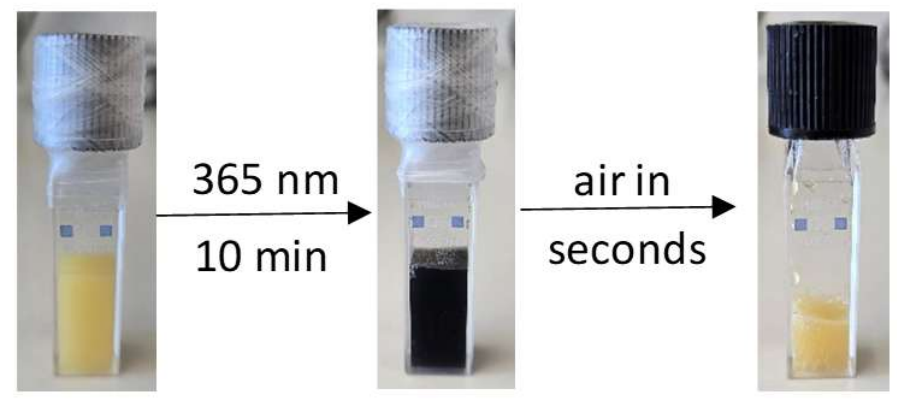		
H_2O_2 / µmol g^{-1}	112.66	66.65 (GB)*	823.72

b.

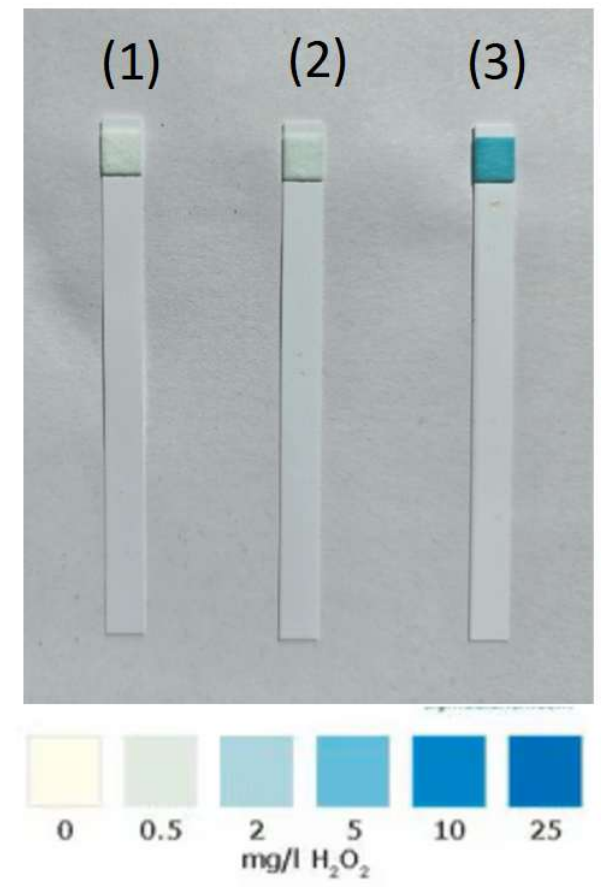

Figure S13. (a) Comparison of the amount of H_2O_2 in the suspension of CTF-Bpy in presence of 20 mM 4-MBA before and after 10 min illumination, and upon air exposure. The quantification of H_2O_2 was done through a spectrophotometric method based on the triiodide procedure.[4, 5] (b) Complementary H_2O_2 quantification in each suspension was done using test strips. *GB: measurements done in a glovebox. Under illumination, the H_2O_2 content decreased from 112.66 to 66.65 µmol g^{-1} due to the photodecomposition of H_2O_2.

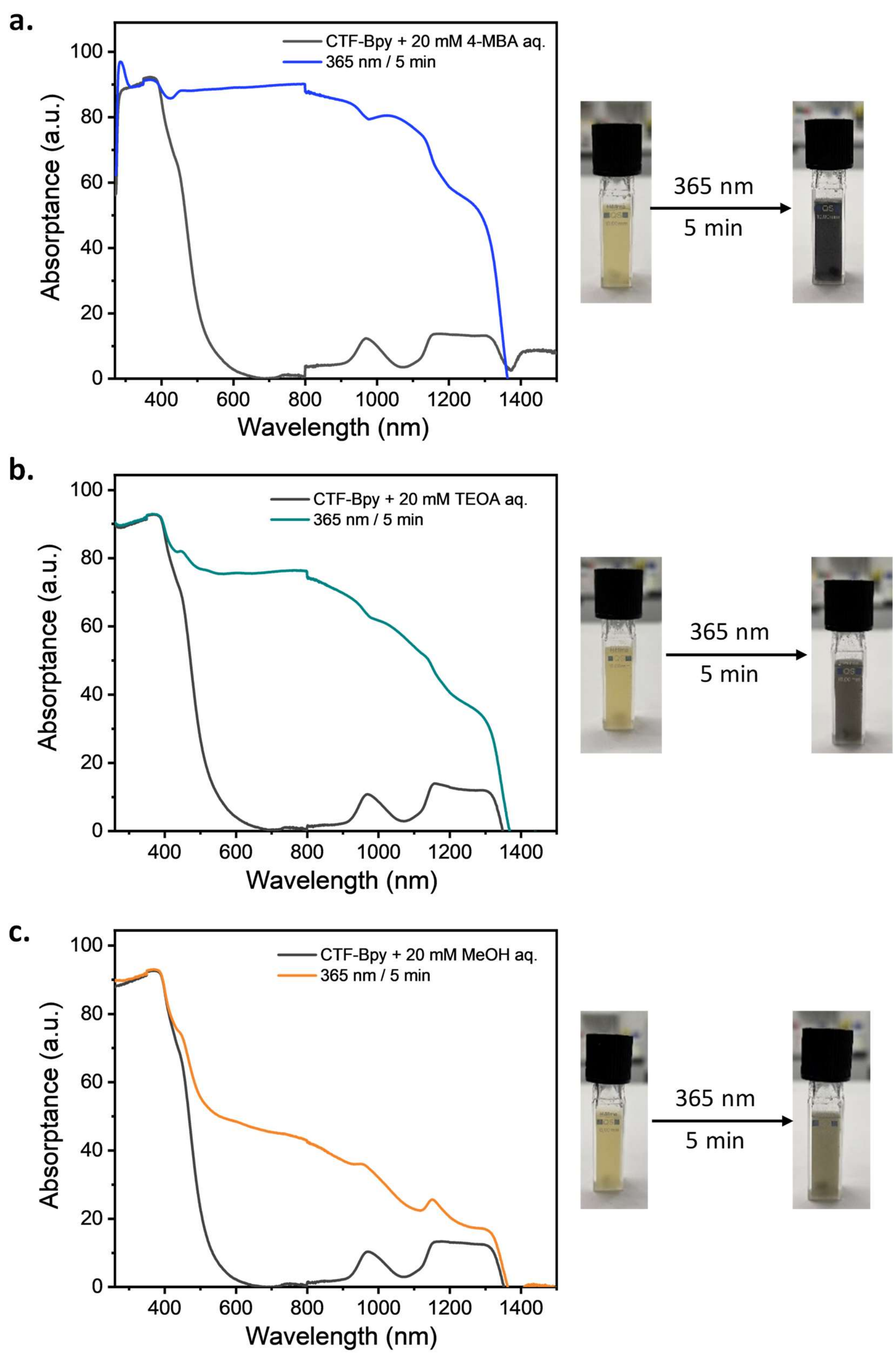

Figure S14. UV-vis-NIR absorptance spectra of CTF-Bpy aqueous suspension in the presence of 20 mM (a) 4-MBA, (b) TEOA and (c) MeOH before and after 5 min illumination. Corresponding photographs of the suspension before and after illumination are shown on the right.

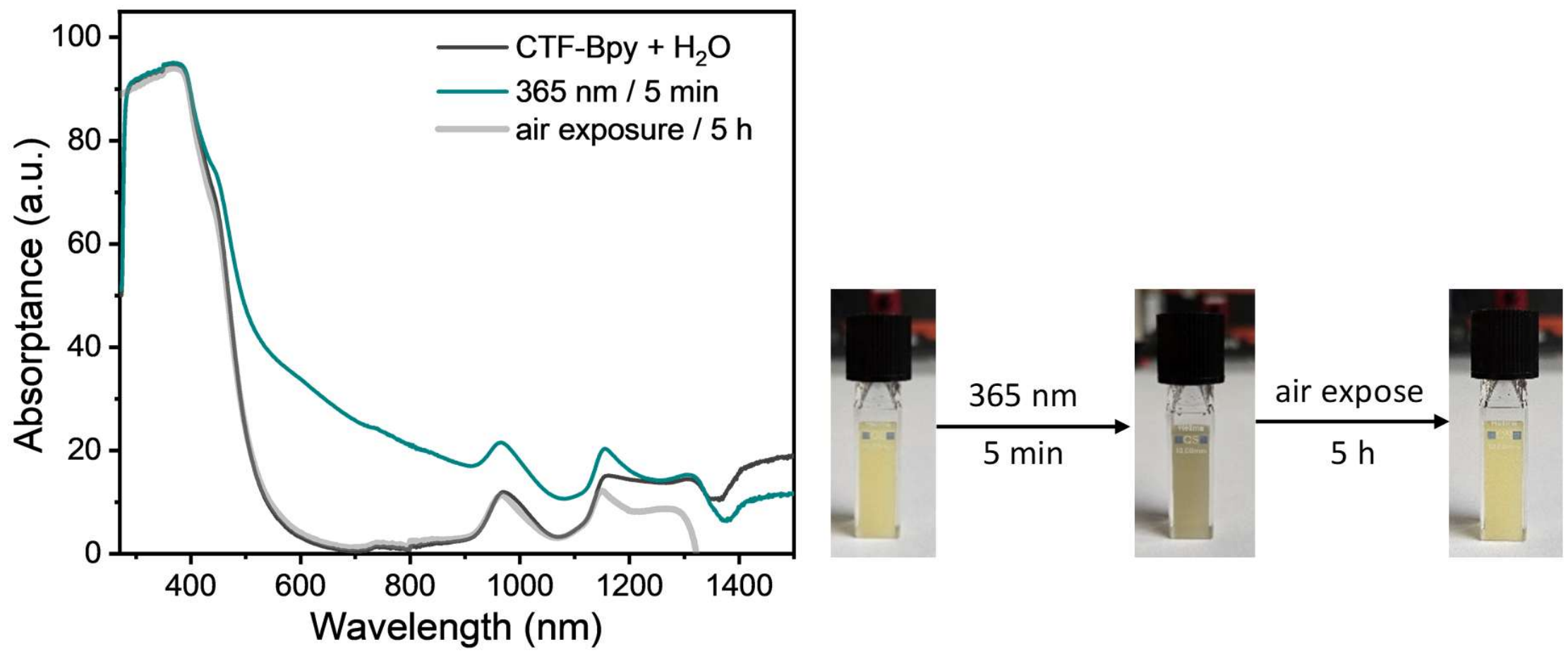

Figure S15. UV-vis-NIR absorptance spectra of CTF-Bpy aqueous suspension before and after illumination, and after 5 h exposure to air. On the right is the photograph of the three suspensions corresponding to the spectrum. This result suggests weak electron donating ability of H_2O for radical formation in CTF-Bpy under illumination.

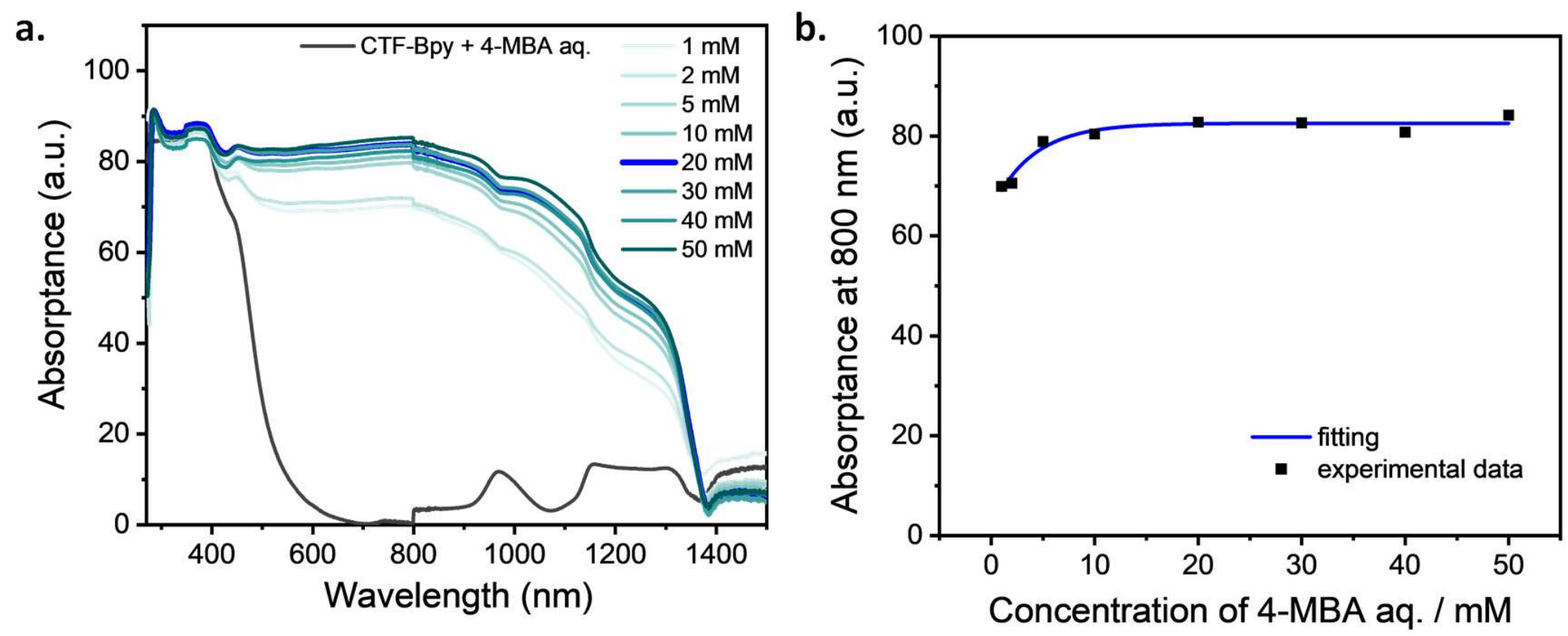

Figure S16. (a) UV-vis-NIR absorptance spectra of CTF-Bpy aqueous suspension with different concentrations of 4-MBA, before and after 1 min 365 nm illumination. (b) Absorptance at 800 nm as a function of 4-MBA concentration. Curve fitting suggests an optimal 4-MBA concentration of 20 mM for the light-induced radical formation in an aqueous CTF-Bpy suspension.

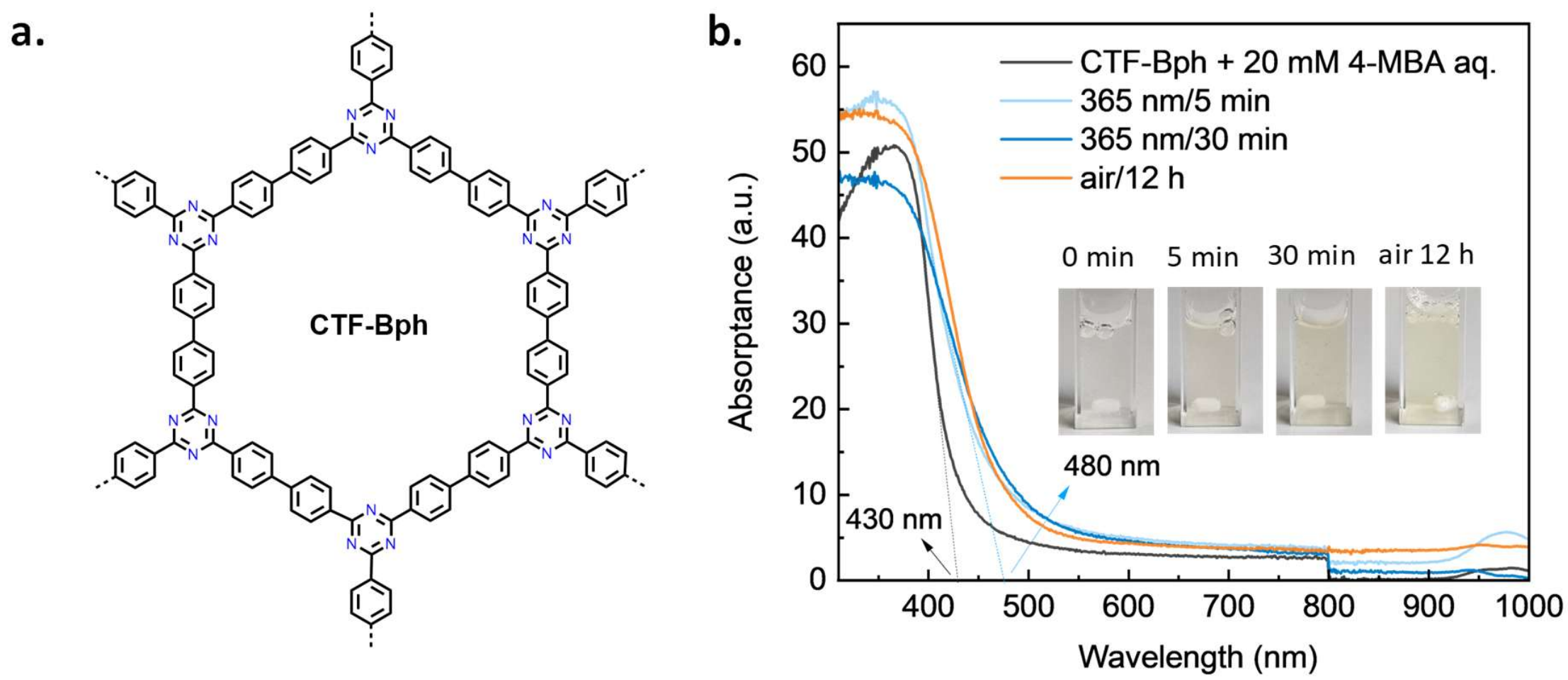

Figure S17. (a) Chemical structure of CTF-Bph. (b) UV-vis-NIR absorptance spectra of CTF-Bph aqueous suspension (1 mg/3 mL) in the presence of 20 mM 4-MBA before irradiation, after 5 and 30 min of irradiation by 365 nm UV light, and after 12 h of air exposure. Inset (b) shows photographs of the suspension before irradiation, after irradiation and after air exposure.

4.3 Characterization of CTF-Bph

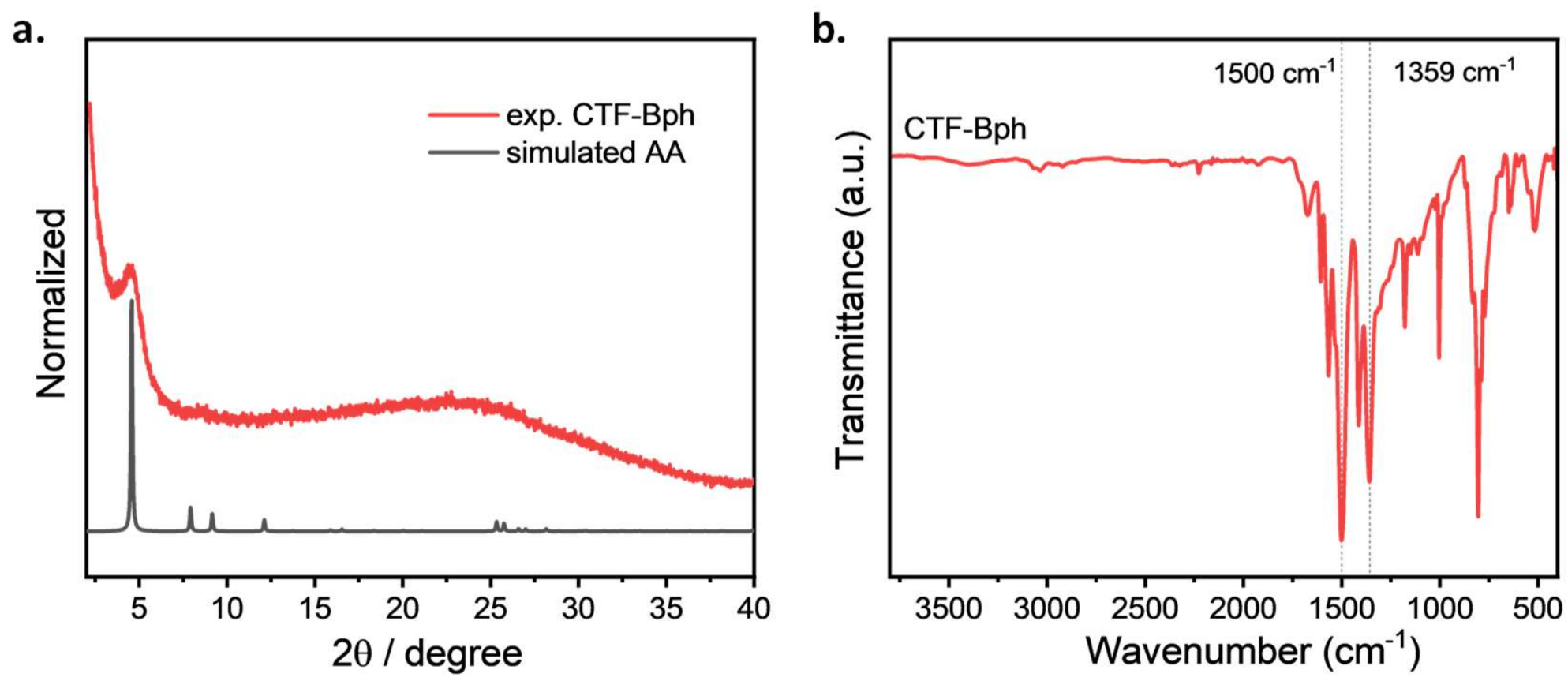

Figure S18. (a) Comparison of experimental and simulated PXRD patterns CTF-Bph. The experimental PXRD pattern matches the reported pattern in AA stacking mode.[7] (b) FTIR spectra of CTF-Bph. Characteristic vibrations of triazine unit at 1500 and 1359 cm^{-1} are indicated.

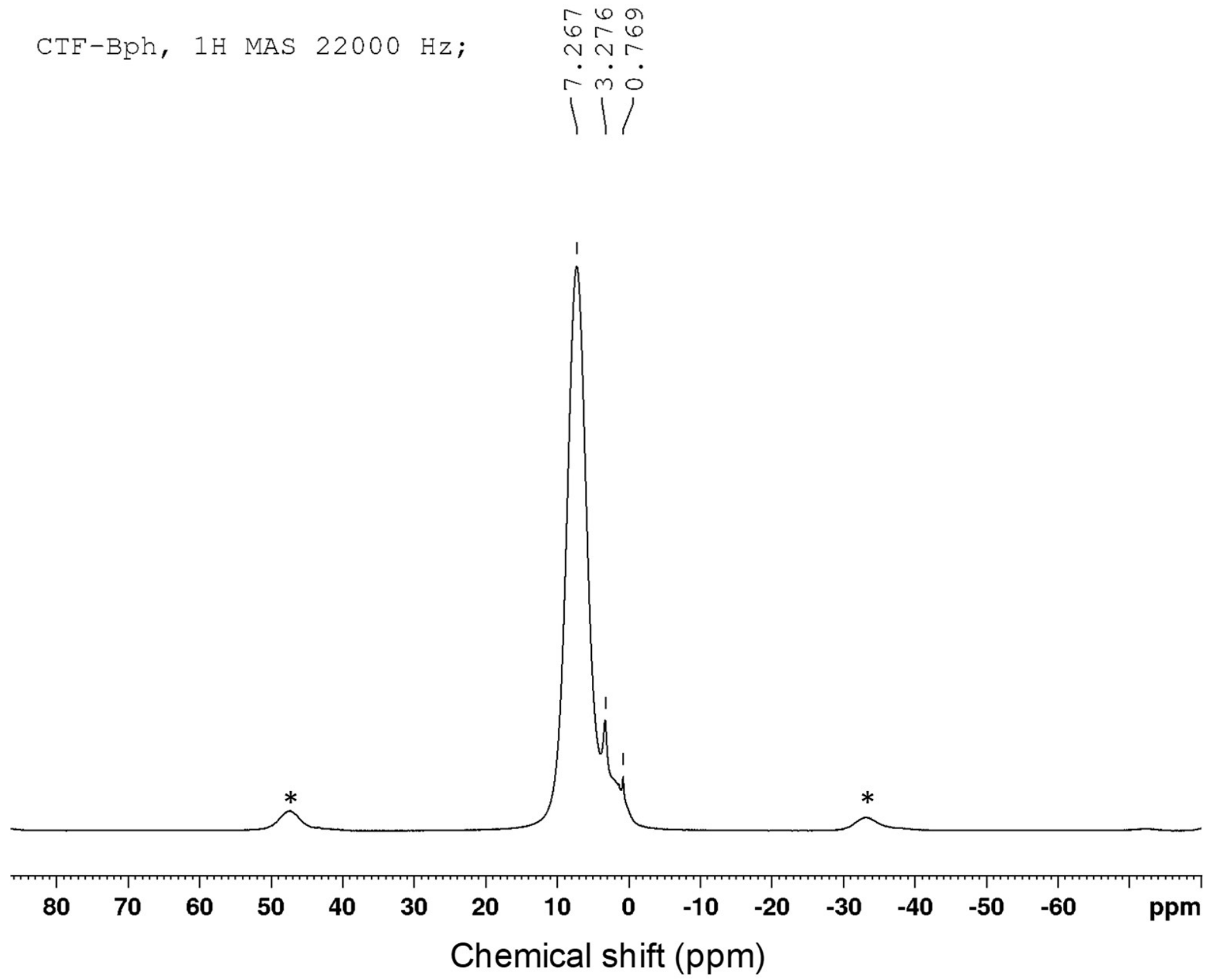

Figure S19. Solid-state ^{1}H MAS NMR spectrum of CTF-Bph. Signal at 7.267 ppm corresponds to aromatic protons of biphenyl unit. Peaks at 3.276 and 0.769 ppm correspond to water and residual organic solvent, respectively. Asterisks denote spinning sidebands.

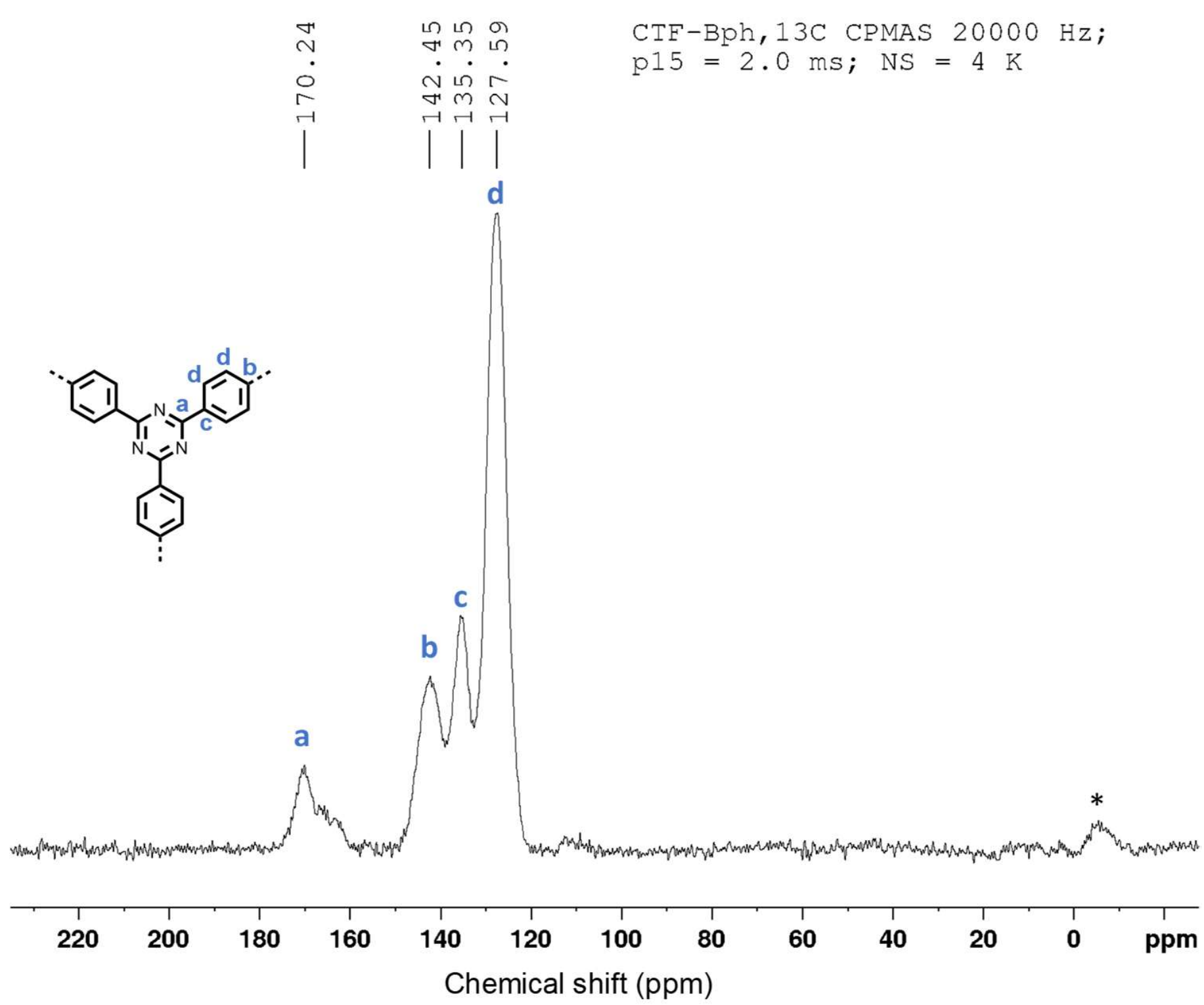

Figure S20. Solid-state ^{13}C CP/MAS NMR spectrum of CTF-Bph with assigned signals in the molecular fragment. Asterisks denote spinning sidebands.

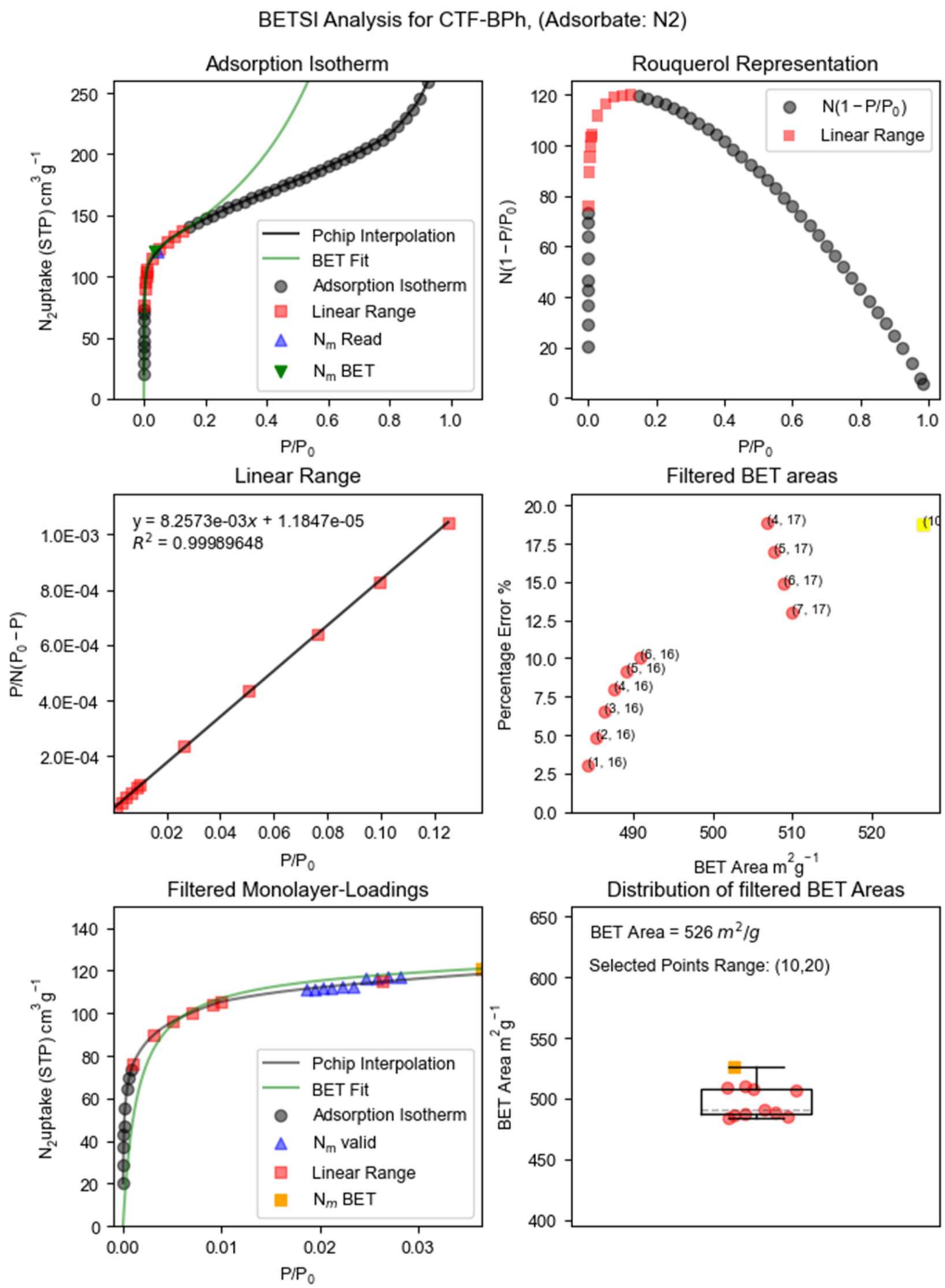

Figure S21. N_2 sorption isotherm analysis of CTF-Bph using BETSI software.[1]

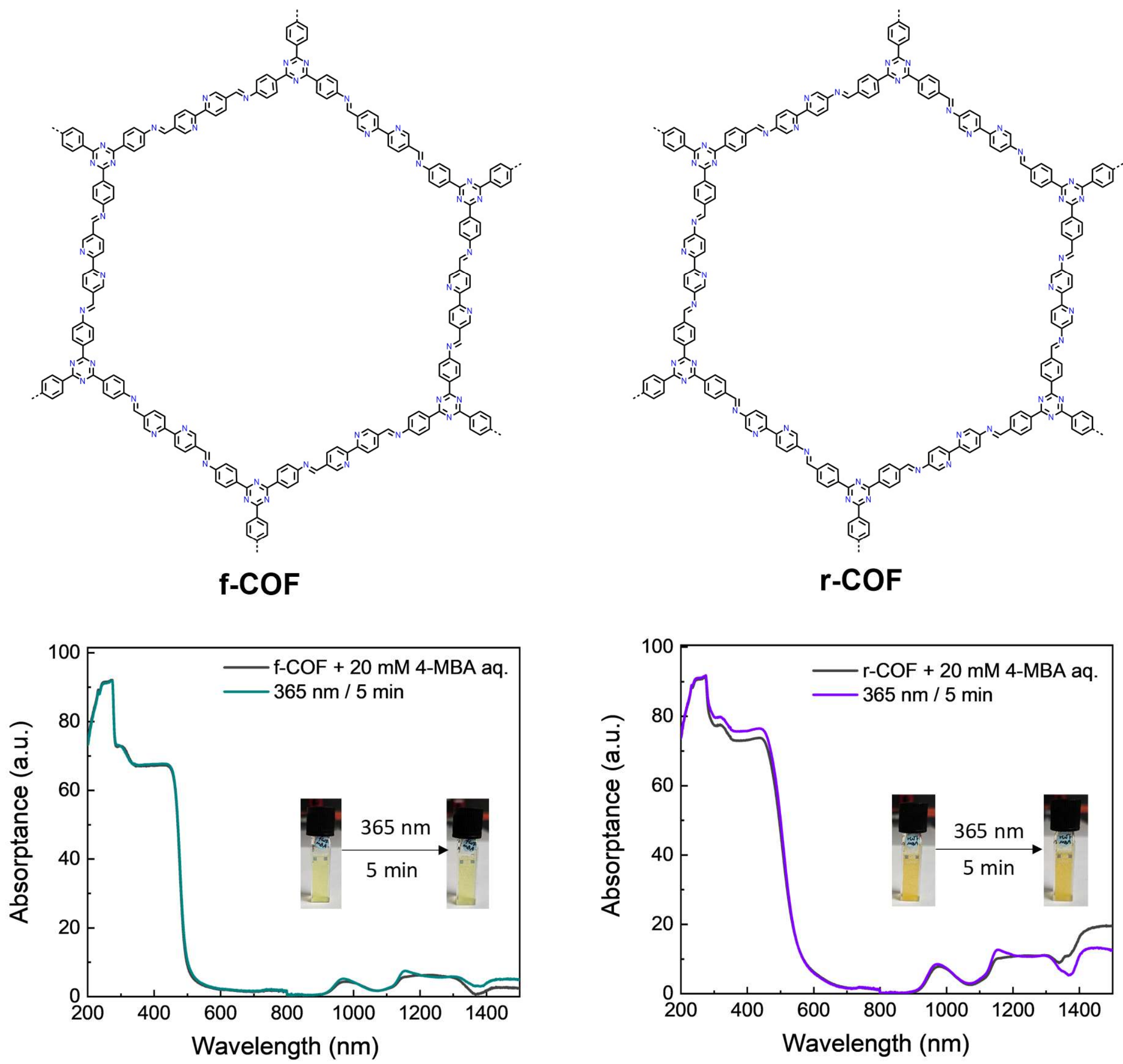

Figure S22. Chemical structure of the 2D f-COF and r-COF, and their corresponding UV-vis-NIR absorptance spectra in 20 mM 4-MBA aqueous solution (1 mg/mL) before and after 5 min of 365 nm UV irradiation. Insets show photographs of the suspensions before and after illumination.[8]

4.4 Cyclic voltammetry measurements

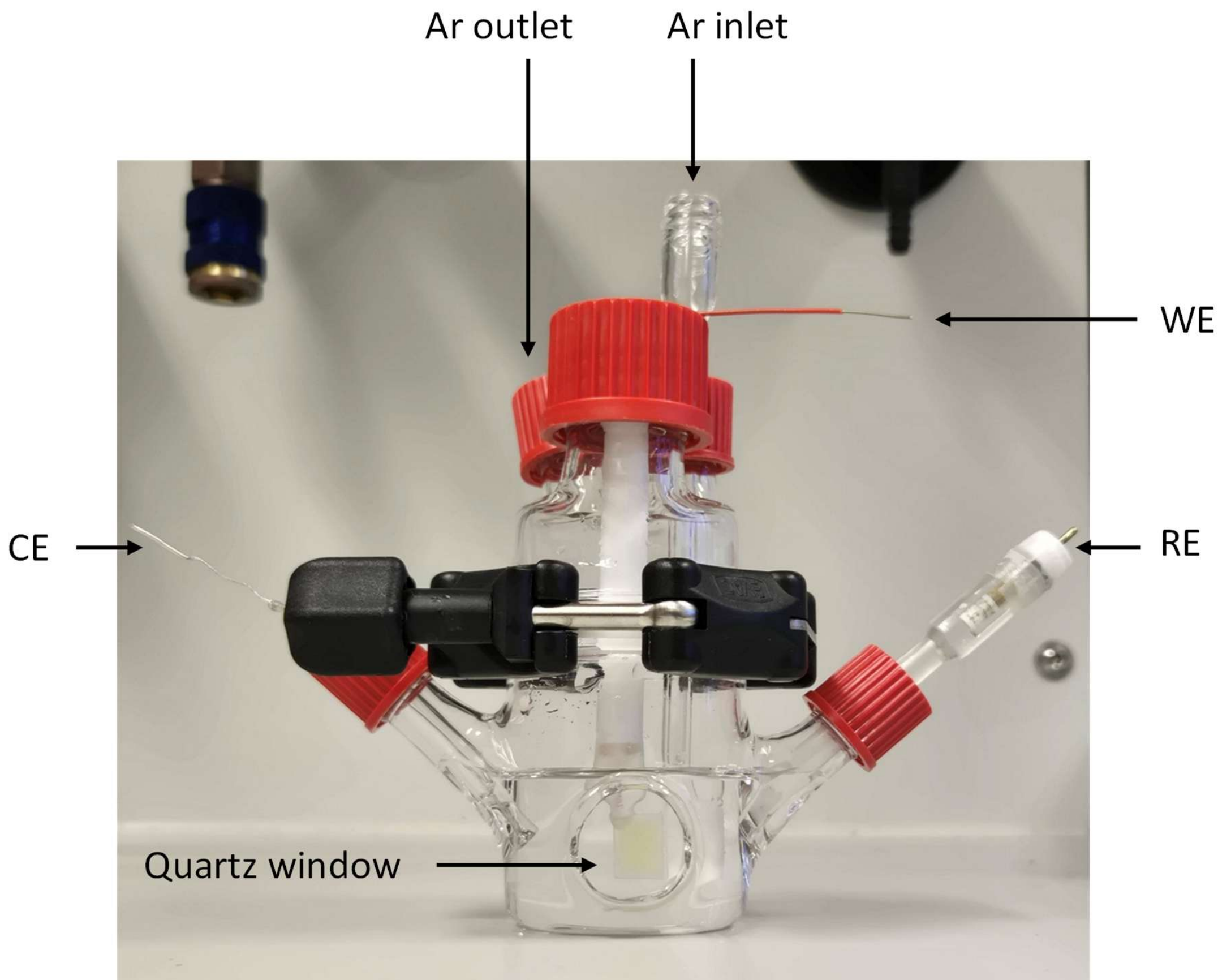

Figure S23. The three-electrode setup for the (Photo)electrochemical measurements.

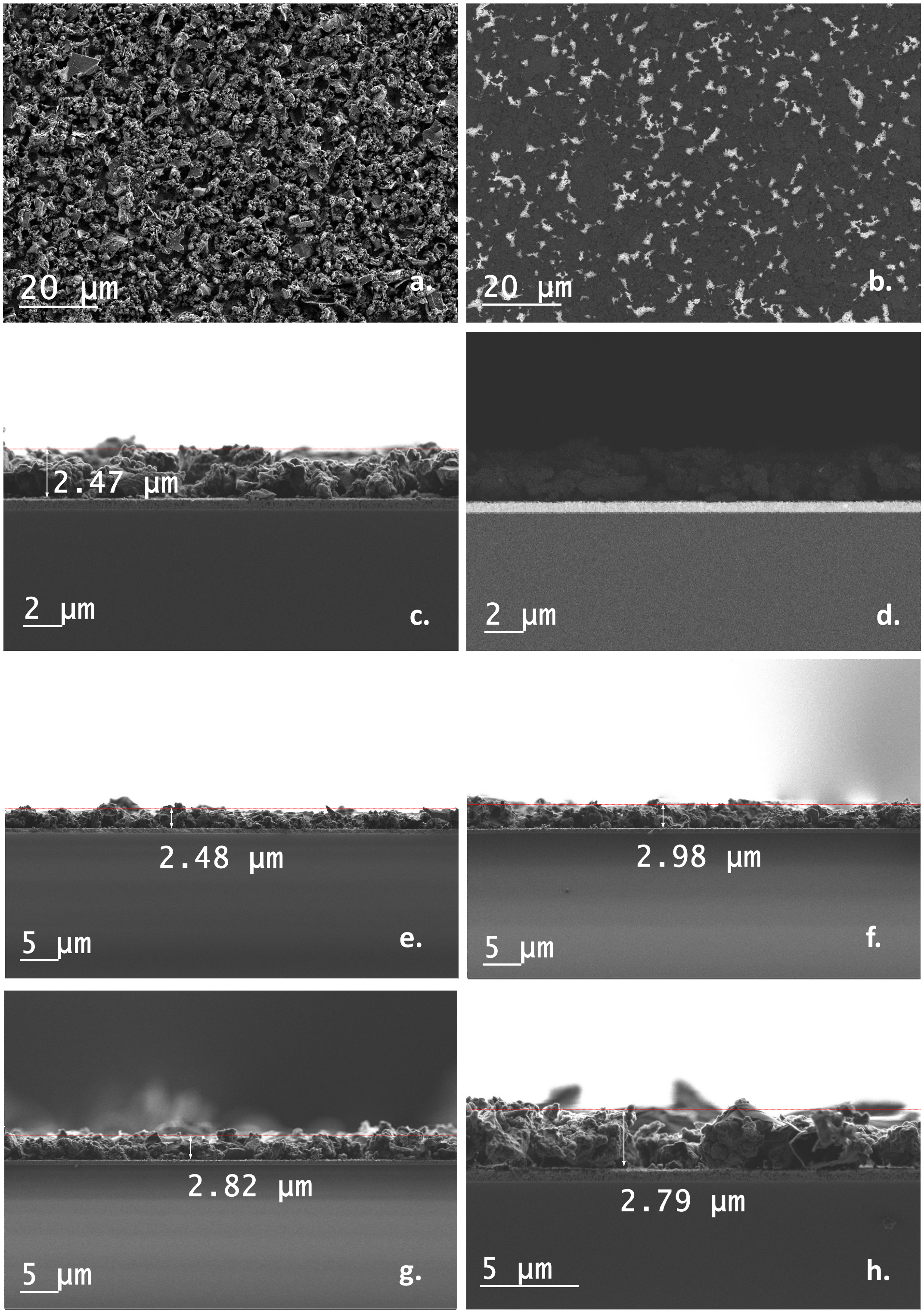

Figure S24. SEM images of the drop-cast CTF-Bpy films on an FTO-coated glass substrate from (a, b) the top and (c-h) from the side. Figure b and d are taken with the EsB (Energy and angle selective BSE) detector showing the material contrast. The other Figures are taken with the SE (secondary electron) detector. In Figure b, the bright areas denote the FTO parts that are not covered by the material. In Figure d, the bright parts denote FTO layer. Measurements in Figure c and e-h were taken at various locations in different drop-cast films, yielding an average film thickness of ca. 2.7 µm.

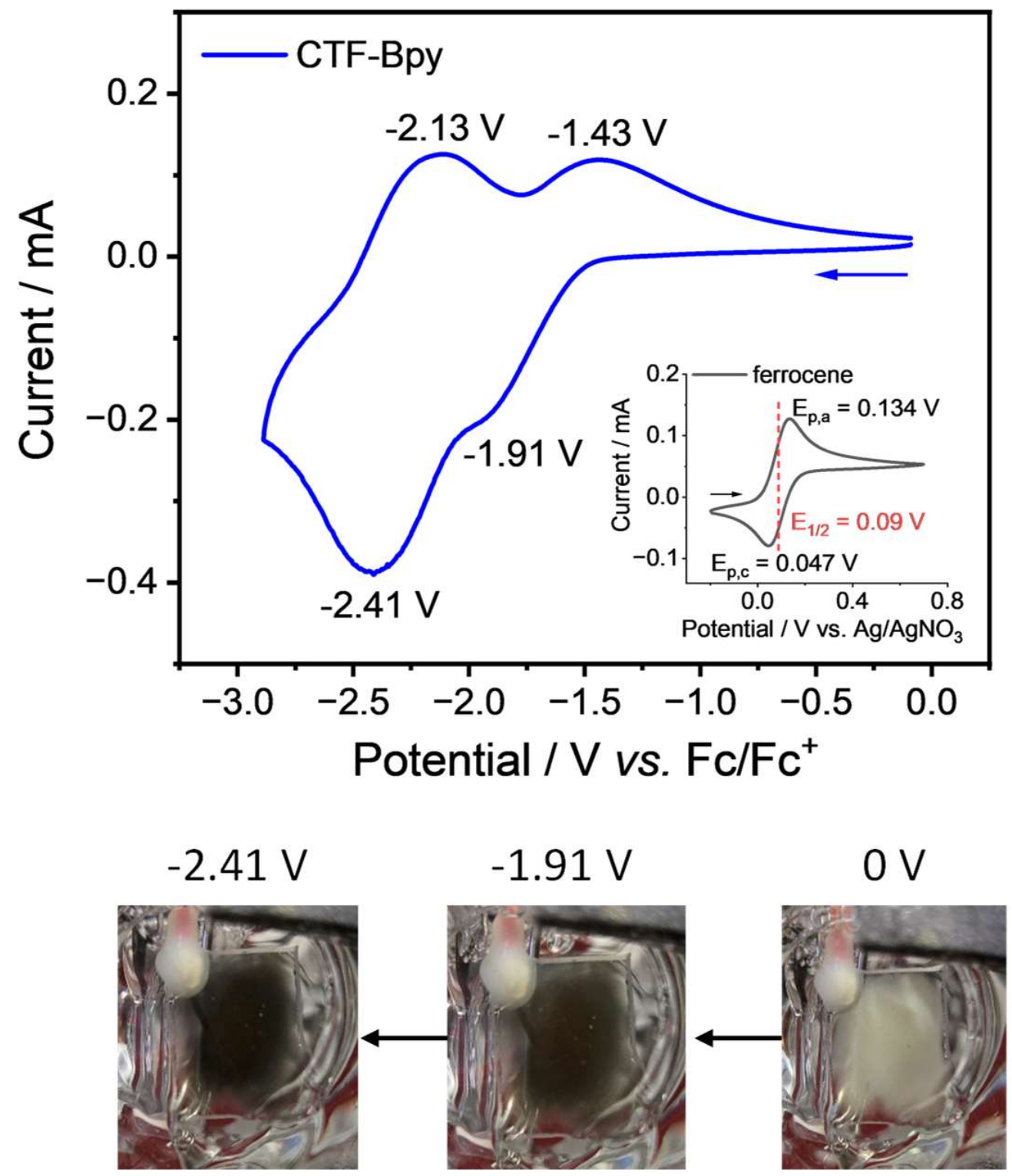

Figure S25. Cyclic voltammetry (CV) curve of CTF-Bpy on an FTO-coated glass electrode in dry acetonitrile containing 0.1 M $TBAClO_4$. Inset is the CV curve of ferrocene. Potentials measured are calibrated according to ferrocene. *Below the Figure are the photographs showing the color of CTF-Bpy film at 0 V, −1.91 V and −2.41 V, during the cathodic scan, respectively. CV tests were conducted at a scan rate of 50 mV s^{-1} under continuous Ar flow.

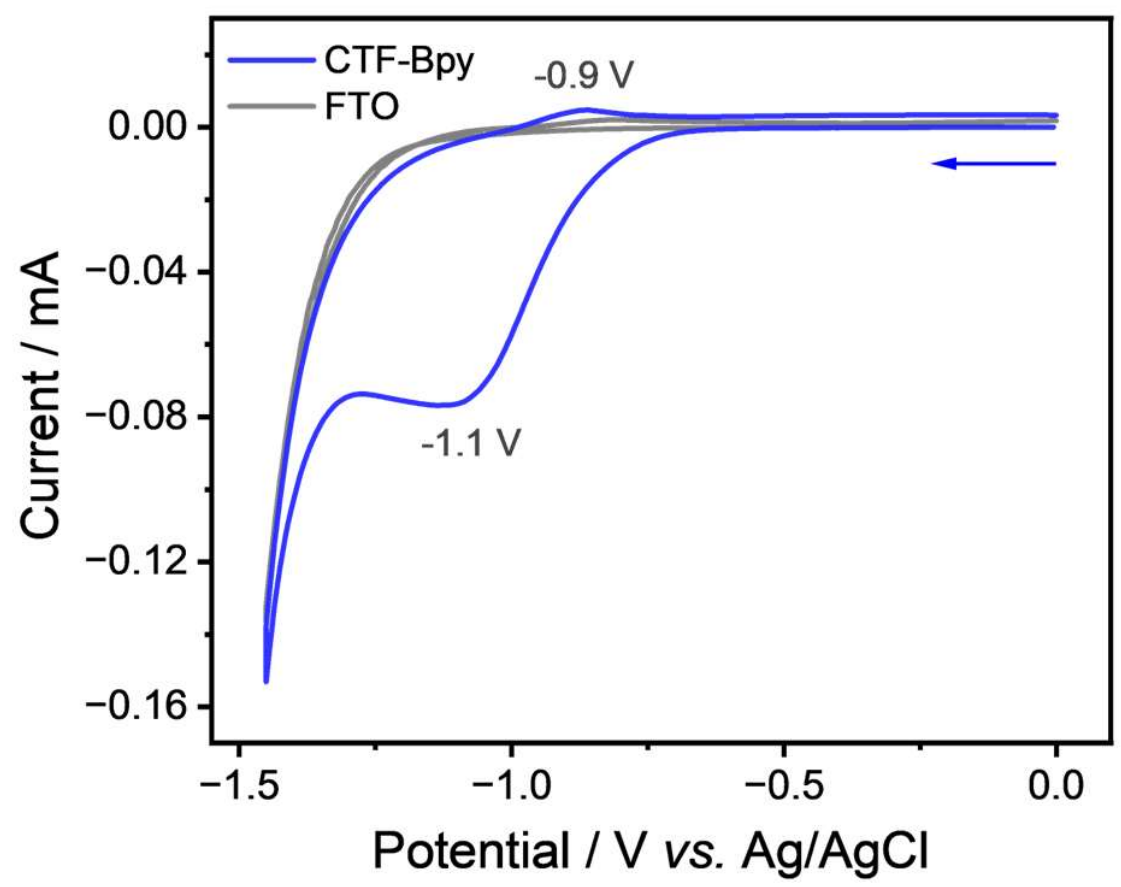

Figure S26. Comparison of CVs of CTF-Bpy on an FTO-coated glass electrode and a blank FTO-coated glass electrode confirms that the reduction/oxidation peaks at −1.1/−0.9 V originate from CTF-Bpy. CVs tests were recorded at 10 mV s^{-1} in 0.1 M KCl aqueous electrolyte within a potential window of 0 to −1.5 V vs Ag/AgCl under continuous Ar purging.

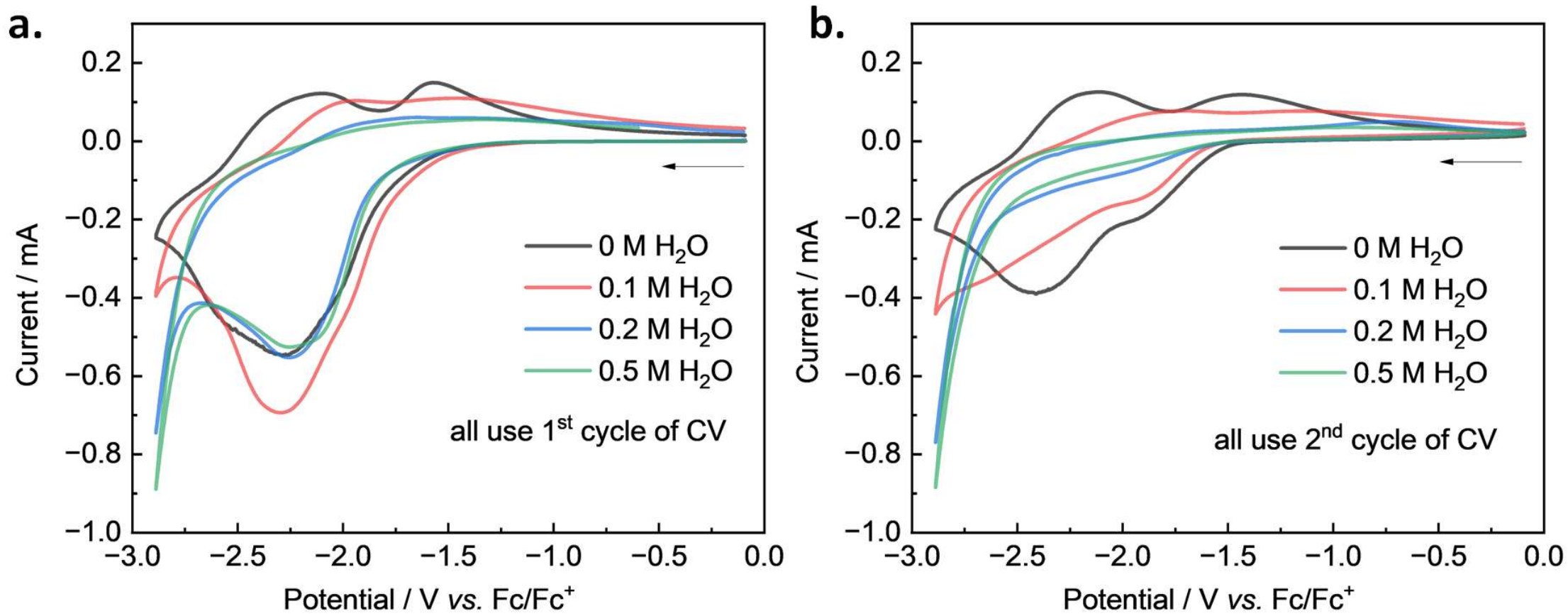

Figure S27. CV curves of CTF-Bpy on an FTO-coated glass electrode in dry acetonitrile containing 0.1 M $TBAClO_4$ with 0, 0.1, 0.2 or 0.5 M H_2O. (a) First and (b) Second CV cycles recorded during the measurements. CV measurements were conducted at a scan rate of 50 mV s^{-1} under continuous Ar flow, with a fresh electrode used for each measurement.

4.5 *In situ* EPR spectro-electrochemistry measurements

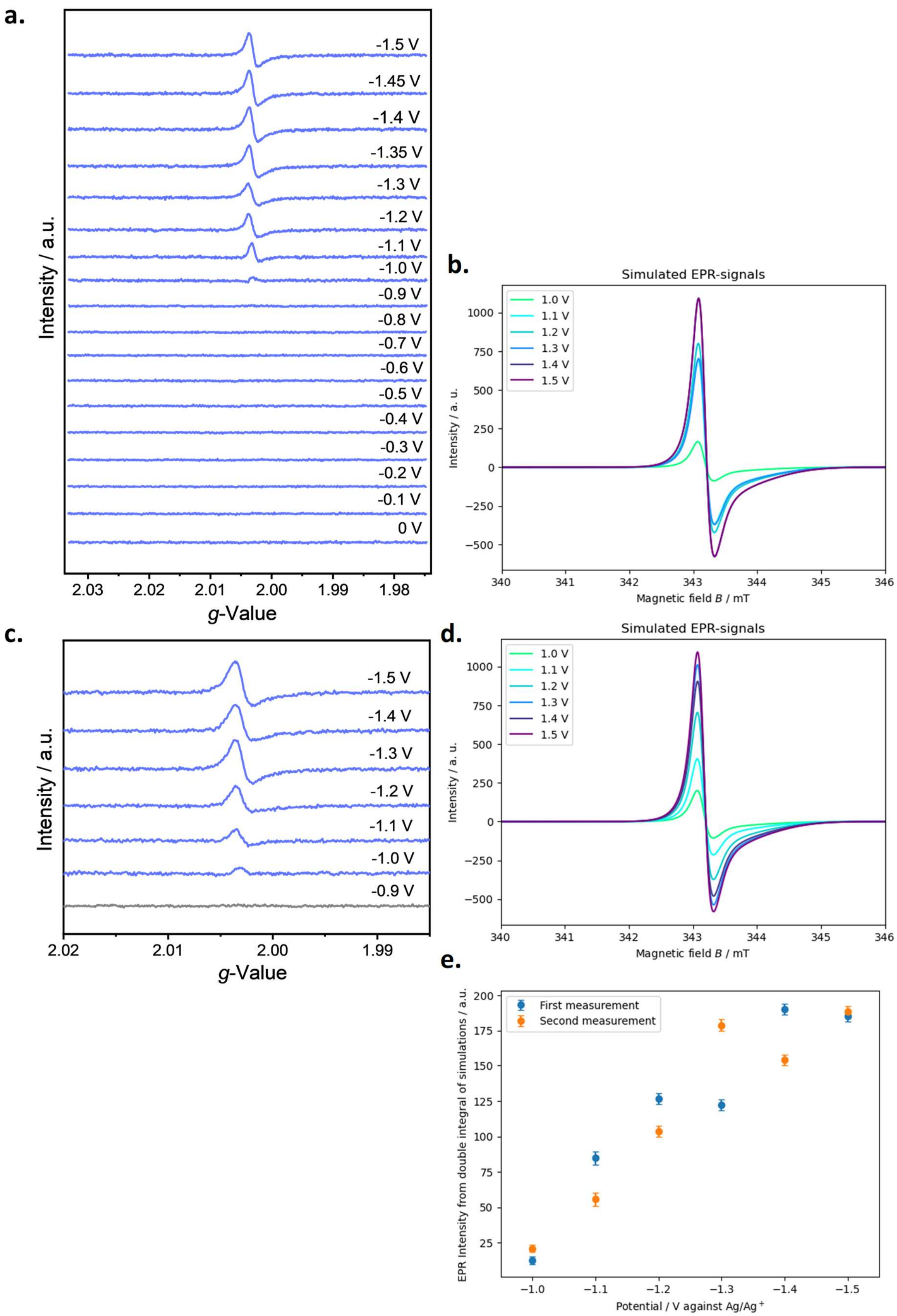

Figure S28. (a) EPR spectro-electrochemical experiments of CTF-Bpy in 0.5 M KCl aqueous electrolyte. Experiments were performed at a scan rate of 10 mV s^{-1}, and the EPR data was collected during the cathodic scan from 0 to −1.5 V versus Ag/AgCl. (b) Simulated EPR spectra of (a) in the potential range −1.0 V to −1.5 V. (c-d) Experimental (c) and the corresponding simulated (d) EPR spectra from a second measurement batch. The coated electrode was exposed to air (oxygen) and rinsed with water before the second measurement. (e) Potential dependence of double integral EPR intensity evaluated from the simulated EPR spectra in (b) (blue colour) and (d) (orange colour). The error is estimated based on the fitting of the experimental curve via numerical minimization of the error of the calculated area considering a confidence interval of 95 %. Additional potential sources of error, including sample movements in the resonator due to gas evolution near the solvent's potential-window limits, were not considered. EPR spectra in (a) and (c) are measured at a microwave frequency of 9.4226 GHz.

The less symmetric EPR signal observed in spectro-electrochemical measurements (**Figure S28a, c**) compared to the EPR signal obtained upon light illumination (**Figure 2c**) is likely due to the differences in measurement conditions. Specifically, the illuminated EPR spectrum of CTF-Bpy was collected from a suspension, whereas the spectra in **Figure S28a** and **Figure S28c** were collected using an electrode dip coated with CTF-Bpy.

5. Mechanistic investigation of the light-induced radical formation

5.1 PXRD under illumination

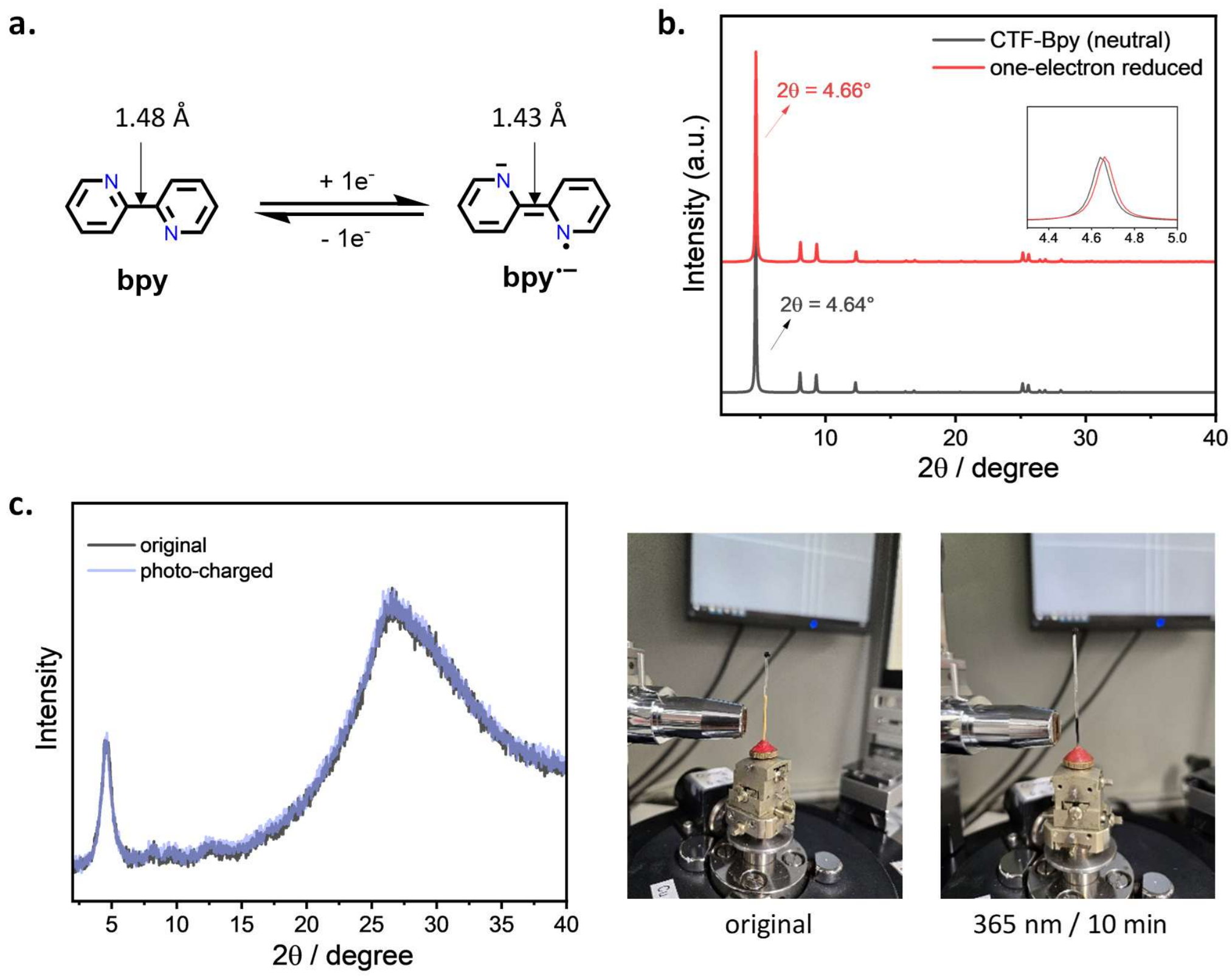

Figure S29. (a) Schematic of the reported C_{py}-C_{py} bond length change in 2,2'-bipyridine (bpy) upon one-electron reduction.[9] (b) Comparison of the simulated PXRD patterns of CTF-Bpy structural models based on the neutral versus one-electron reduced bpy. (c) Comparison of experimental PXRD patterns of CTF-Bpy before and after photocharging. Right: photograph of CTF-Bpy in a capillary before and after illumination. PXRD samples for the photocharging experiment were prepared by suspending CTF-Bpy in 20 mM 4-MBA aqueous solution under Ar atmosphere.

In the Pawley refined model of CTF-Bpy, the C_{py}-C_{py} bond length is 1.414 Å, shorter than in molecular 2,2'-bipyridine (bpy), which is attributed to extended conjugation within the framework. Following the bond length variation observed in molecular bpy, the C_{py}-C_{py} bond in the photocharged CTF-Bpy was fixed at 1.364 Å during geometry optimization, while changes in other bond lengths were neglected as they are comparatively less significant.[9]

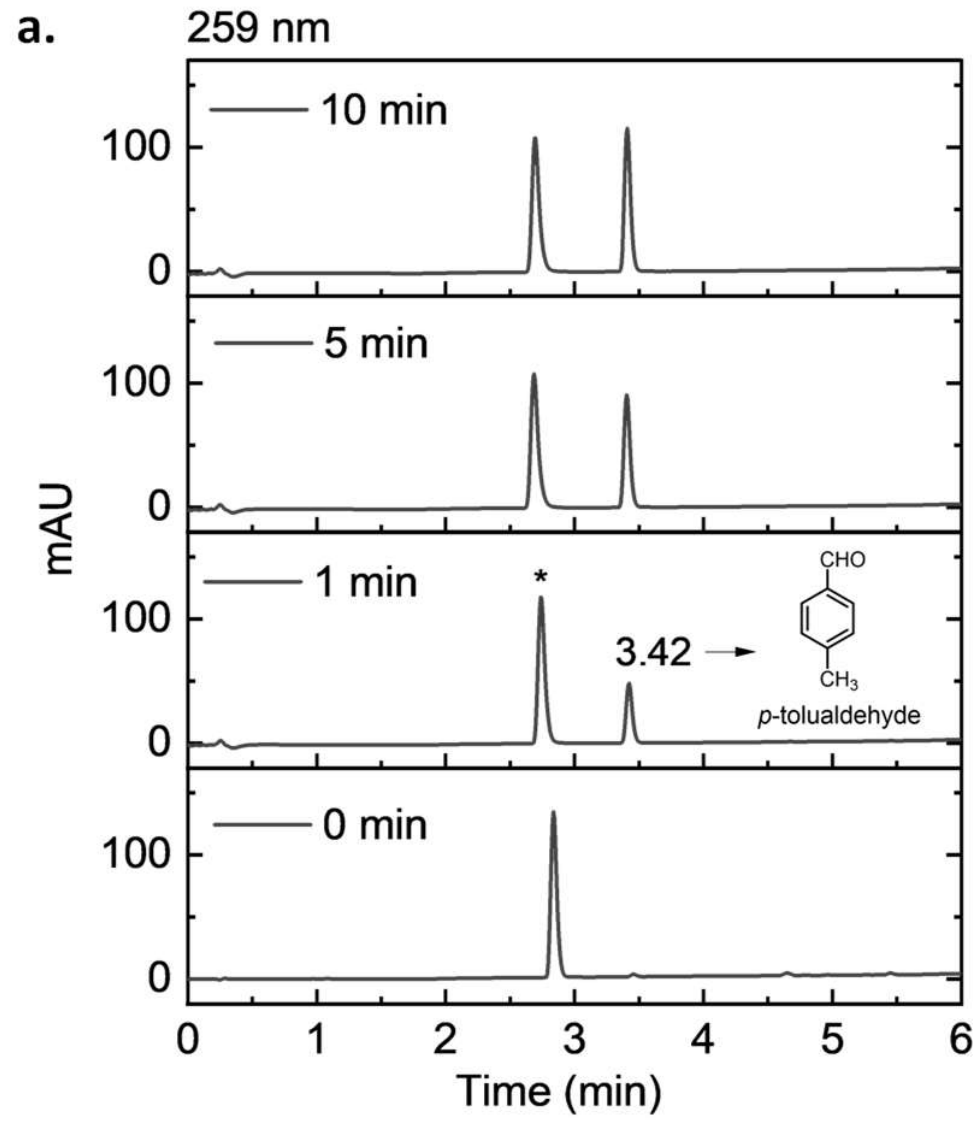

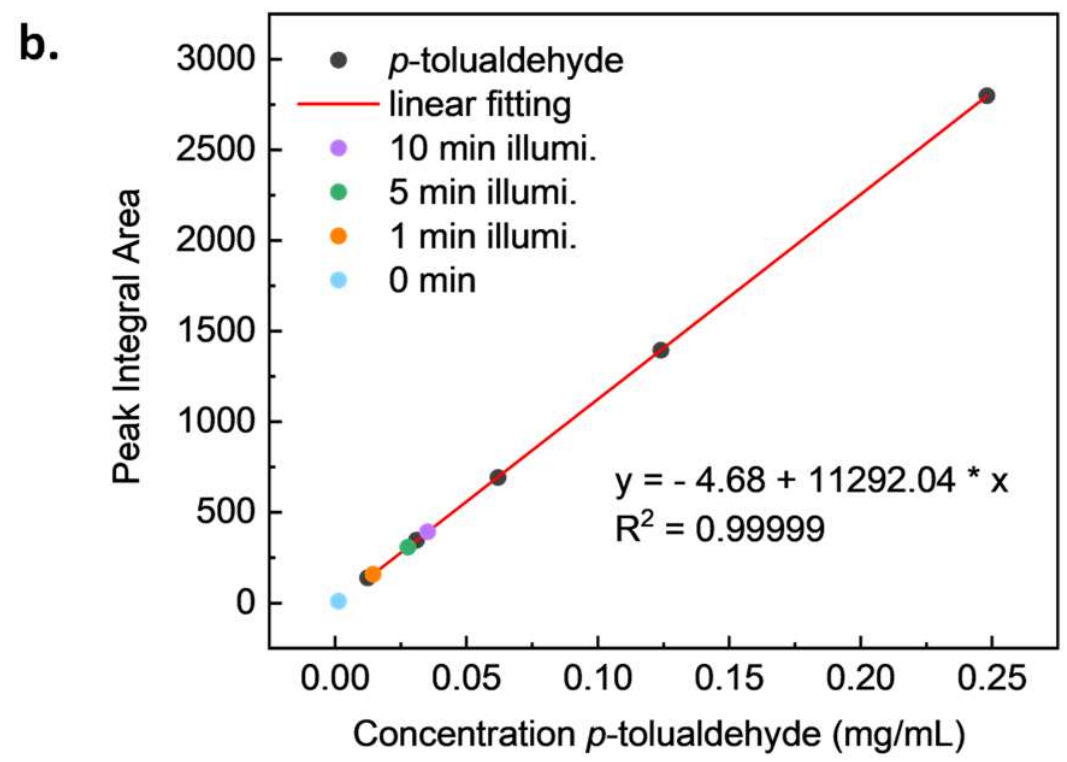

c.

Illu. time	Area	Fitted amount of p-tolualdehyde	
		mg/mL	mmol/L
0	9.9953	0.0013	0.0108
1	158.2567	0.0144	0.1199
5	308.0813	0.0277	0.2305
10	393.2615	0.0352	0.2929

Figure S30. a) HPLC chromatogram of CTF-Bpy aqueous suspension (1 mg/mL) containing 20 mM 4-methylbenzyl alcohol (4-MBA) before irradiation (0 min) and after 1, 5 and 10 min of irradiation under 365 nm UV light. HPLC chromatogram recorded at a detection wavelength of 259 nm. The filtered reaction mixture was directly used for HPLC analysis. *The asterisk denotes the peak corresponding to 4-MBA. (b) Calibration curve for the HPLC quantification of *p*-tolualdehyde constructed using standard solutions of known concentrations. The peak area was plotted against the analyte concentration, and the peak areas of *p*-tolualdehyde obtained from the photocharged CTF-Bpy suspensions were fitted to the calibration curve to determine the corresponding concentrations. (c) Summary table of the quantified *p*-tolualdehyde concentrations obtained from the calibration curve. HPLC chromatogram was performed using a C_{18} column maintained at 40 °C with a gradient elution of H_2O/acetonitrile containing 0.1% formic acid as the mobile phase. The trace amount of *p*-tolualdehyde detected prior to irradiation can be attributed to the activity of CTF-Bpy under ambient conditions toward O_2 reduction to H_2O_2, where 4-MBA serves as an electron donor (i.e., ambient light and O_2 from air), consistent with the observation from the dark H_2O_2 measurements (**Figure S48**).

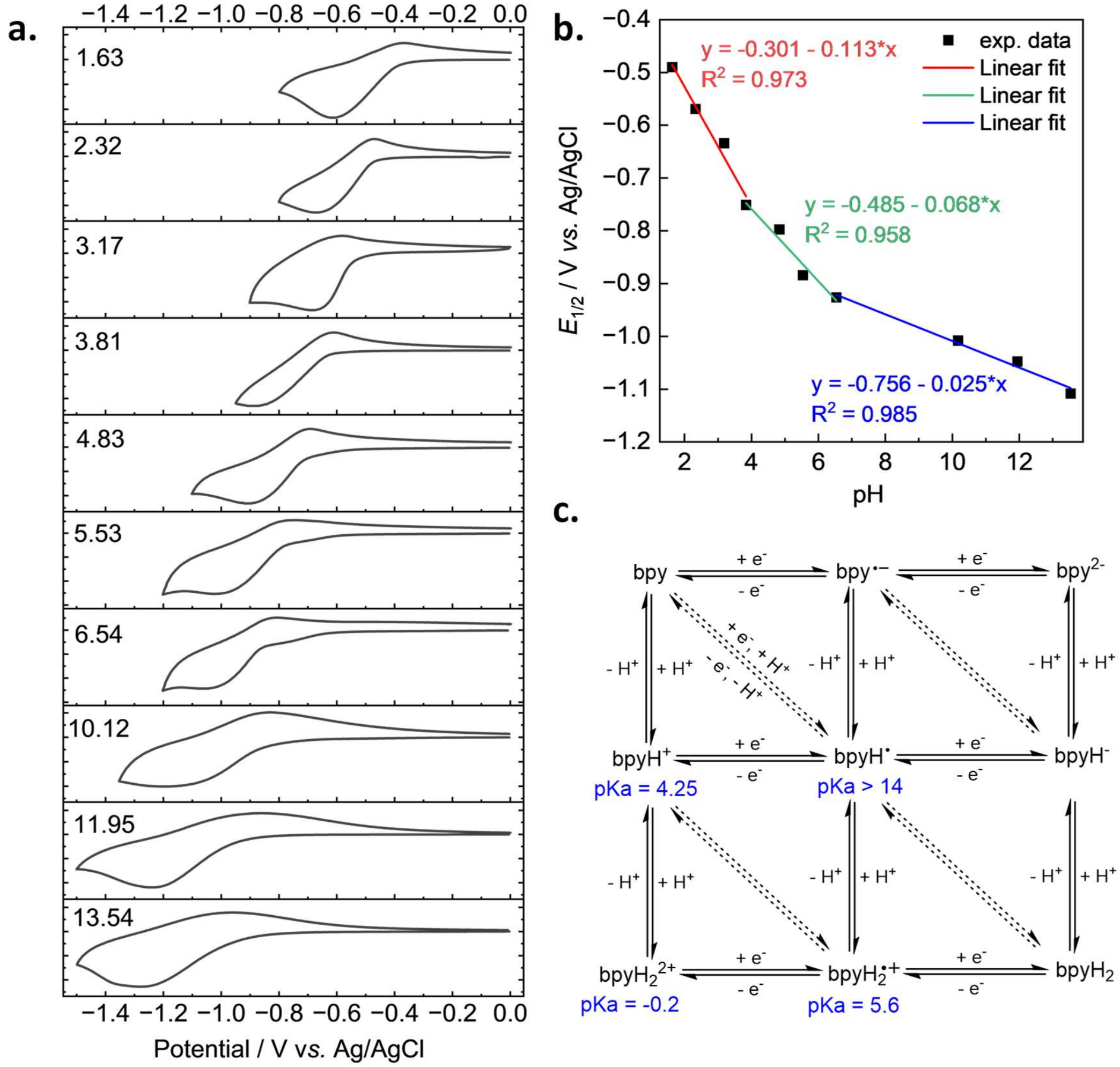

Figure S31. (a) CVs of CTF-Bpy on an FTO-coated glass electrode in aqueous 0.1 M KCl solutions at various pH values. (b) Pourbaix diagrams of CTF-Bpy. Linear lines are overlayed as fitting with the Nernst equation. (c) Nine-membered square scheme for 2,2'-bipyridine (bpy), showing the proton-dependent stepwise and (solid lines) concerted (dashed lines) proton-coupled electron transfer routes.[10] Electrolytes at pH 1.63, 2.32, and 3.17 were prepared using HCl; pH 3.81 and 4.83 using 0.1 M acetate buffer; pH 5.53 and 6.54 using 0.1 M phosphate buffer; pH 10.12 using 0.1 M glycine/NaOH buffer; and pH 11.95 and 13.54 using KOH. CV measurements were conducted at a scan rate of 50 mV s^{-1} under continuous Ar flow, with a fresh electrode used for each measurement.

5.2 Raman spectra

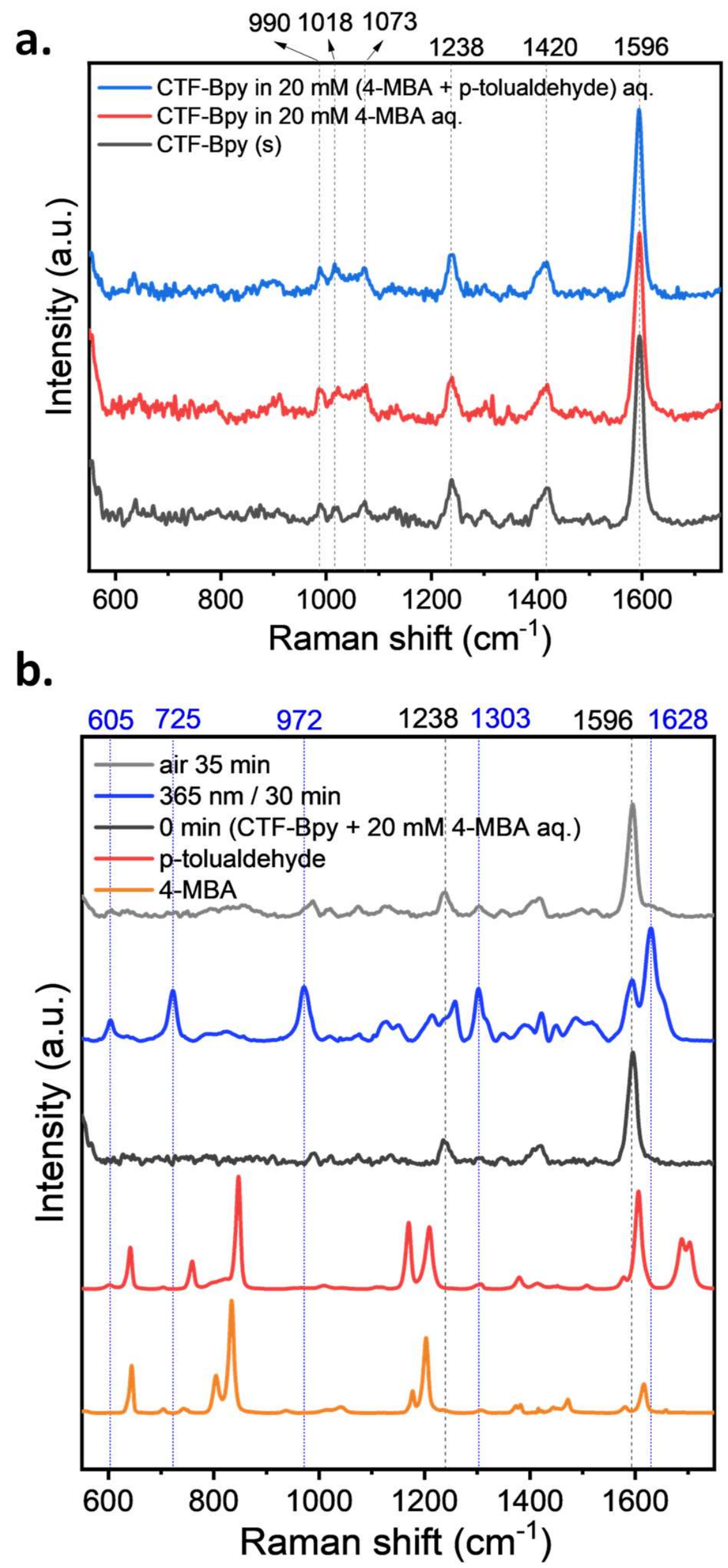

Figure S32. (a) Raman spectra of CTF-Bpy measured in solid state, aqueous suspension with 20 mM 4-MBA and aqueous suspension in a mixture of ''20 mM 4-MBA + 20 mM *p*-tolualdehyde'' in water. (b) Comparison of Raman spectra of 4-MBA, *p*-tolualdehyde and CTF-Bpy aqueous suspension with 20 mM 4-MBA before illumination (0 min), after illumination (30 min) and upon 35 min air exposure (air 35 min). Characteristic bands corresponding to the pristine CTF-Bpy and predominant band changes upon irradiation are marked in grey and blue dash lines, respectively.

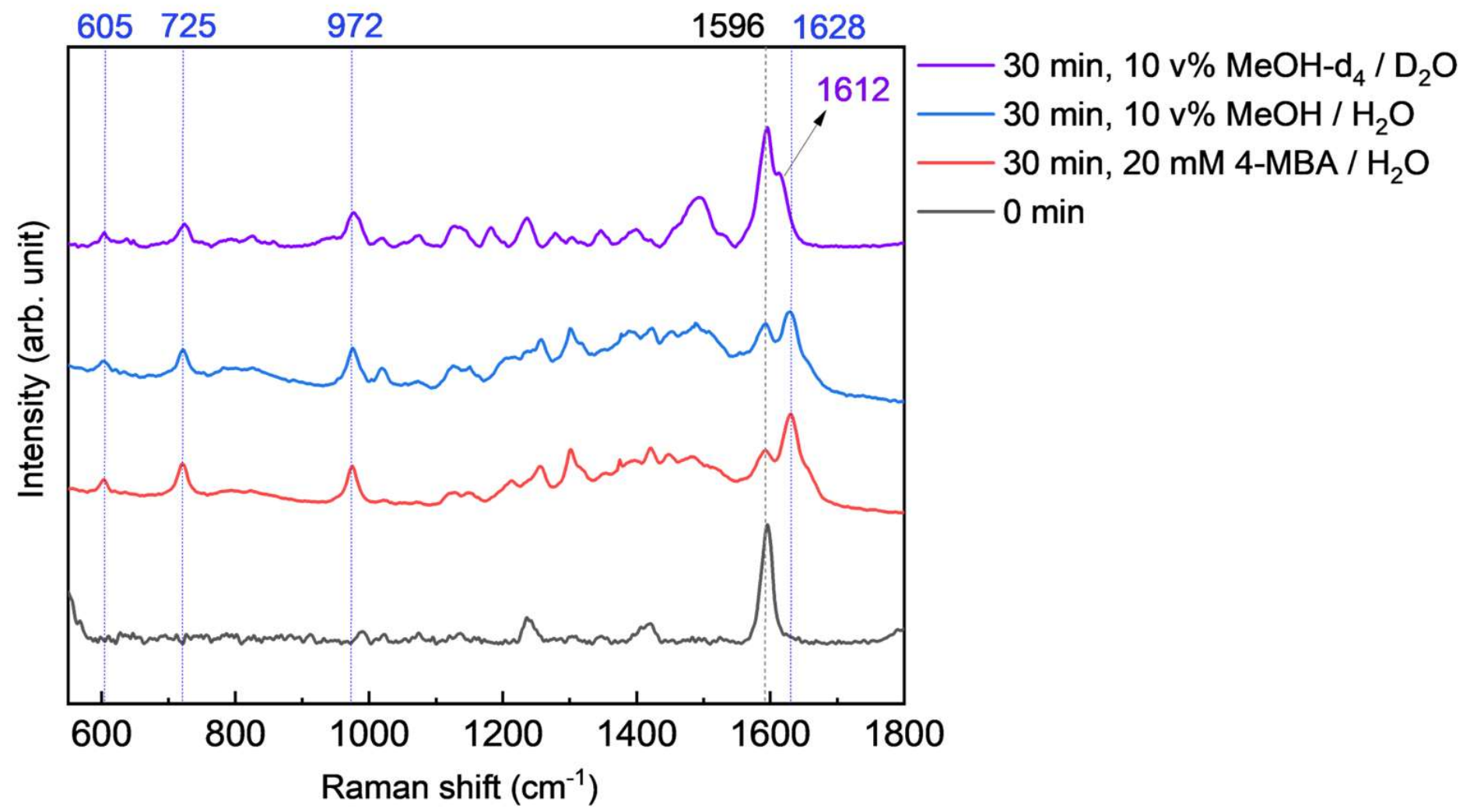

Figure S33. Raman spectra of CTF-Bpy recorded before (0 min, black) and after 30 min of irradiation with 365 nm UV light in (red) 20 mM 4-MBA aqueous solution, (blue) 10 v% MeOH in H_2O and (purple) 10 v% MeOH-d_4 in D_2O. MeOH was selected as the sacrificial electron donor for the isotope-effect study because a deuterated analogue of 4-MBA was not readily available.

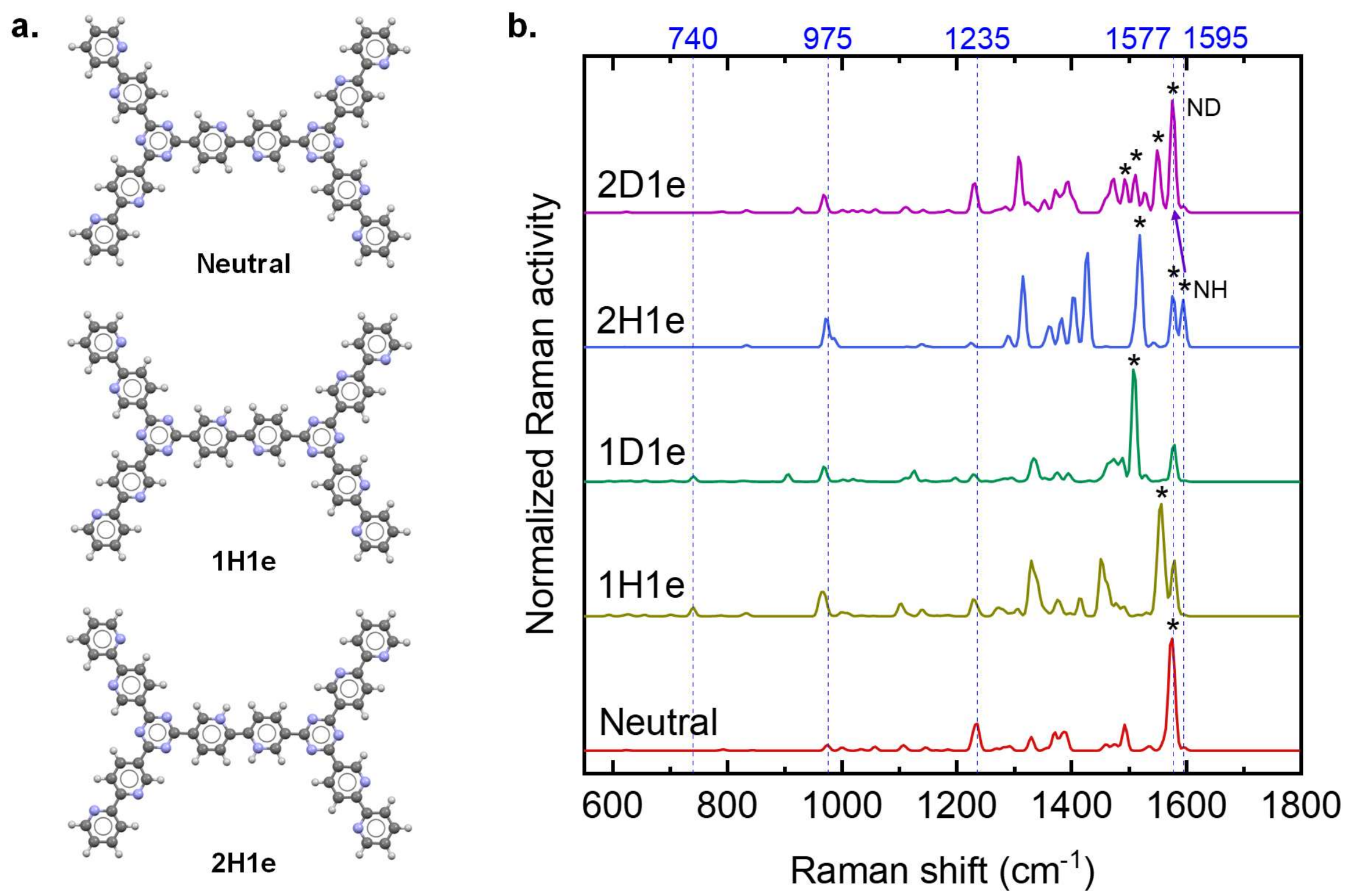

Figure S34. (a) Geometry-optimized CTF-Bpy fragment models used for the Raman spectral calculations. For the protonated models, protonation was introduced only at the central bpy moiety for the calculation. (b) Simulated Raman spectra obtained from DFT calculations. The bands labeled with asterisk (*) indicate they are mainly contributed from stretching modes at the central bpy moiety in the fragment model. DFT calculations were performed using ORCA 6.0.1 at the B3LYP/def2-TZVP levels of theory. The band shift associated with the deuteration effect is labeled. The calculated vibrational frequencies were scaled by a factor of 0.967 to correct for systematic overestimation. Note that the four terminal hydrogen atoms were assigned with atomic mass of 10000 instead of 1 to minimize the termination effect on the simulated Raman spectra.

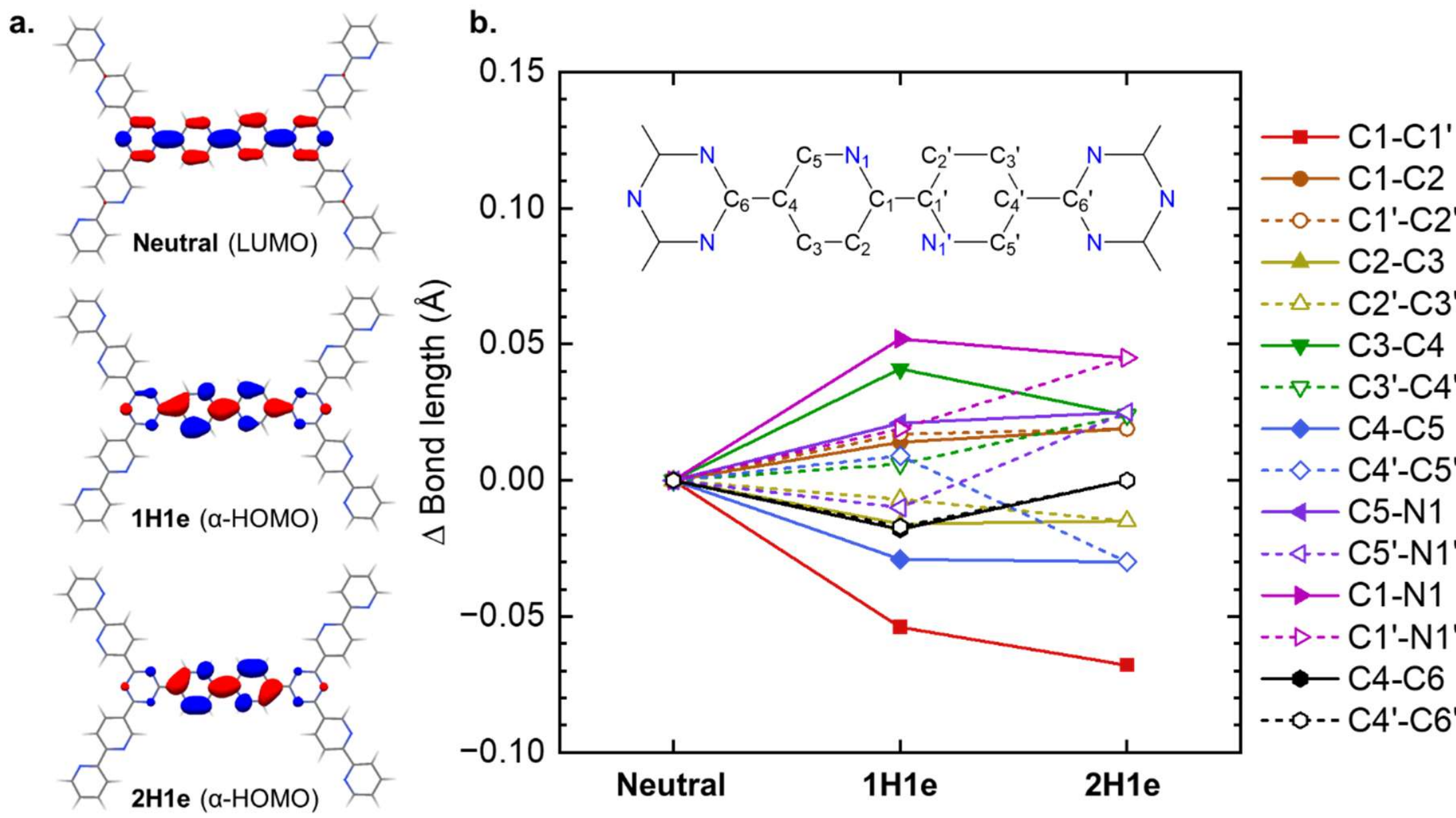

Figure S35. (a) Selected molecular orbitals of fragments models. (b) Change of bond length at the central bpy unit in the optimized geometry of neutral, 1H1e, and 2H1e models.

Table S1. Band assignments of the experimental Raman spectra of the pristine (0 min) and photocharged (365 nm / 30 min) CTF-Bpy shown in **Figure S32 and S33**, together with the simulated Raman spectra shown in **Figure S34**.

Description	CTF-Bpy Experimental			Calculated				
	0 min	30 min (H)	30 min (D)	Neutral	1H1e	1D1e	2H1e	2D1e
[b]C1-C1'; C2-C3 bond str. on 2H1e bpy; NH, CH in-plane bending on 2H1e bpy;		1628					1595	
[b]2D1e bpy ring str.; ND, CH in-plane bending on 2D1e bpy;			1612					1577
[a](neutral) bpy ring str.; C-H in-plane bending;	1596	1596	1596	1577	1580	1580	1577	1577
[b]1H1e bpy ring str.; NH, CH in-plane bending on 1H1e bpy;					1558			
[b]1D1e bpy ring str.; ND, CH in-plane bending on 1D1e bpy;						1509		
[a]triazine ring str; $C_{triazine}$-C_{bpy} bond str.; C-H in-plane bending;	1420	1420	1420	1388				
[b]reduced bpy ring str., e.g., 2H1e/2D1e CH, NH in-plane bending on reduced bpy;		1303					1316	1308
[a](neutral) bpy ring str.; CH in-plane bending;	1238			1236	1236	1236		1233
[a]ring str., CH in-plane deformation	1073	1073	1073					
[a]ring str.	1018	1018	1018					
[a]Triazine ring deformation;	990			975			972	968
[a,b]Triazine ring in-plane deformation; reduced bpy ring deformation,		972	972		968	968		
[a,b]reduced bpy ring deformation,		725	725		740	741		
[a,b]reduced bpy ring deformation		605	605					

*Raman bands are assigned according to the literature[a] and DFT calculations[b] shown in Figure S34-35.[11, 12] The main band changes on the Raman spectra of CTF-Bpy upon illumination are marked by blue (H) and purple (D) color. Characteristic Raman bands of pristine CTF-Bpy are shown in dark color.

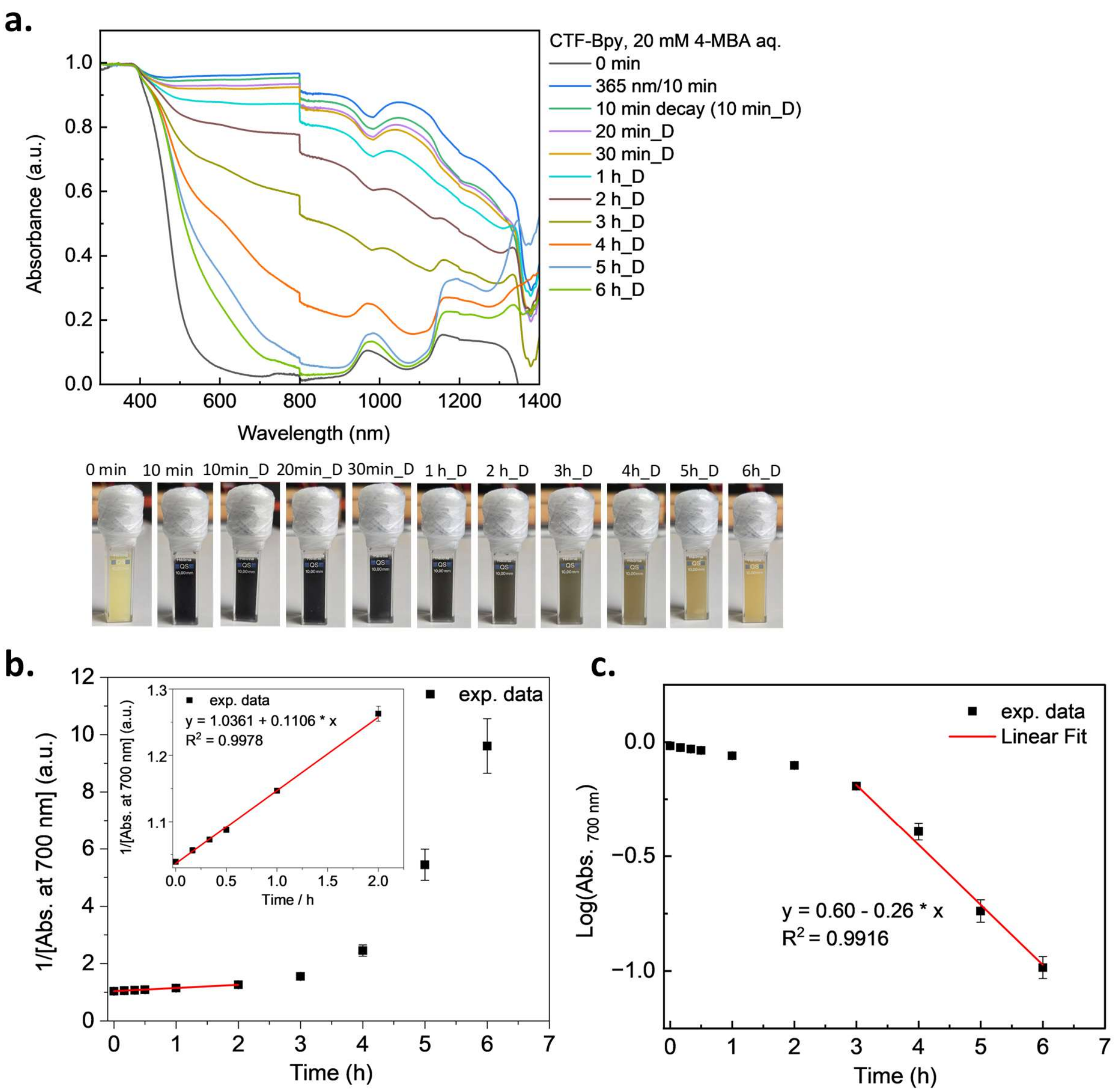

Figure S36. (a) UV–vis–NIR absorption spectra of an Ar-purged CTF-Bpy aqueous suspension (1 mg/3 mL) containing 20 mM 4-MBA before irradiation, after 10 min of 365 nm UV irradiation, and during subsequent dark decay over 6 h after the light was switched off (top). The corresponding photographs of the suspension at each time point are shown below. (b-c) Kinetic analysis of radical decay based on the absorbance data shown in (a). The decay during the first 2 h in the dark is consistent with second-order kinetics (b), whereas the decay during the final 3 h is well described by first-order kinetics (c).

6. Photoelectrochemistry investigation

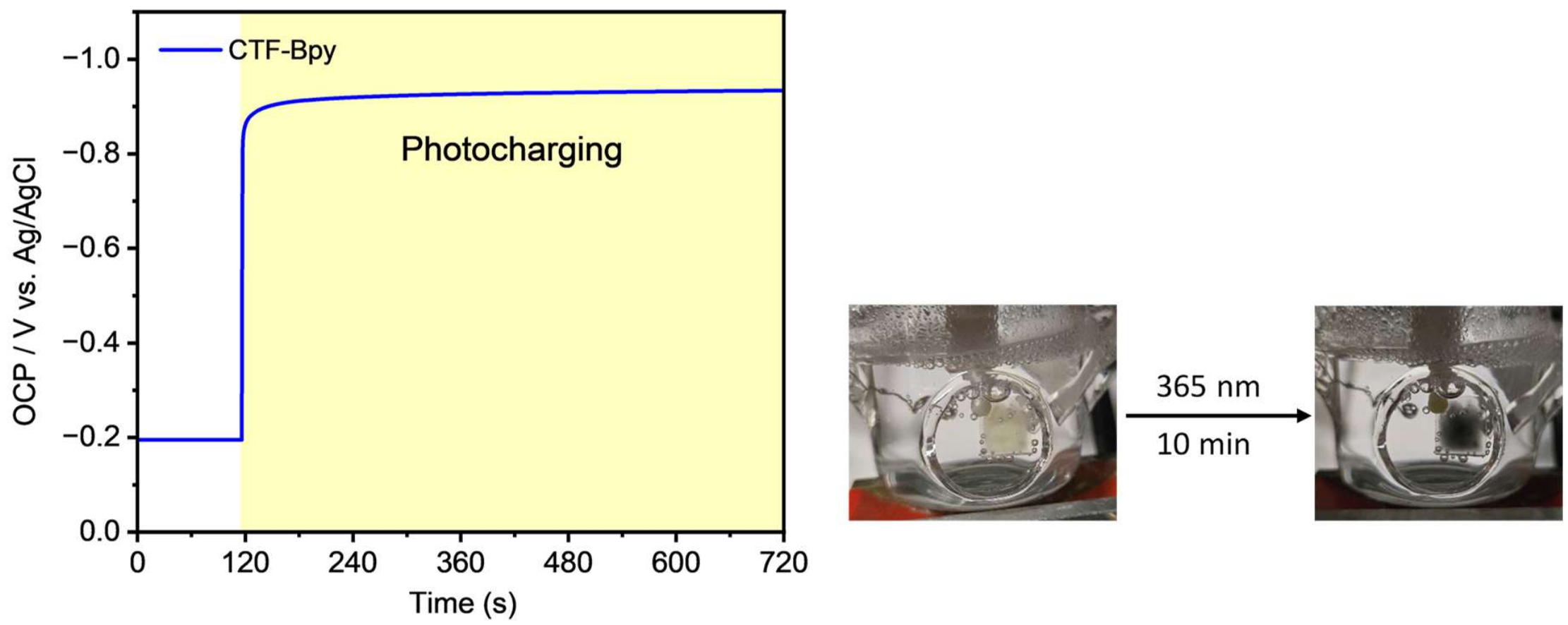

Figure S37. Open circuit potential (OCP) of CTF-Bpy film on an FTO-coated glass electrode upon 365 nm UV illumination for 10 min. Right: Photographs showing the film's colour change from the original yellow to black after 10 min illumination. Measurement was performed in 20 mM 4-MBA aqueous electrolyte.

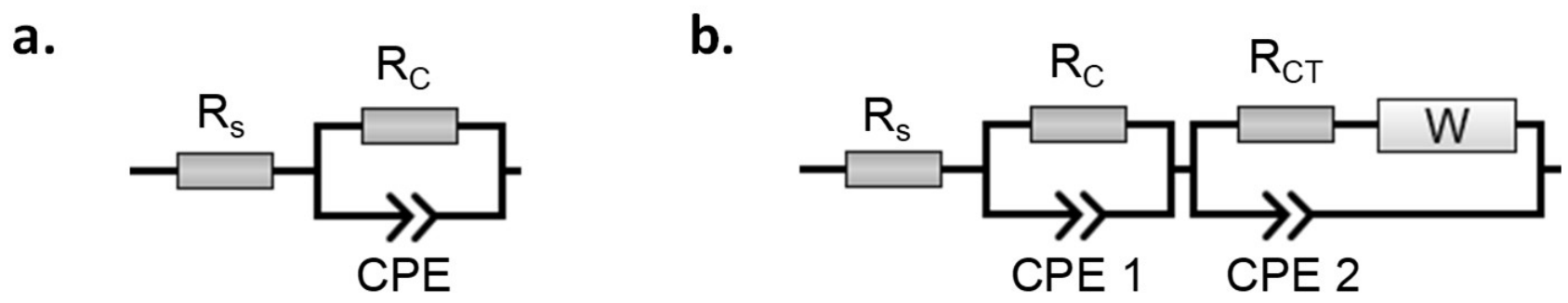

Figure S38. Simulated equivalent circuit models used for fitting the experimental impedance data of the CTF-Bpy photoanode (a) in the dark (0 min) and (b) after 10 min illumination by 365 nm UV light.

Table S2. Fitting results of the impedance measurement in the dark as shown in **Figure 4e**.

Parameters	Value	Error / %
R_S (Ω)	64.1	3.9
R_C (Ω)	1.61E+08	1.2
CPE Q 1	1.61E-05	0.01
CPE Alpha 1	0.99	0.0043

Table S3. Fitting results of the impedance measurement after 10 min 365 nm UV illumination as shown in **Figure 4e**.

Parameters	Value	Error / %
R_S (Ω)	45.4	9
R_C (Ω)	1.03E+04	13
CPE Q 1	2.65E-04	16
CPE Alpha 1	0.669	5.8
R_{CT} (Ω)	3.68E+03	19
Warburg Coeff. 1	2.75E+03	3
CPE Q 2	4.07E-05	4.4
CPE Alpha 2	0.904	4.4

7. Solar battery function

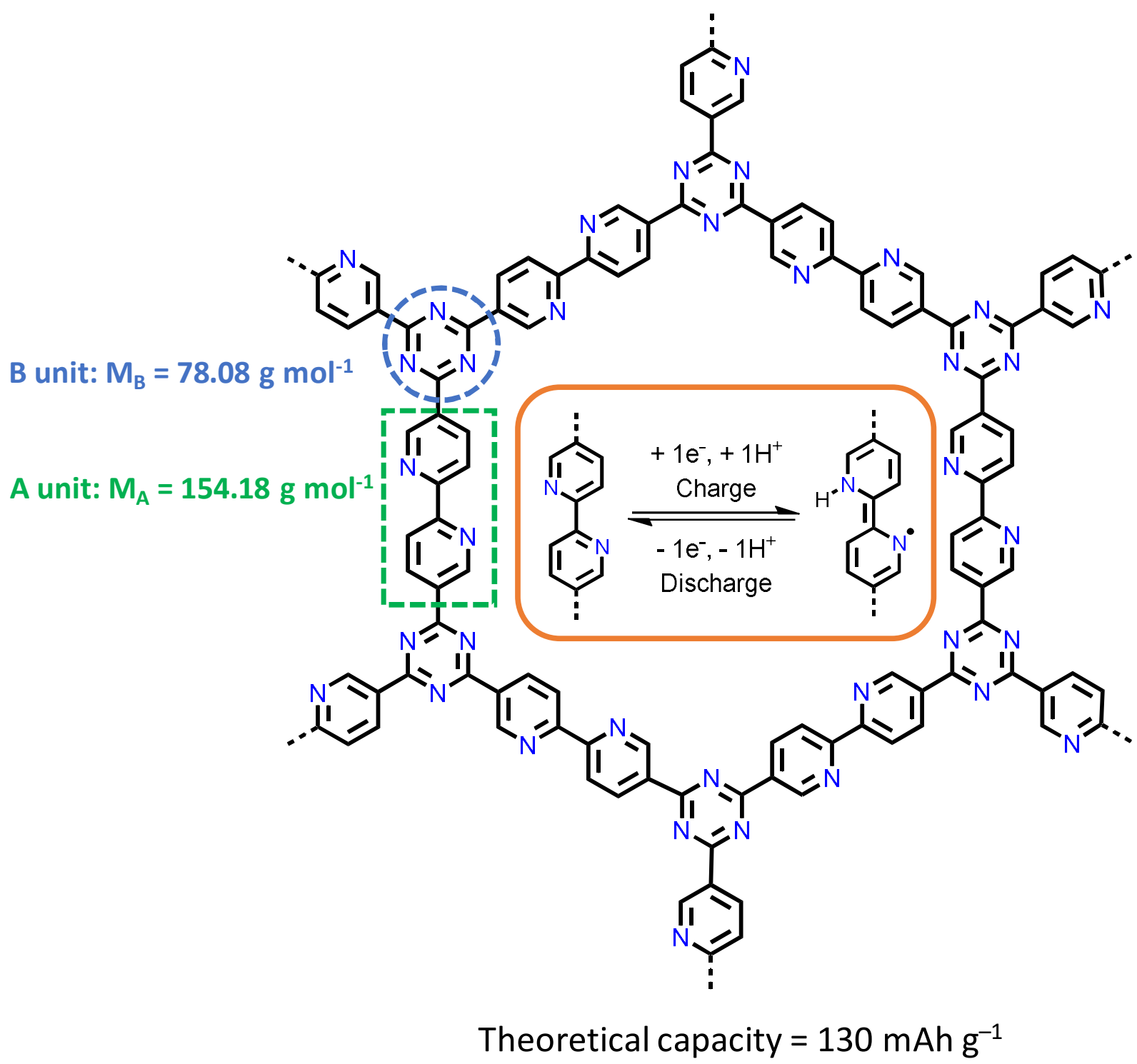

Figure S39. Theoretical capacity of CTF-Bpy calculated based on the one-electron reduction of bpy unit.[13, 14]

Equation for the calculation of the theoretical capacity:

$$C_{spec}\,(\mathrm{mAh\ g^{-1}}) = \frac{n \times F}{M_W \times 3.6} = \frac{n \times 96485\ (\mathrm{C\ mol^{-1}})}{M_W\ (\mathrm{g\ mol^{-1}}) \times 3.6}$$

In the above equation, where n is the number of electrons transferred per redox reaction. F is the Faraday constant, and Mw is the molar weight of the repeating unit of the organic component.[13, 14]

The repeating unit in CTF-Bpy consists of 1/2 of a A unit (M_A) and 1/3 of a B unit (M_B). Hence, the molecular weight of the repeating unit in CTF-Bpy is 103.12 g mol^{-1} ($C_6H_3N_2$). The number of electrons (n) involved in the repeating unit is equal to 1/2, as 1 electron is involved in each bpy unit. Therefore, using the Equation above, the theoretical capacity of CTF-Bpy is calculated to be 130 mAh g^{-1}.

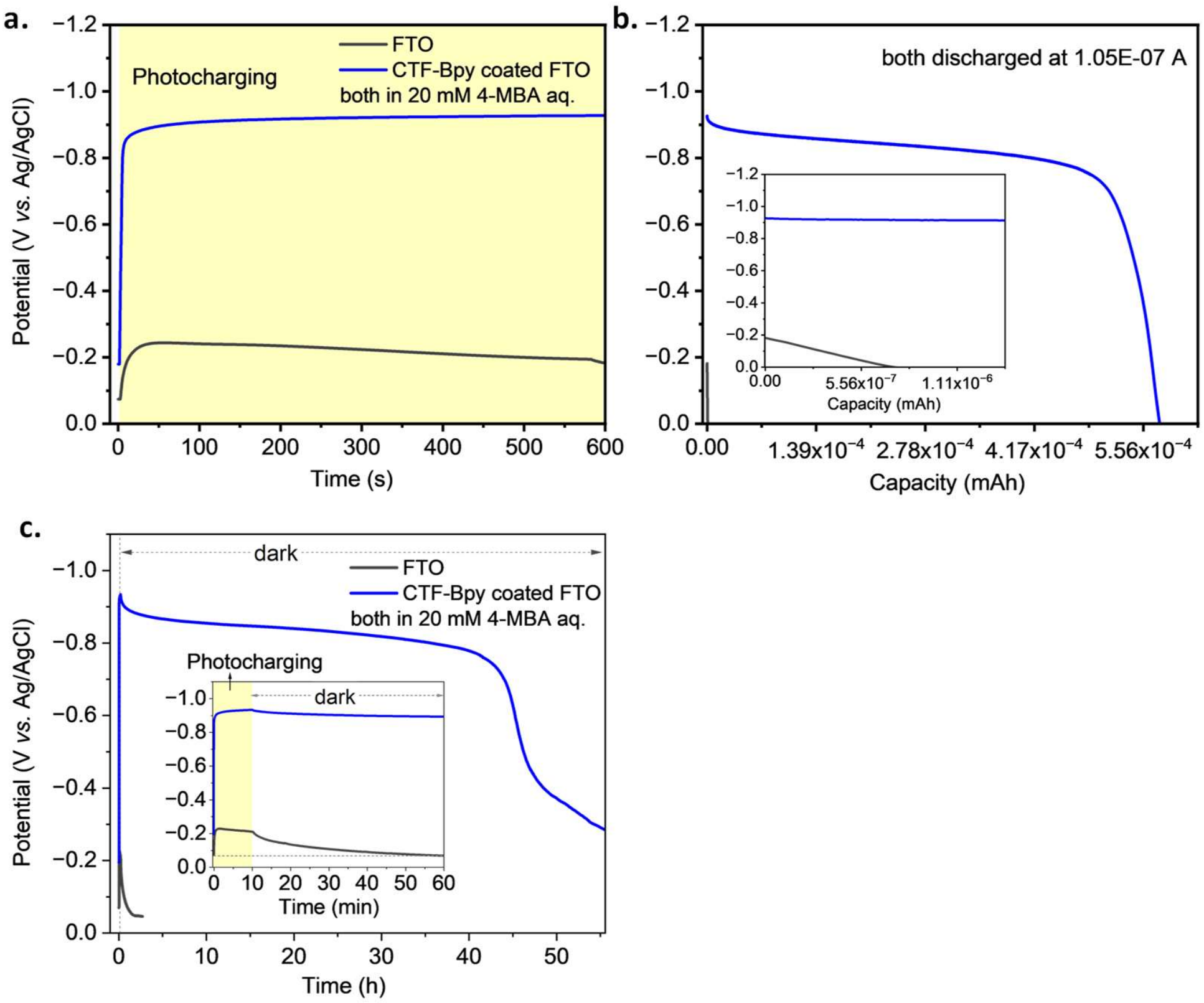

Figure S40. (a) Photocharging of bare FTO and CTF-Bpy coated FTO photoanode under OCP conditions with 365 nm UV illumination ($P_{nominal}$ = 237 mW cm^{-2}). (b) Subsequent electric discharge in the dark at a current density of 1.5 mA g^{-1}. (c) OCP stability after 10 min 365 nm UV light illumination, demonstrating the stability of stored electrons under dark conditions. The inset shows the OCP during 10 min photocharging. All measurements were performed in 20 mM 4-MBA aqueous electrolyte under continuous Ar purging.

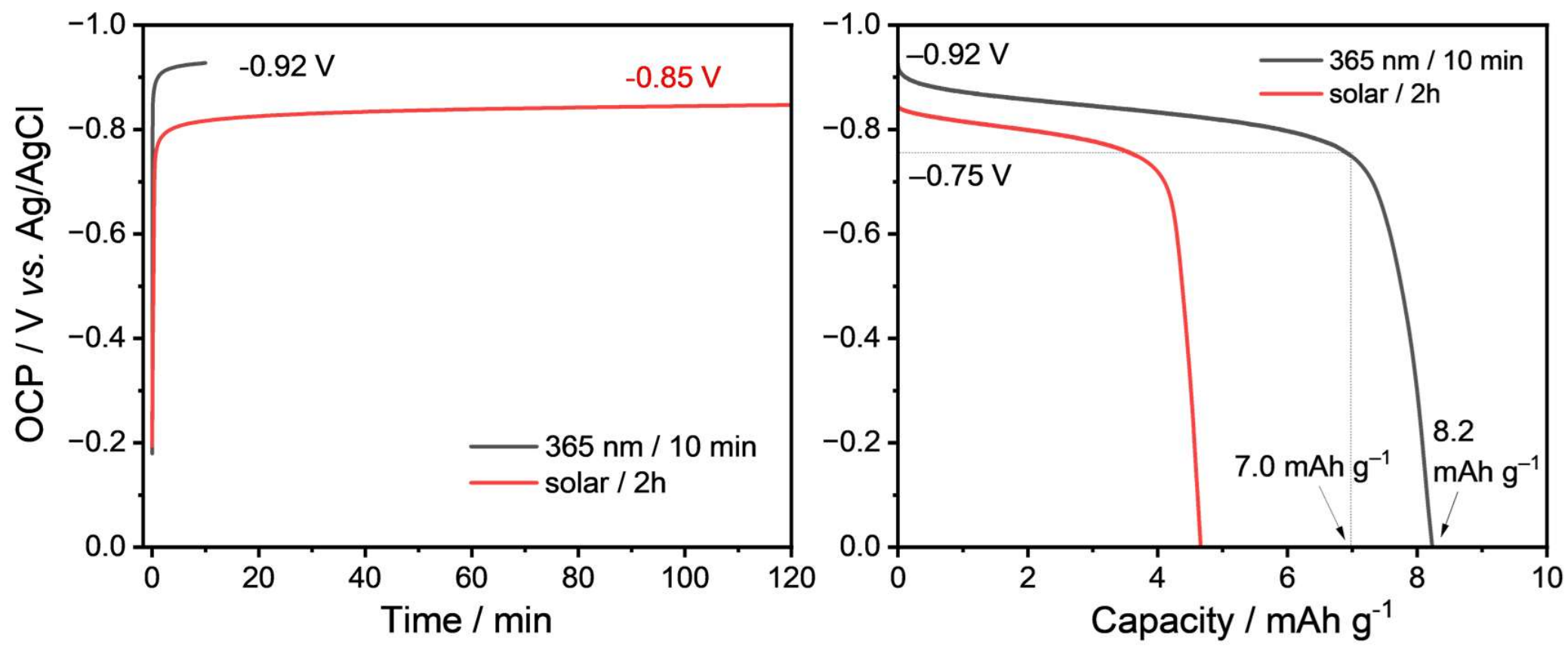

Figure S41. Charging of the CTF-Bpy photoanode in solar battery applications with 1 sun (AM 1.5 G, nominal power $P_{nominal} \approx$ 100 mW cm^{-2}, 2 h) or 365 nm UV ($P_{nominal}$ = 237 mW cm^{-2}, 10 min) illumination under OCP conditions. Subsequent electric discharge in the dark was performed at a fixed current density of 1.5 mA g^{-1} (normalized against mass of CTF-Bpy). Measurements were performed in 20 mM 4-MBA aqueous electrolyte under continuous Ar purging.

It can be seen from **Figure S41b** that the the stored electrons with potentials within −0.92 ~ −0.75 V account for 85.4 % of the total capacity (7.0 mAh g^{-1} of 8.2 mAh g^{-1}), suggesting the capacity is contributed mostly by the majority of the stored electrons. Similarly, we presume the H_2 evolution in the dark is driven by the majority of the stored electrons.

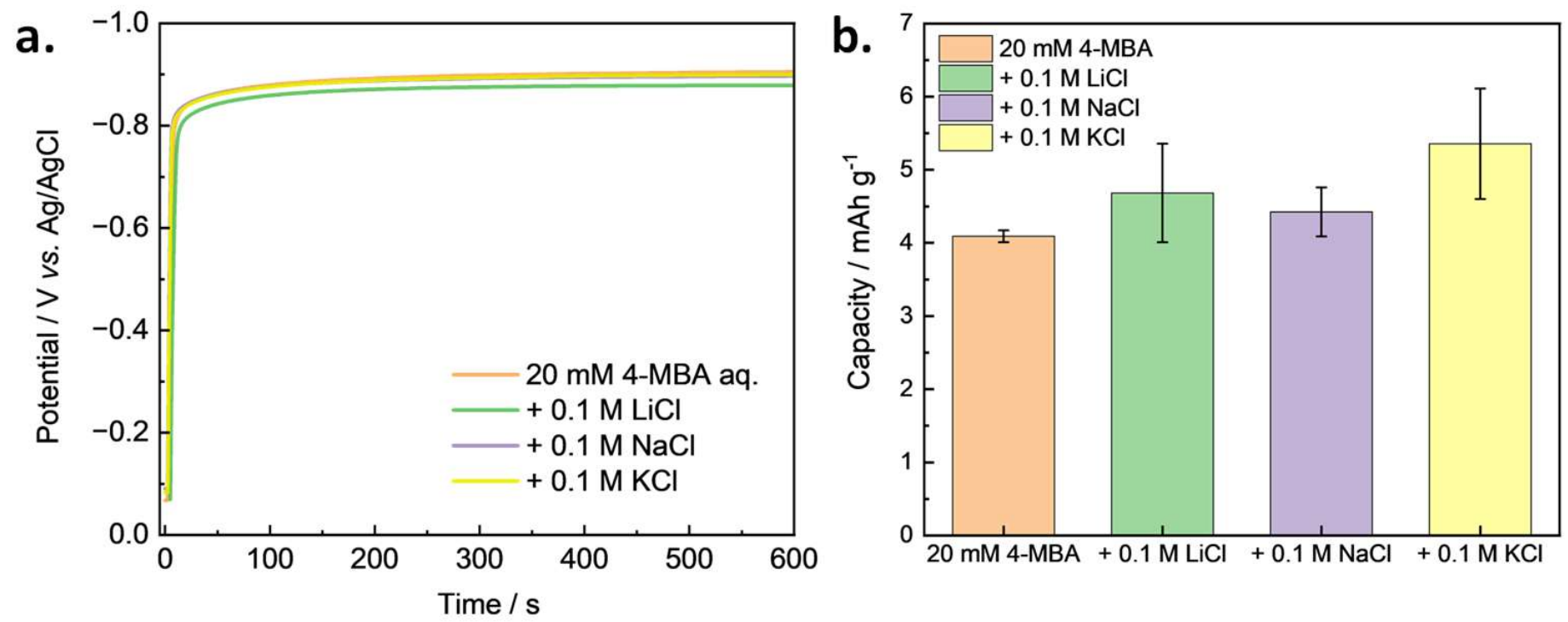

Figure S42. (a) Photocharging of the CTF-Bpy photoanodes in 20 mM 4-MBA aqueous solution in the absence and presence of 0.1 M LiCl, NaCl and KCl. (c) Discharge capacity of the CTF-Bpy photoanodes as a function of the electrolyte cation. The electrical discharge measurements were performed at a current density of 1.5 mA g^{-1}.

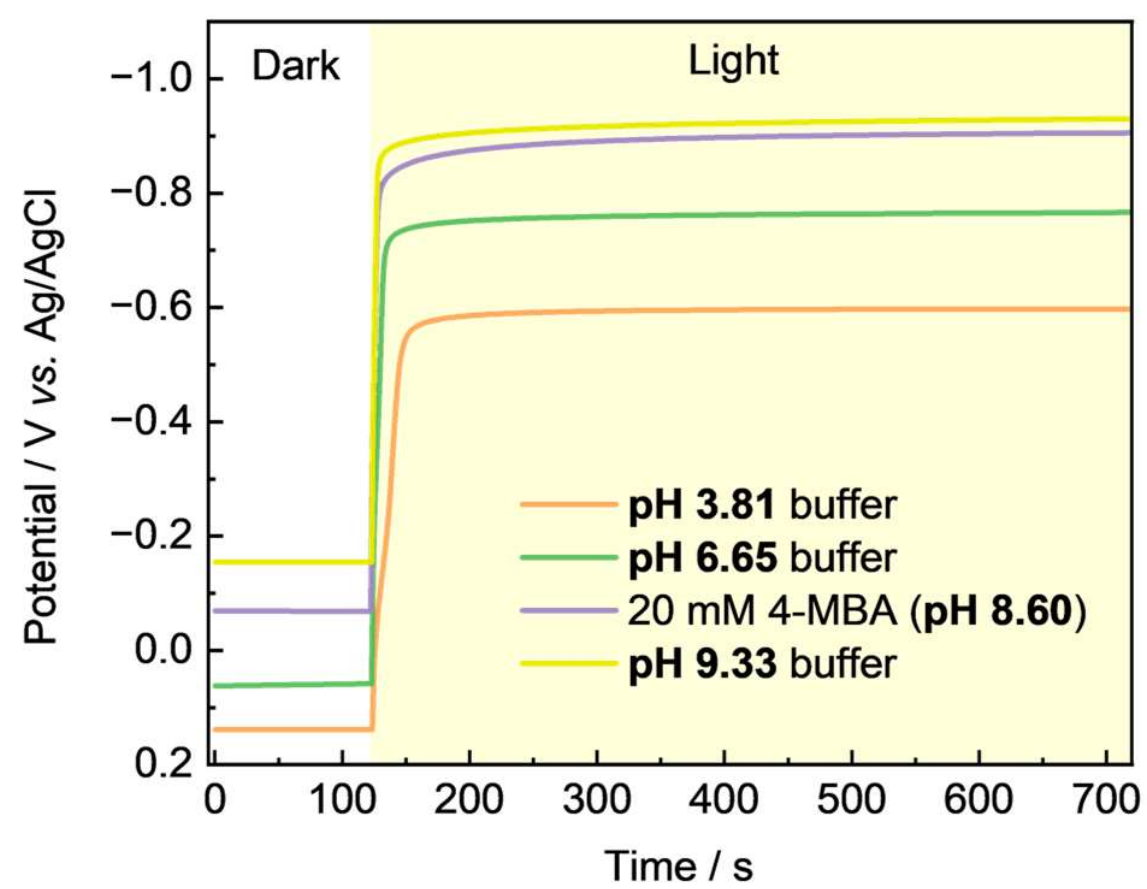

Figure S43. Photocharging of CTF-Bpy photoanodes in a 20 mM 4-MBA aqueous solution and in aqueous buffer solutions containing 20 mM 4-MBA at pH 3.81, 6.65 and 9.33. Photocharging was performed with 365 nm UV light for 10 min under continuous Ar flow. Before photocharging, the CTF-Bpy photoanodes were stabilized in each solution until a stable open circuit potential (OCP) was reached.

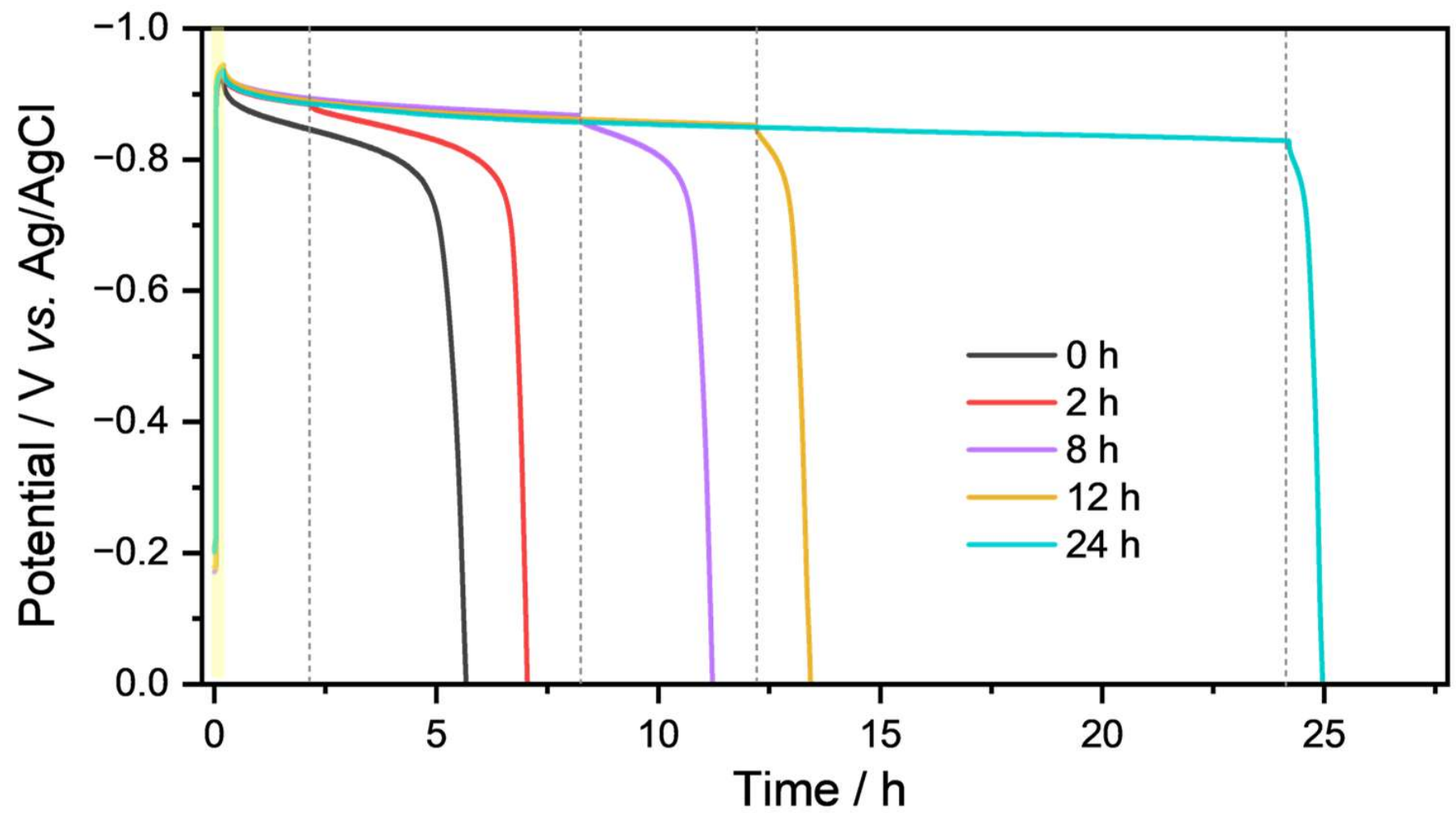

Figure S44. Photocharging of CTF-Bpy photoanode for 10 min (365 nm), followed by immediate (0 h) and delayed (2, 8, 12 and 24 h) electric discharge in the dark at a current density of 1.5 mA g^{-1}. Measurements were performed in 20 mM 4-MBA aqueous electrolyte under continuous Ar purging.

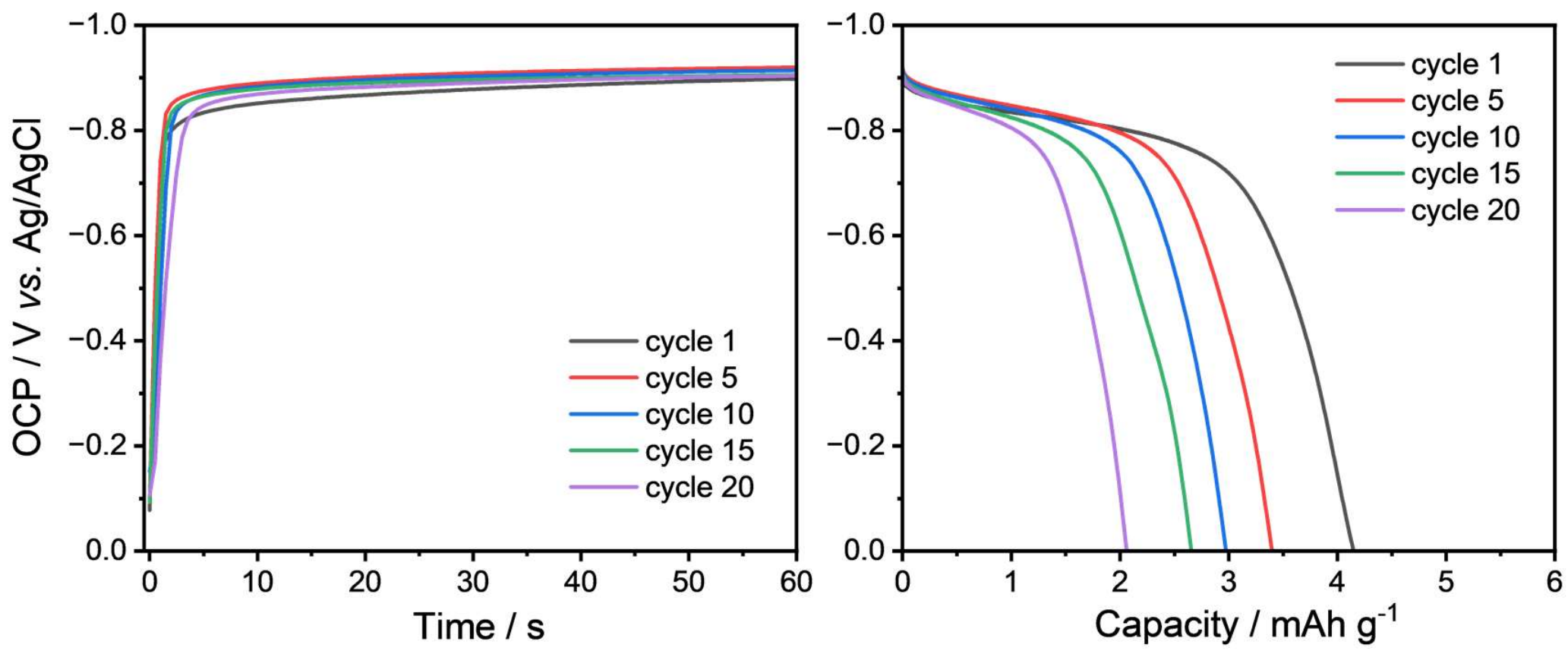

Figure S45. Representative profiles of photocharging (365 nm / 1 min, left) and subsequent immediate electric discharge (right) at a fixed current density of 10 mA g^{-1} of CTF-Bpy photoanode during cycling stability tests. Measurements were performed in 20 mM 4-MBA aqueous electrolyte under continuous Ar purging.

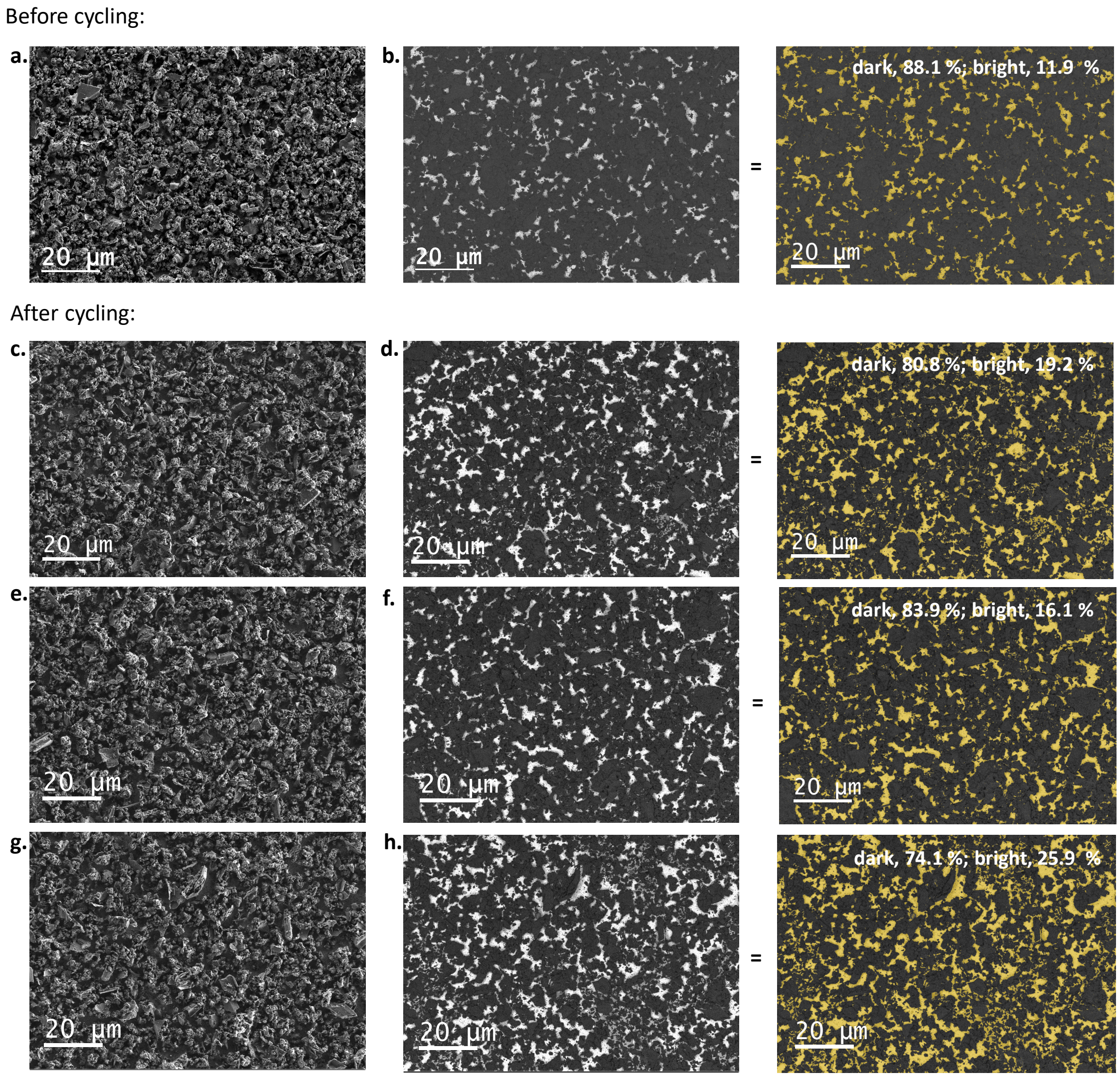

Figure S46. SEM images of drop-cast CTF-Bpy films on an FTO-coated glass substrate before (a, b) and after (c-h) the cycling study in Figure 5d. Images in (a), (c), (e) and (g) were recorded using the SE (secondary electron) detector, whereas images in (b), (d), (f) and (h) were acquired using the EsB (Energy and angle selective backscattered electron) detector at identical locations as above to enhance material contrast. Figure c/d, e/f and g/h were taken at different locations in the same film. In the EsB images, dark and bright regions correspond to CTF-Bpy covered and uncovered parts on FTO, respectively. The right-hand panels show the corresponding ImageJ-processed images of (b), (d), (f) and (h), in which the uncovered areas of FTO are highlighted in yellow (= bright) and their area fractions are indicated. The uncovered area on FTO increased from 11.9 % before cycling to an average of 20.4 % after cycling study, indicating partial loss of CTF-Bpy on FTO, which likely contributed to the capacity decay observed during the cycling experiment.

8. Dark photocatalysis

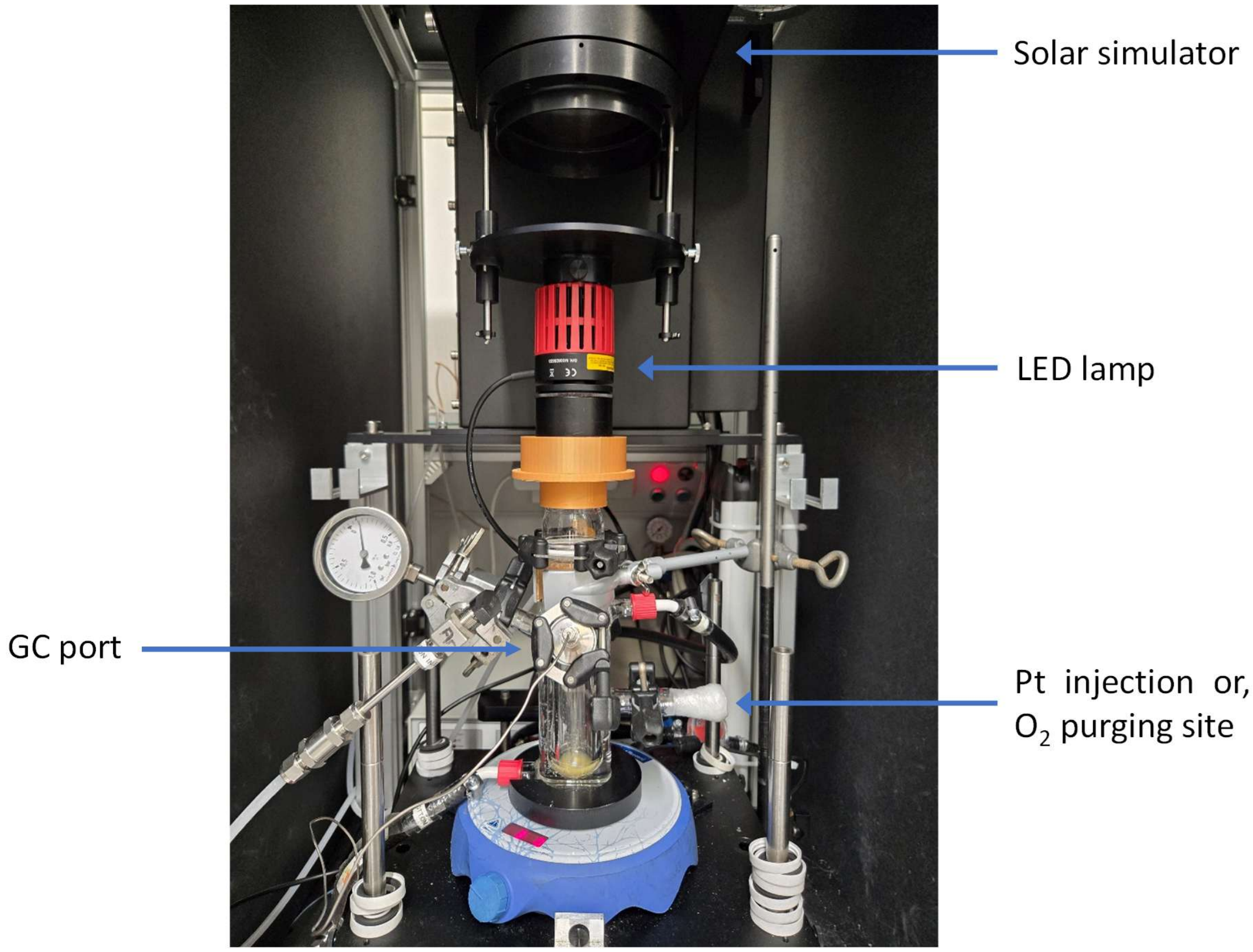

Figure S47. Experimental setup for the dark H_2O_2 production and dark H_2 evolution measurements. For the dark H_2O_2 production experiments, the GC sampling port is sealed.

a.

Sample preparation	Light	Dark
H_2O_2 / µmol g^{-1}	108	22

b.

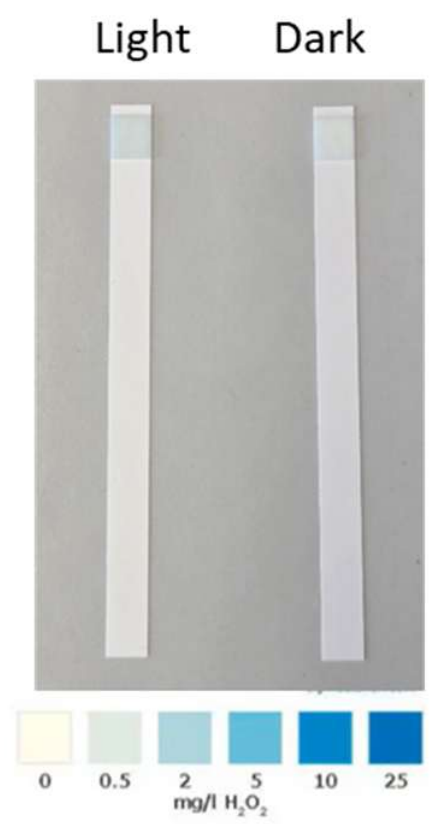

Figure S48. (a) Comparison of the amount of H_2O_2 in the suspension of CTF-Bpy in presence of 20 mM 4-MBA before illumination, with the suspension prepared under ambient light and O_2 (in air) (Light) and in the dark under Ar (Dark). The quantification of H_2O_2 was done through a spectrophotometric method based on the triiodide procedure.[4, 5] (b) H_2O_2 test strips are used as complementary method to quantify the amount of H_2O_2 in each suspension.

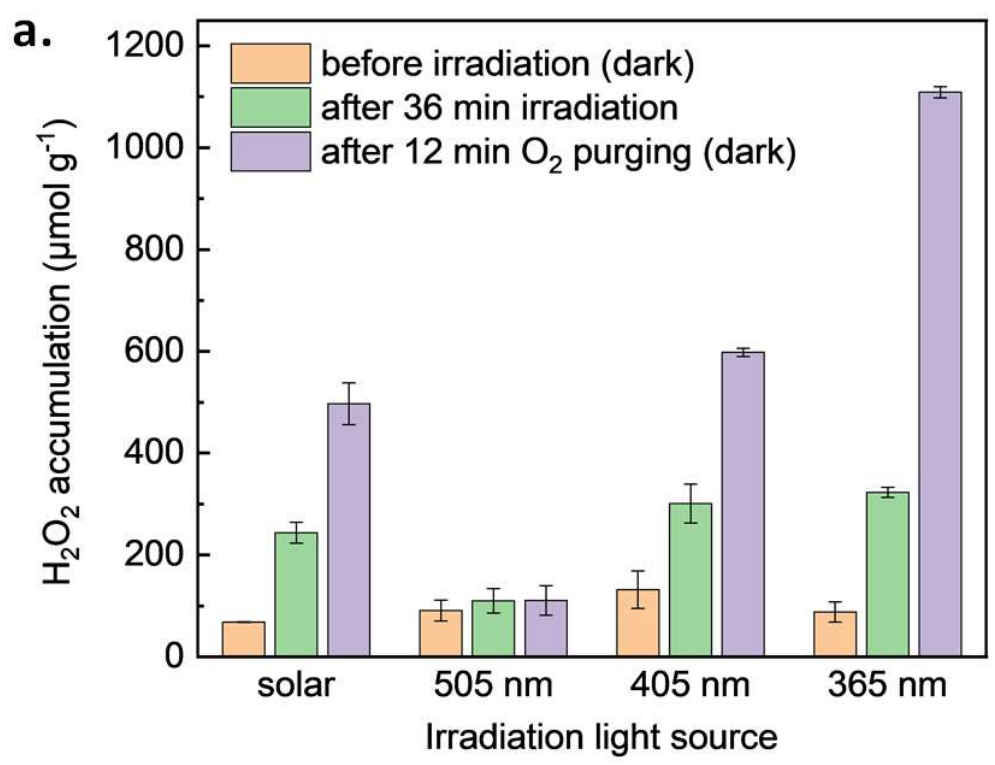

H_2O_2 / μmol g^{-1}	Solar	505 nm	405 nm	365 nm
before irradiation	68 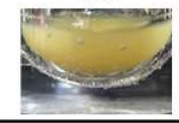	91 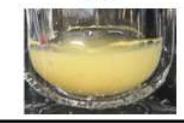	132 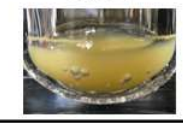	88 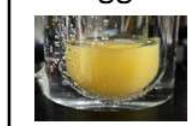
after irradiation	244 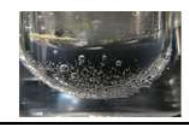	110 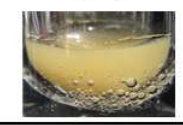	301 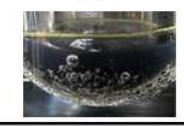	323 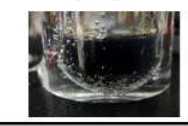
after O_2 purging	497 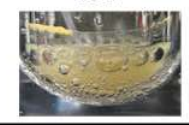	111 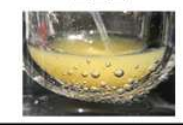	598 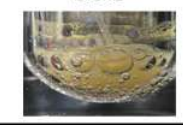	1109 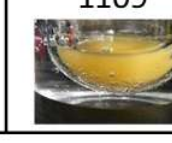

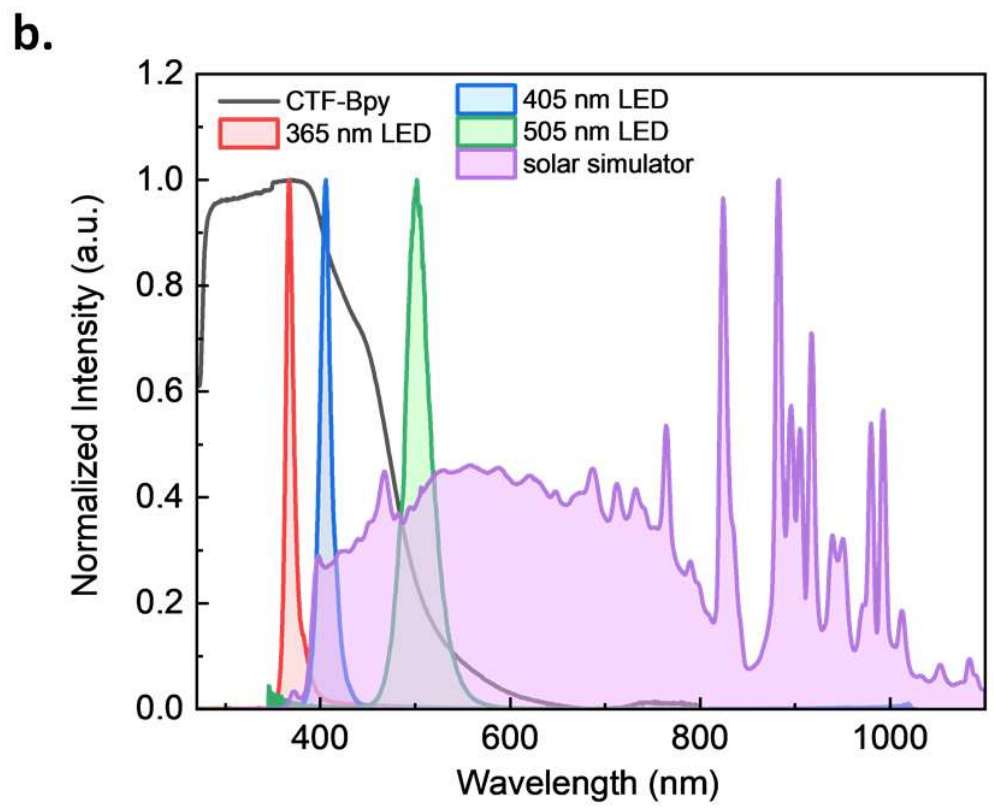

Irradiation source	365 nm	405 nm	505 nm	solar simulator
absolute area/emission	14.2	16.9	34.8	228.5
overlapped area	13.6	16.2	16.1	48.2
max. height within overlap	0.998	0.894	0.361	0.449
ratio of overlapped area	95.6 %	96.2 %	46.4 %	21.1 %

Figure S49. (a) Dark H_2O_2 production of CTF-Bpy after 36 min of irradiation using LEDs with different wavelengths (365, 405 and 505 nm) and solar simulator. Error bars represent the deviation between two independent experiments. The table on the right summarized the measured H_2O_2 concentrations in the suspensions before irradiation, after irradiation, and after O_2 exposure, together with photographs of the reaction suspension at each stage. (b) Spectral overlap of the LEDs (365, 405 and 505 nm) and solar simulator with the UV-vis spectrum of CTF-Bpy aqueous suspension containing 20 mM 4-MBA. The table on the right summaried the details of the spectral overlaps shown in Figure (b). The 365 nm, 405 nm, 505 nm LEDs and the solar simulator have output powers of 237, 131, 20 and 328 mW cm^{-2}, respectively. O_2 purging was performed for 12 min at a flow rate of 100 mL min^{-1}. Based on the results shown in Figure 6a of the main text, 12 min of O_2 purging is sufficient to completely discharge the stored electrons in CTF-Bpy. The slight variations in the H_2O_2 production measured after irradiation arise because these measurements were conducted under ambient air, and the results are sensitive to the duration of air exposure prior to analysis.

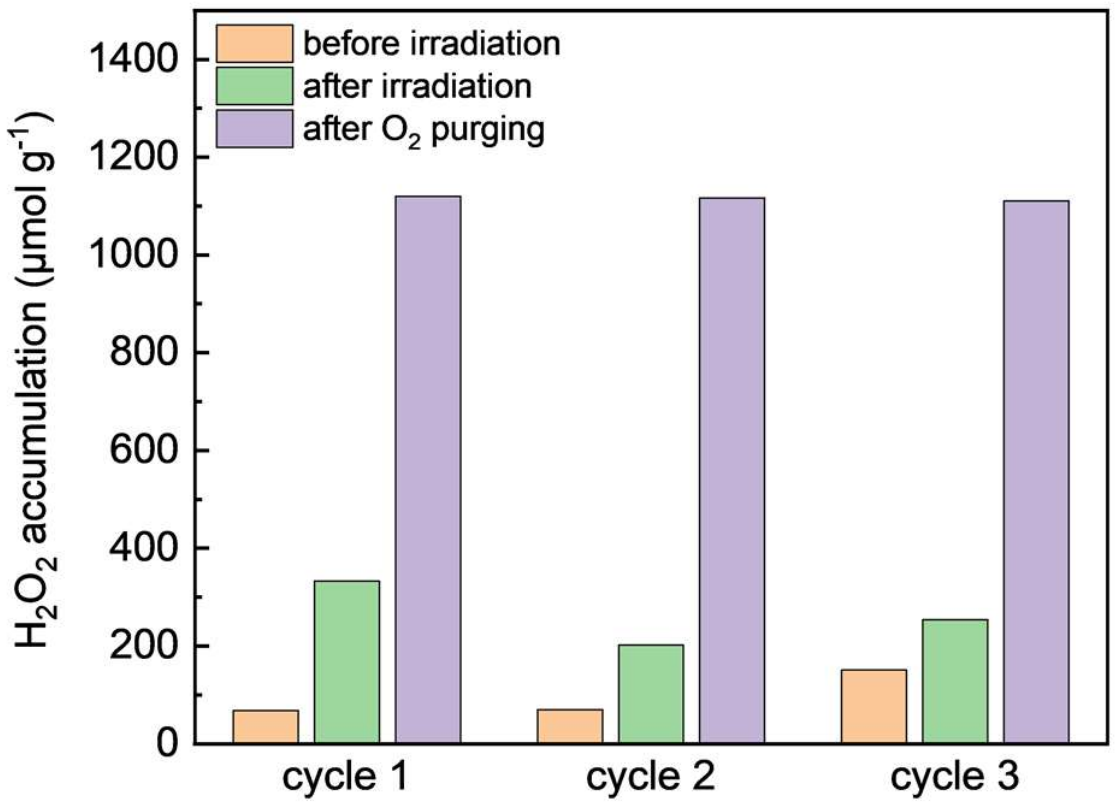

H_2O_2 / μmol g^{-1}	cycle 1	cycle 2	cycle 3
before irradiation	68 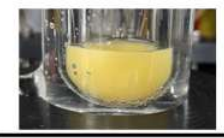	70 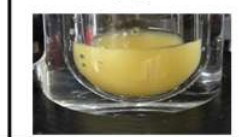	151 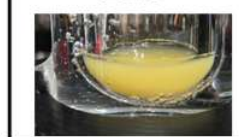
after irradiation	333 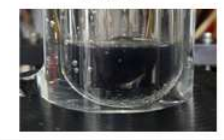	202 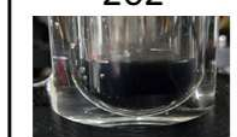	254 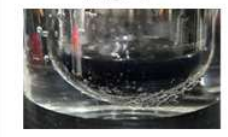
after O_2 purging	1120 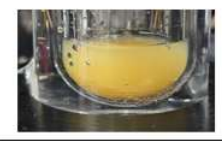	1117 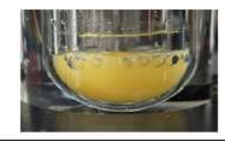	1111 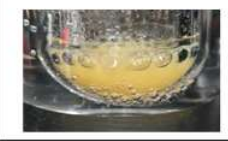

Figure S50. Cycling stability of CTF-Bpy for dark H_2O_2 production (left) and summary of the measured H_2O_2 concentrations in the suspension before irradiation, after irradiation, and after O_2 exposure, together with photographs of the reaction suspension at each stage (right). Photocharging was performed by irradiating an Ar-purged aqueous suspension of CTF-Bpy containing 20 mM 4-MBA with 365 nm UV light for 36 min. O_2 purging was peformed for 12 min at a flow rate of 100 mL min^{-1}. Based on the results shown in Figure 6a of the main text, 12 min of O_2 purging is sufficient to completely discharge the stored electrons in CTF-Bpy. After each cycle, CTF-Bpy was recovered by filtration, thoroughly washed with water, and dried under vacuum at 100 °C for 12 h before reuse. The slight variations in the H_2O_2 production measured after irradiation arises because these measurements were conducted under ambient air, and the results are sensitive to the duration of air exposure prior to analysis.

(1) Stepwise 1e⁻ pathway

$$O_2 + e^- \rightarrow O_2^{\bullet -}$$
$$O_2^{\bullet -} + e^- + 2H^+ \rightarrow H_2O_2$$

(2) Direct 2e⁻ pathway

$$O_2 + 2H^+ + 2e^- \rightarrow H_2O_2$$

Figure S51. The stepwise $1e^-$ and concerted $2e^-$ pathway of O_2 reduction to H_2O_2.

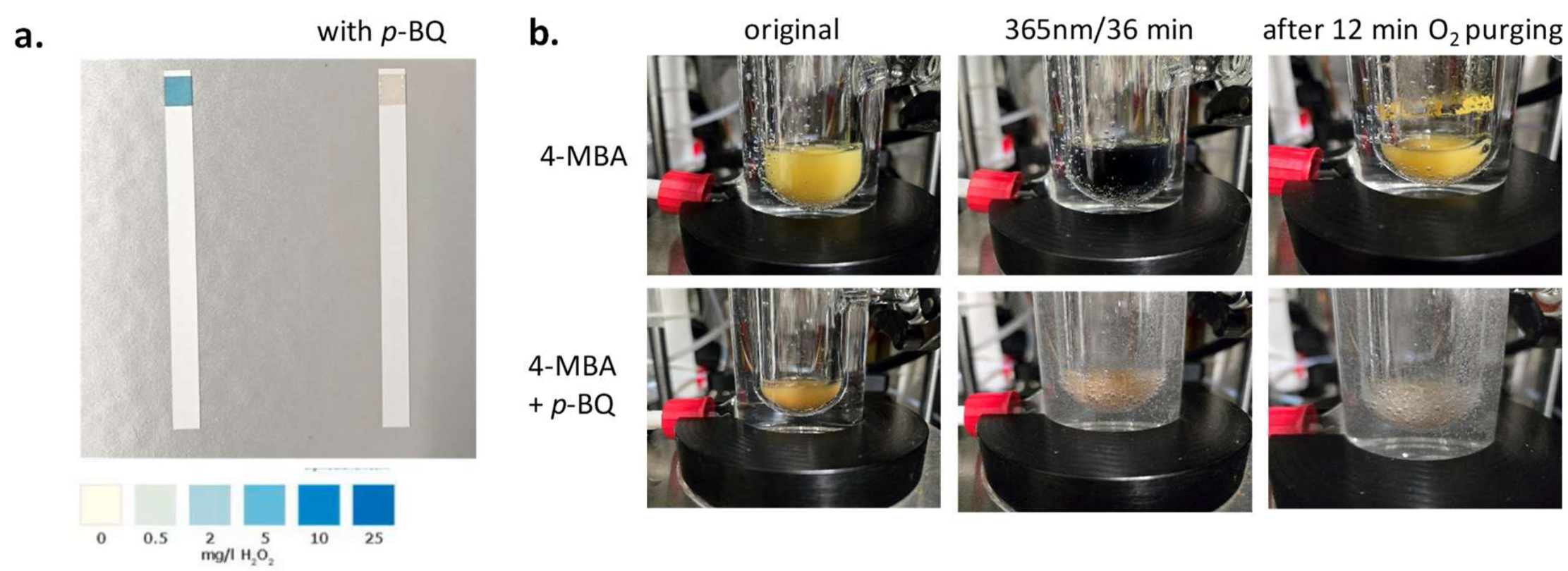

Figure S52. (a) Qualitative evaluation of H_2O_2 formation without (left) and with the presence of 10 mM *p*-benzoquinone (*p*-BQ) (right) after 36 min illumination (365 nm) and subsequent 12 min O_2 purging (100 mL min^{-1}). Due to the interference of *p*-BQ with the potassium iodide titrimetric assay, the relative peroxide levels were estimated using peroxide test strips.[15] (b) Photographs of the CTF-Bpy aqueous suspension without (top) and with the presence of 10 mM *p*-BQ (bottom) before illumination, after 36 min illumination (365 nm) and after 12 min O_2 purging.

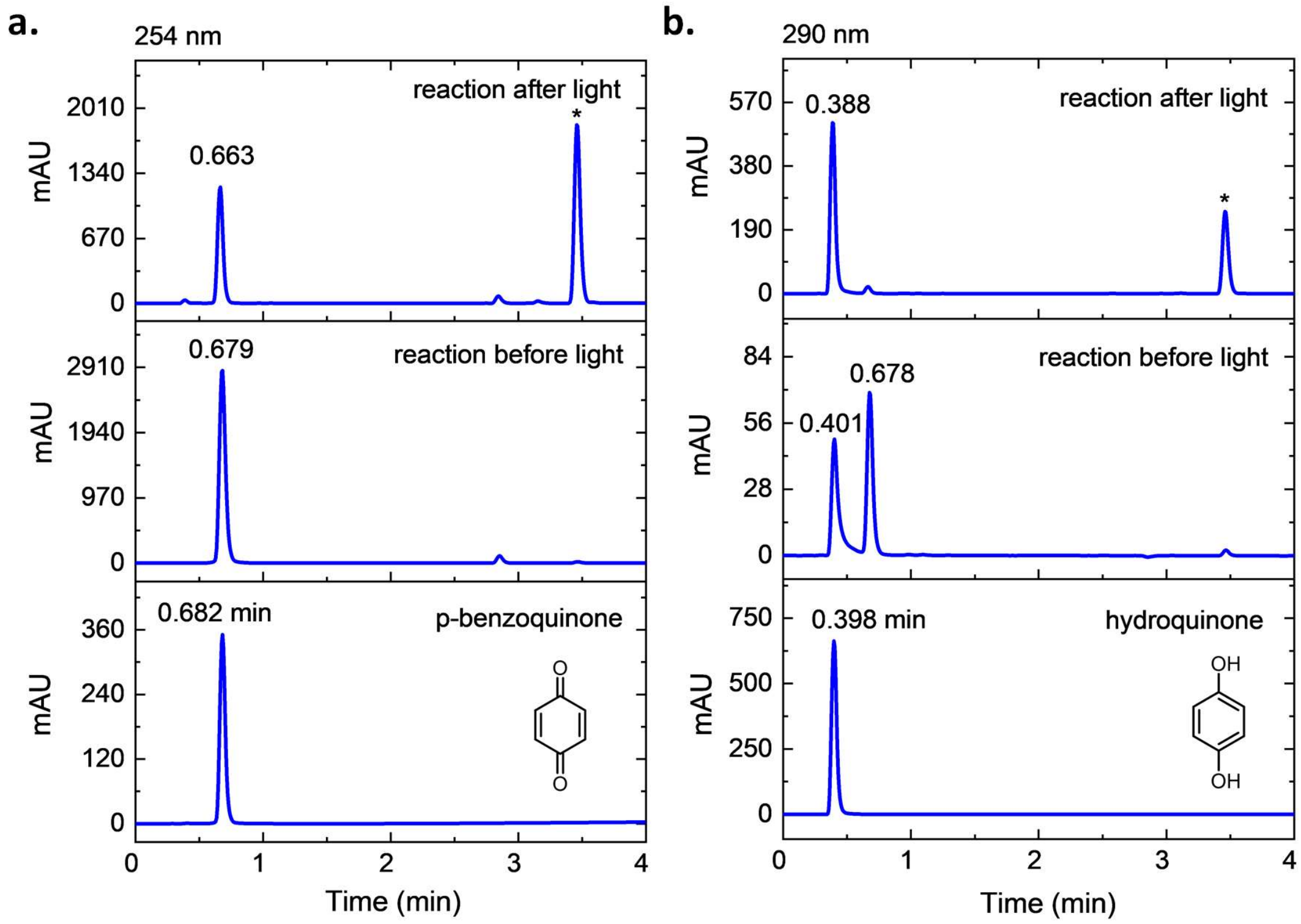

Figure S53. HPLC chromatograms of the reaction mixture containing CTF-Bpy in 20 mM 4-MBA aqueous solution with 10 mM *p*-benzoquinone (*p*-BQ), recorded before and after illumination, and compared with reference solutions of (a) *p*-BQ and (b) hydroquinone in water. Illumination of the reaction mixture was performed with an Ar-purged CTF-Bpy suspension (1 mg/mL) under 365 nm UV light for 10 min. HPLC analyses were performed using a C_{18} column at 40 °C with H_2O: acetonitrile (gradient program) with 0.1% formic acid addition as the eluent. The UV-vis spectrum of *p*-BQ shows a maximum absorption at ~ 250 nm, whereas hydroquinone exhibits maxima at ~ 220 nm and ~ 290 nm.[16] The results show that *p*-BQ suppresses the photocharging of CTF-Bpy by scavenging the photogenerated electrons and undergoing self-reduction to hydroquinone. *Asterisks denotes *p*-tolualdehyde, which is the oxidation product of 4-MBA.

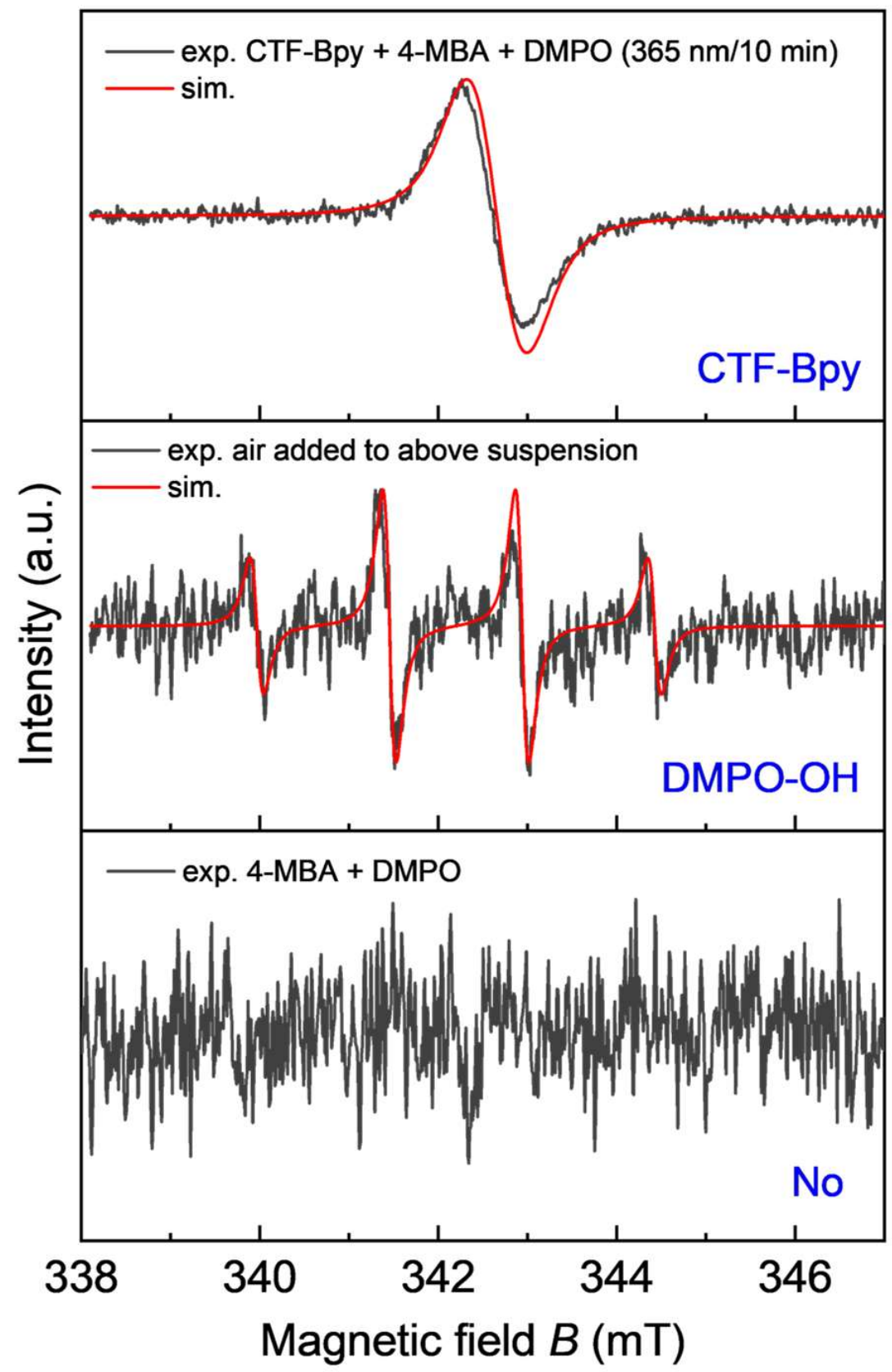

Figure S54. Experimental (black) and simulated (red) EPR spectra of the CTF-Bpy aqueous suspension (10 mg/mL) containing 20 mM 4-MBA and 100 mM 5,5-dimethyl-1-pyrroline N-oxide (DMPO) after 10 min of 365 nm UV irradiation (top), and after subsequent air introduction, showing the characteristic DMPO–•OH adduct spectrum (middle). A control EPR spectrum of an aqueous solution containing 20 mM 4-MBA and 100 mM DMPO in air is shown at the bottom. Simulation Parameters for CTF-Bpy radical: S=1/2, *g*-value =2.0033, Gaussian-linewidth=0.45 mT, Lorentzian-linewidth = 0.37 mT. Simulation parameters for DMPO-•OH adduct: S=1/2, g=2.006, Coupling Nuclei: N,H; A(N)=41.8 MHz, A(H)=41.8 MHz, Gauss-linewidth=0 mT, Lorentz-linewidth = 0.15 mT.

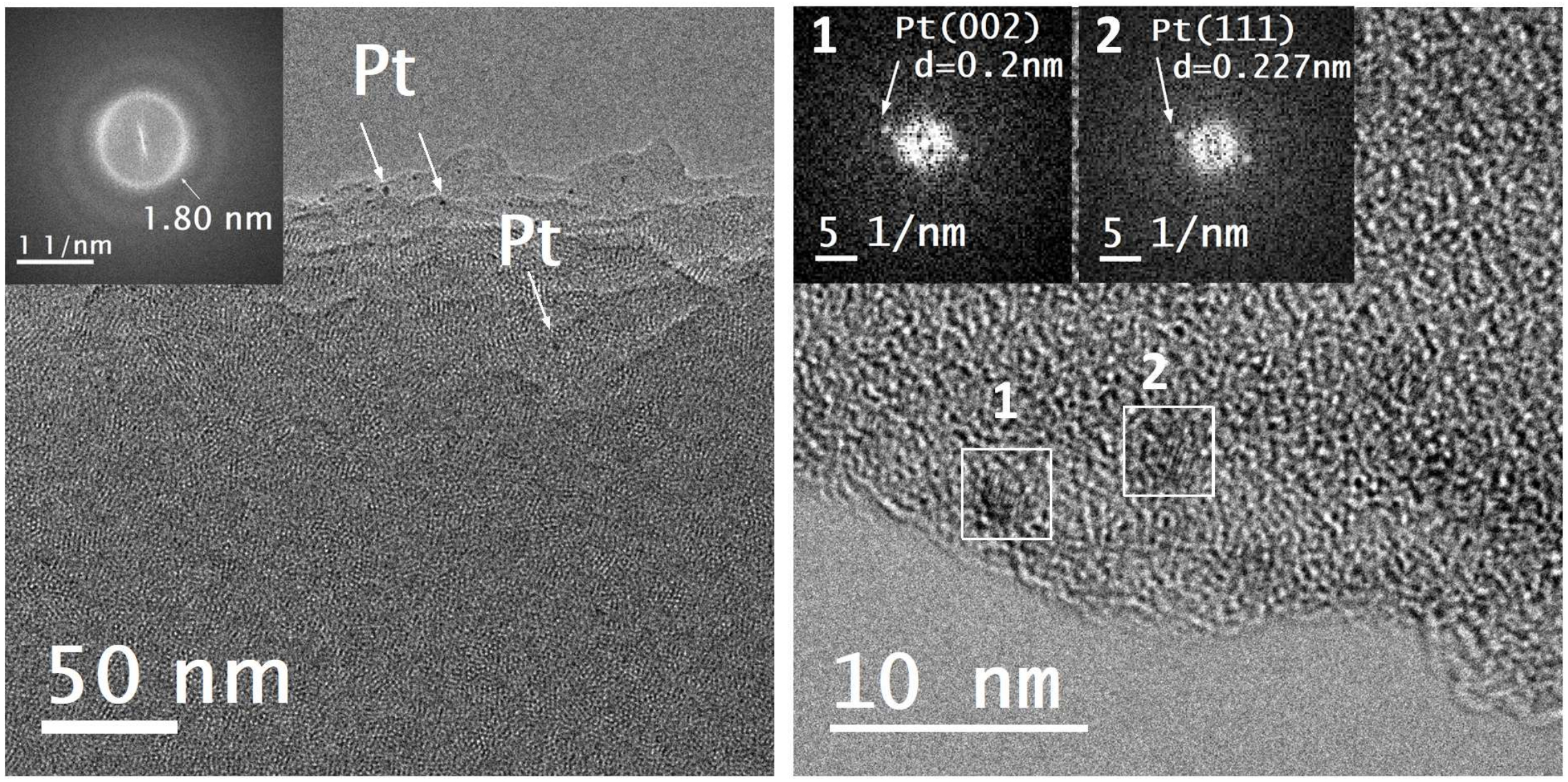

Figure S55. TEM images of CTF-Bpy after dark H_2 evolution, when $H_2PtCl_6.6H_2O$ was used as a co-catalyst and the sample was illuminated with 365 nm UV light for 36 min. Inset are the FFT (Fast Fourier Transform) of the image on the left and the selected area (white square) of the image on the right. This result confirms the *in situ* formation of Pt nanoparticles from the reduction of $H_2PtCl_6{\cdot}6H_2O$ in the dark, where CTF-Bpy acts as a reductant.

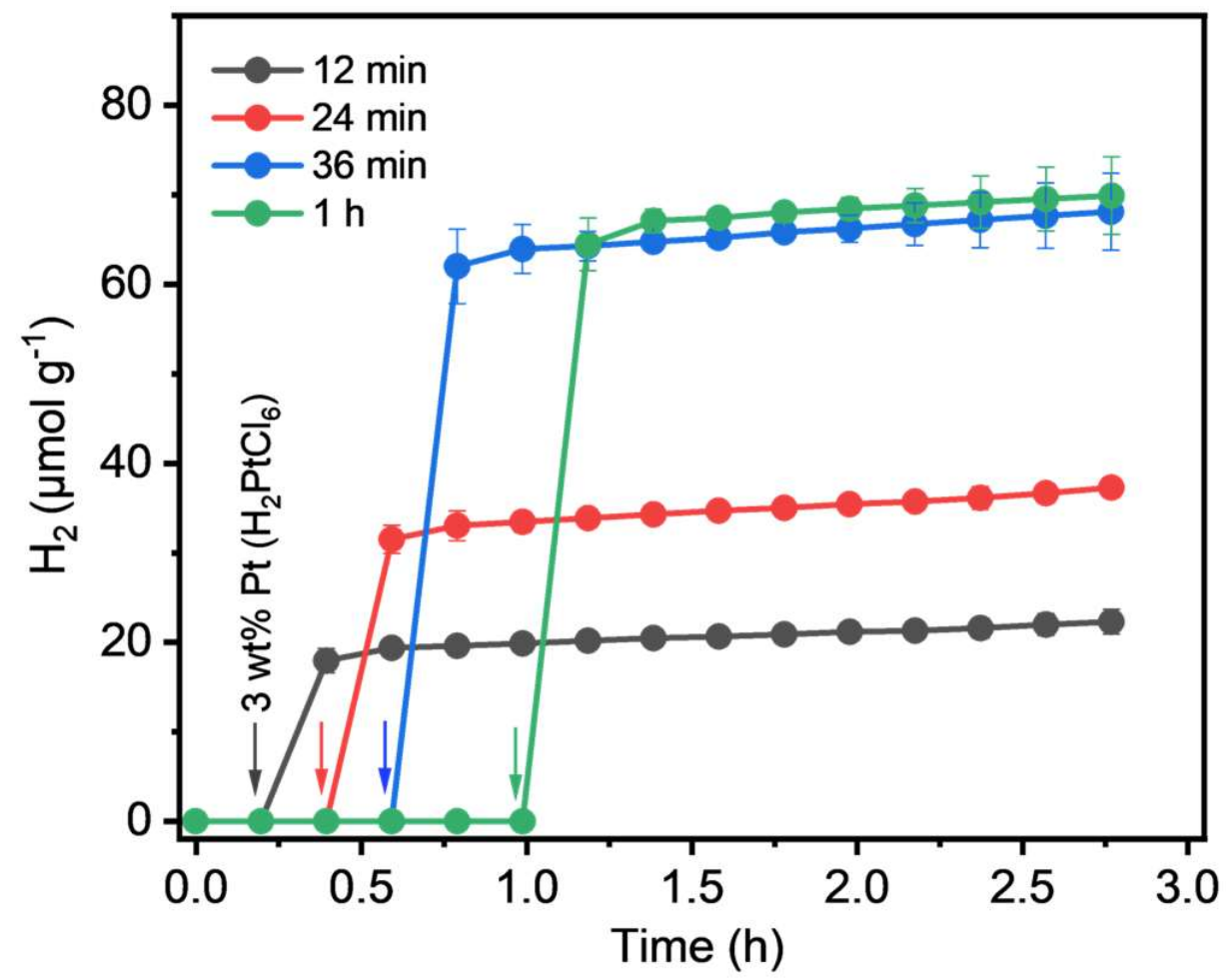

Figure S56. Dark H_2 evolution of CTF-Bpy with various illumination time (12 min, 24 min, 36 min and 1 h). Experiments were performed with 365 nm UV illumination in 20 mM 4-MBA aqueous suspension. 3 wt% $H_2PtCl_6 \cdot 6H_2O$ was injected immediately after switching off the illumination. This result suggests that optimum H_2 evolution is achieved at 36 min of illumination, without significant changes by extending the illumination time to 1 h. Error bars represent the means and standard deviations from two independent experiments using two different batches of CTF-Bpy.

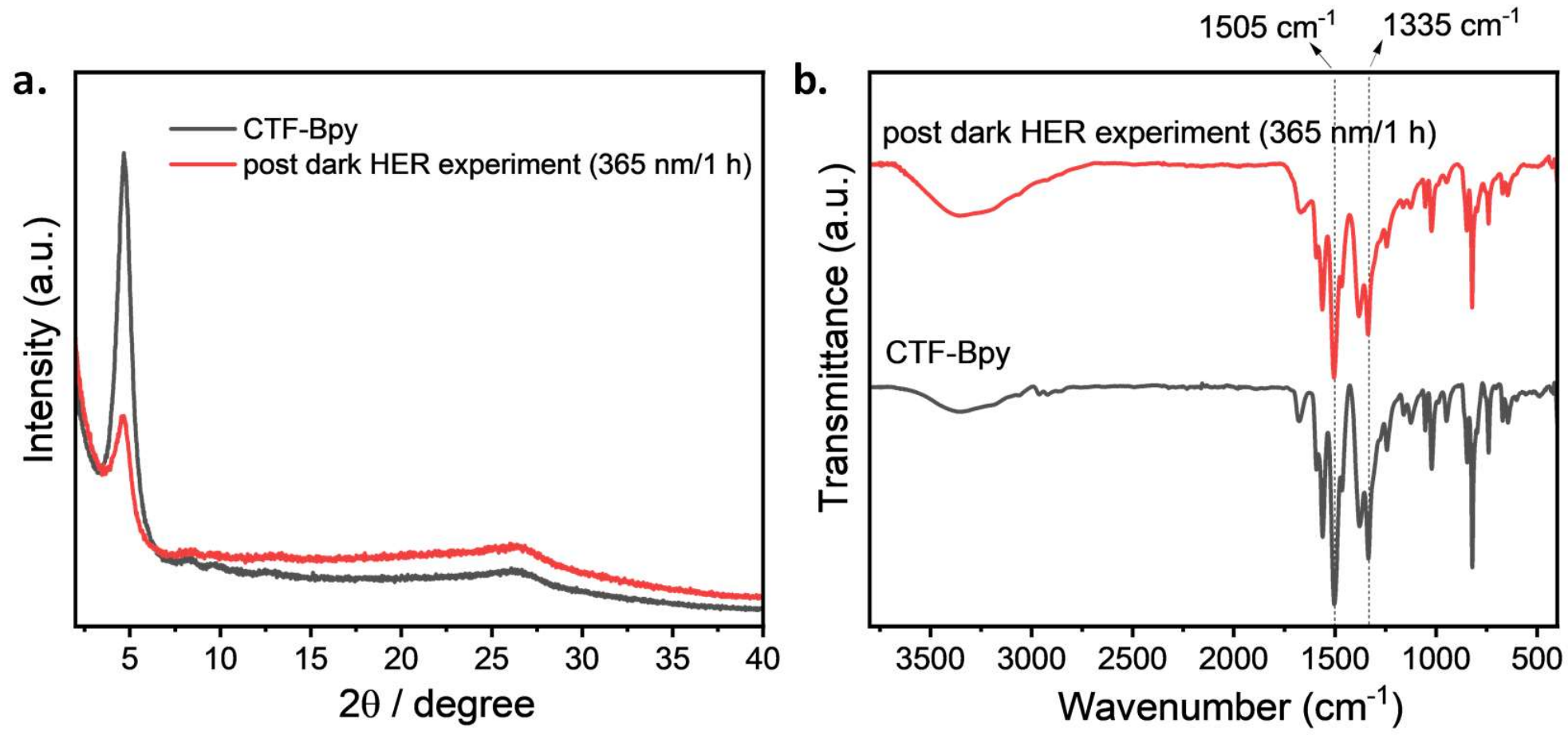

Figure S57. Comparison of (a) PXRD and (b) FTIR of CTF-Bpy before and after dark H_2 evolution experiments (1 h 365 nm UV illumination). The characteristic vibrations of triazine unit at 1505 and 1335 cm^{-1} in the FTIR spectra are indicated. After the dark photocatalysis experiment, the powder sample was filtered, washed with DMF and dried at 120 °C in vacuum oven for 8 h before doing the PXRD and FTIR measurements.

Despite a noticeable loss in crystallinity, FTIR confirmed the retention of triazine linkage of CTF-Bpy after 1 h 365 nm UV illumination and post dark H_2 evolution experiment.

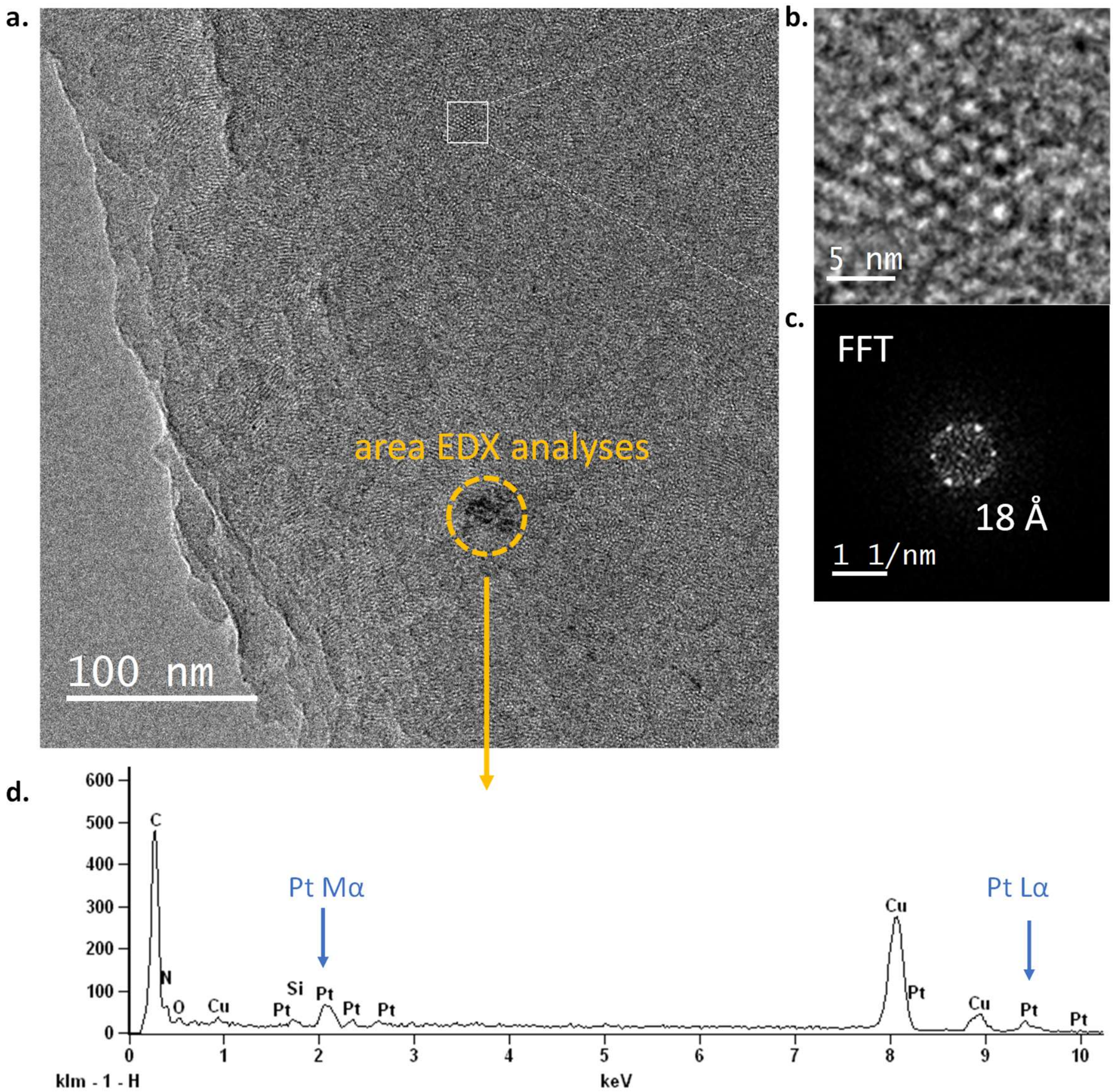

Figure S58. (a) TEM image of CTF-Bpy after a dark H_2 evolution experiment. (b) Enlarged image of the selected area in (a) (white square) showing the pore structures and (c) the corresponding FFT of the selected white square area; (d) Energy-dispersive X-ray (EDX) spectrum of the selected orange colored circle in (a). The presence of Cu is due to the Cu grid. Dark H_2 evolution was performed by illuminating the suspension for 1 h by 365 nm UV illumination, followed by the addition of 3 wt% Pt (with $H_2PtCl_6.6H_2O$) into the system in the dark. The CTF-Bpy used for analysis was collected by filtration, followed by water, DMF washing and then dried in 80 °C vacuum oven overnight.

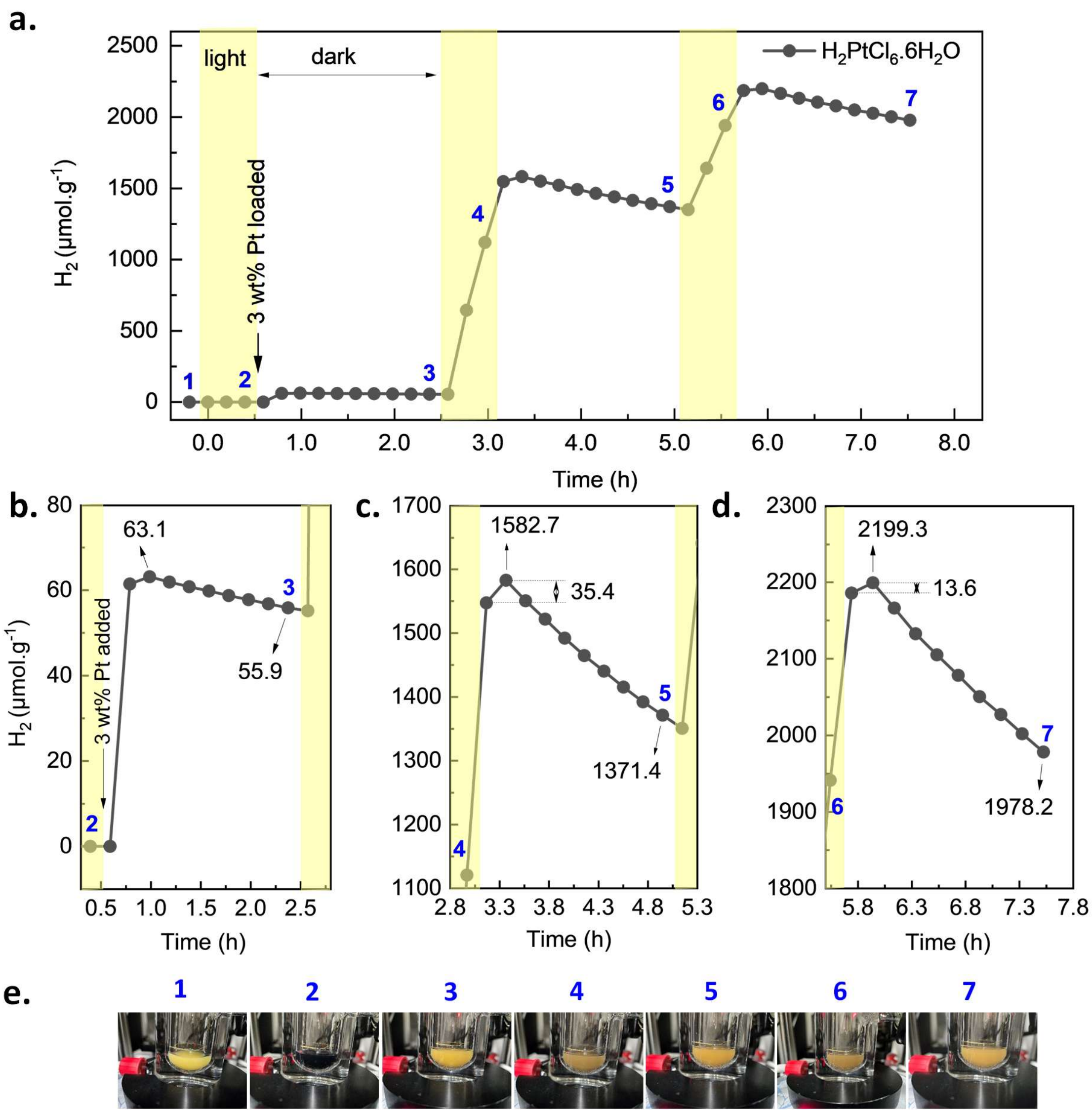

Figure S59. (a) Cycling study of dark H_2 evolution of CTF-Bpy using H_2PtCl_6 as the cocatalyst. Enlarged views of the (b) first, (c) second, and (d) third cycle of dark H_2 evolution shown in (a). (e) Photographs of the suspension at the indicated time points in (a), showing the corresponding color changes.

The decrease in the amount of H_2 measured during the dark period in the three cycles is due to our gas chromatography (GC) sampling procedure. Specifically, a fixed volume of gas is automatically withdrawn from the reactor for GC analysis during each measurement run, resulting in a corresponding decrease in the amount of H_2 remaining in the reactor. This contribution is supported by the consistent relative decrease in the measured H_2 amount across the three cycles: from 63.1 to 55.9 μmol g^{-1} in the first cycle, from 1582.7 to 1371.4 μmol g^{-1} in the second cycle, and from 2199.3 to 1978.2 μmol g^{-1} in the third cycle, corresponding to decreases by 11 %, 13 %, and 10 %, respectively. After each gas sample is withdrawn for GC analysis, the same volume of Ar is backfilled into the reactor to compensate for the resulting decrease in internal pressure.

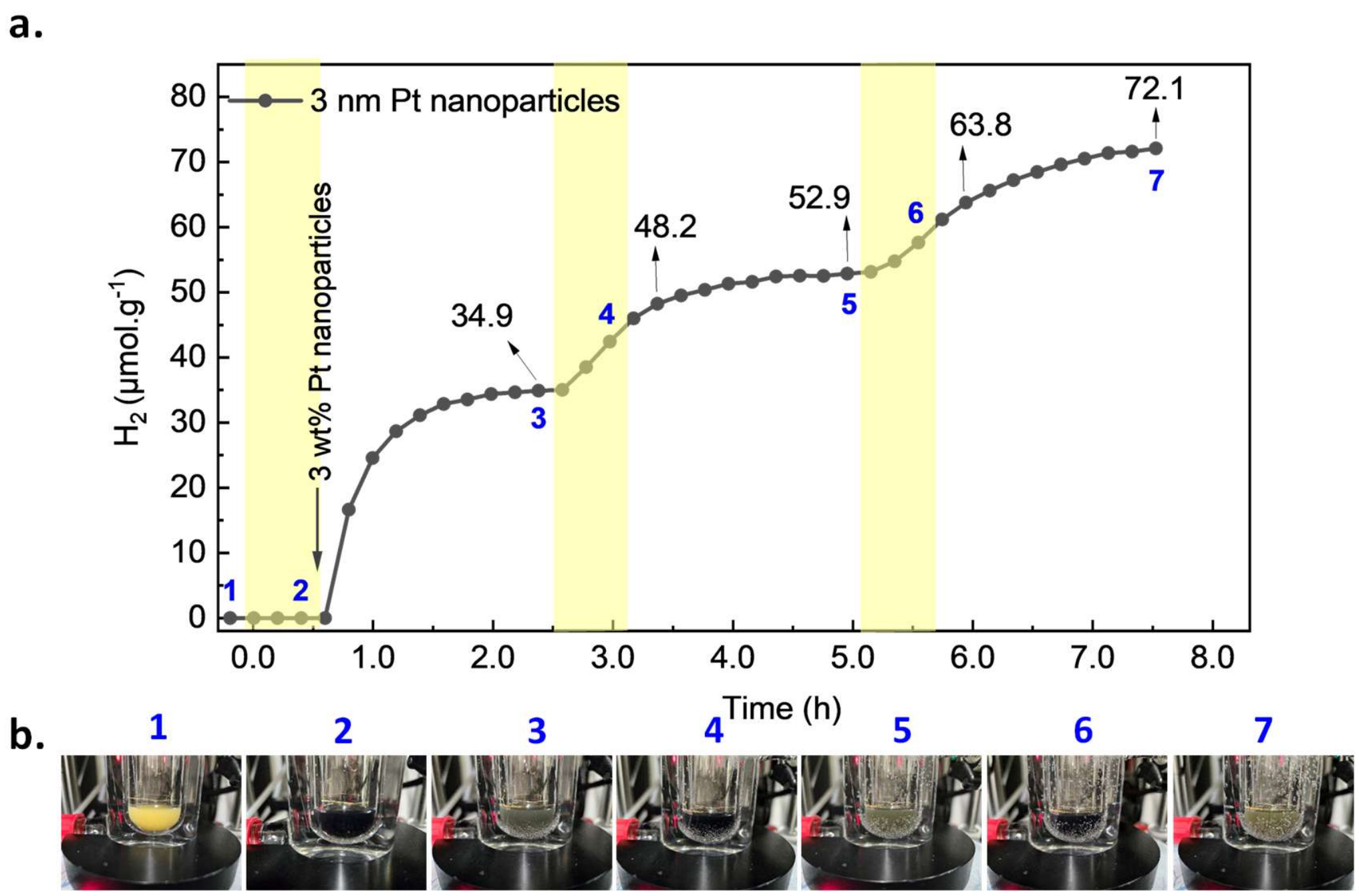

Figure S60. (a) Cycling study of dark H_2 evolution of CTF-Bpy using Pt nanoparticles (3 nm) as the cocatalyst. (b) Photographs of the suspension at the indicated time points in (a), showing the corresponding color changes. *Unlike the system using H_2PtCl_6, this system still exhibits the characteristic color change of the suspension to a dark color after illumination during the second and third cycles. Moreover, the amount of H_2 produced during the dark period continues to increase across all three cycles. Both observations can be attributed to the slower electron transfer from CTF-Bpy to Pt nanoparticles in this system.

Table S4. Comparison of the reported materials for dark H_2 evolution.

Photocatalysts		SED	solvent	Irradiation wavelength /time/power	Dark H_2 evolution		Photo-OCP/ reduction potential	Ref.
					max. H_2 /μmol g^{-1}	co-catalyst		
carbon nitrides	NCN-CN_x (K-PHI)	10 mM 4-MBA	phosphate buffer	AM 1.5/ 2 h/ 100 mW cm^{-2}	125	0.25 wt% Pt nano. (3 nm)	-0.5 V vs NHE	3
	CCN	20 v% MeOH	H_2O	300 W Xenon lamp (λ > 420 nm filter)/2 h/-	13 μmol h^{-1} (50 mg)	3 wt% Pt nano.	-0.6 V vs /	17
	H-PHI	15 mM 4-MBA	phosphate buffer	AM 1.5/2 h/ 100 mW cm^{-2}	27	0.33 wt% Pt nano. (3 nm)	/	18
	CN-500-4	10 v% benzyl alcohol	H_2O	300 W Xenon lamp (λ > 420 nm filter)/1 h/-	30 μmol h^{-1} (20 mg)	3 wt% Pt nano.	/	19
**Molecular POM**	PS-POM	sodium ascorbate	DMF	470 nm/ 15 min/ 40 mW cm^{-2}	e- to H_2 % = 41 %	/	-1.1 V vs Fc/Fc+	20
**MOFs**	MIL-125-NH_2	MeOH	MeOH	300 W Xe lamp (UV cut-off filter, 200–800 nm)/1 h/-	ca. 180	1.25 wt% Pt nano. (3 nm)	0.16 mV vs. Ag/AgCl	21
	ReCl-253 (MOF-253)	TEOA	H_2O/ MeCN	450 nm/90 min/-	72	$Co(dmgH)_2$(4-COOH-py)Cl (1.9 nmol mL^{-1})	/	22
	MnBr-253 (MOF-253)	TEA	H_2O/ MeCN	450 nm /1 min/ 20 mW cm^{-2}	ca. 180	$Co(dmgH)_2$(4-COOH-py)Cl (9.6 nmol mL^{-1})	/	23
	MIP-177(Ti)-LT	25 v% MeOH	H_2O	365 nm/ 30 min/ 200 mW cm^{-2}	ca. 300	1.13 wt% Pt nano. (3 nm)	/	24
	COK-47_{bipy}	4-MBA	H_2O	365 nm/	90	1 wt% Pt nano. (3 nm)	-0.9 V vs Ag/AgCl	25
**metal oxides**	$(TBA)^+$ $(NbWO_6)^-$	10 v% MeOH	H_2O	365 nm/ 30 min/ 206 mW cm^{-2}	7.16	1.05 wt% Pt nano. (3 nm)	-0.81 V vs Ag/AgCl	26
**COFs**	CTF-Bpy	20 mM 4-MBA	H_2O	365 nm/ 36 min/ 237 mW cm^{-2}	68.1	3wt% Pt (H_2PtCl_6)	-0.92 V vs Ag/AgCl	This work
					38.4	3wt% Pt nano.		

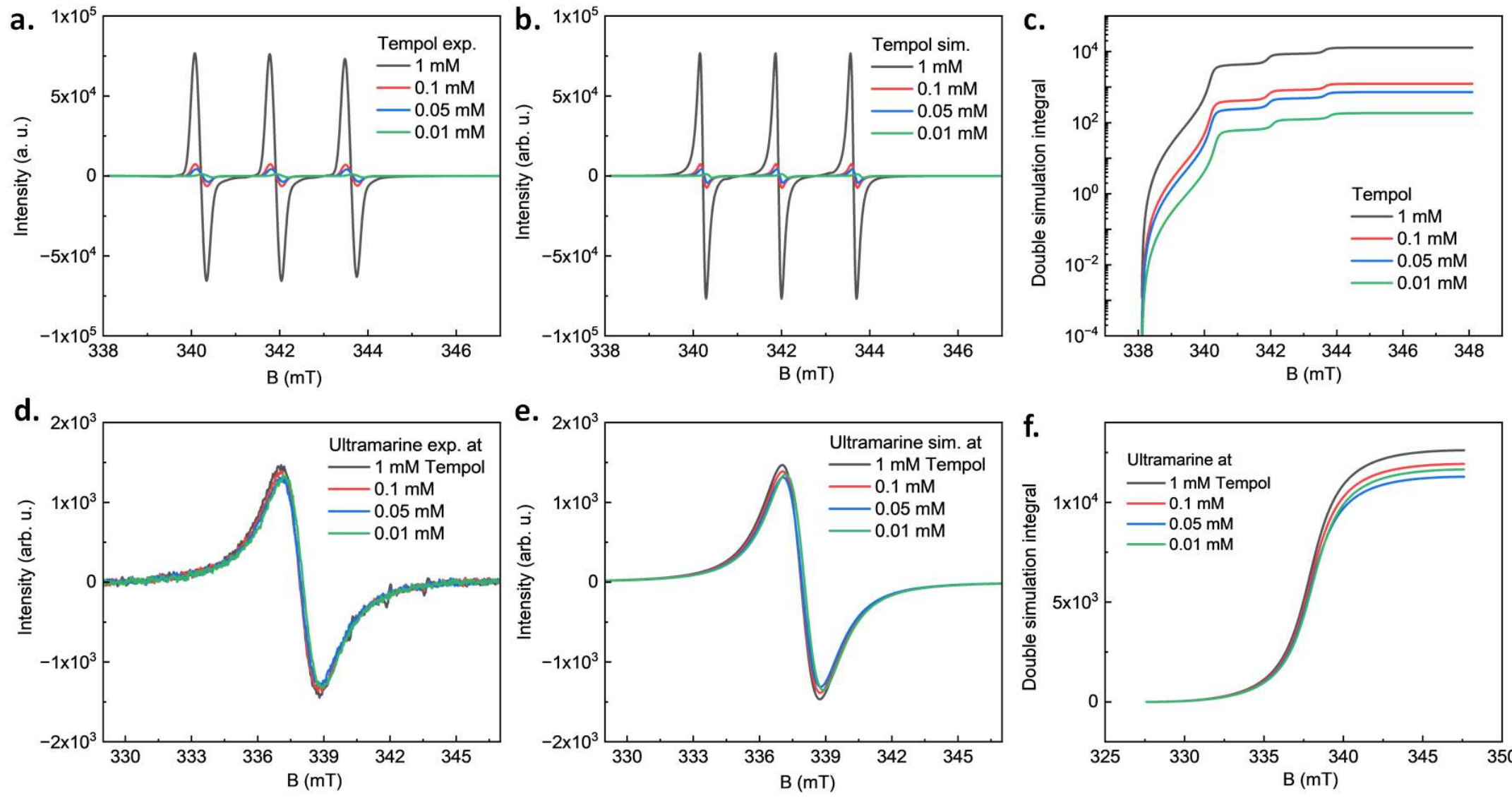

Figure S61. (a-c) Experimental EPR spectra, corresponding simulated spectra and double integration of the simulated spectra for aqueous 4-hydroxy-2,2,6,6,-tetramethyl-piperidine-1-oxyl (Tempol) solutions at different concentrations. (d-f) Experimental EPR spectra, simulated and double integration of the simulated spectra for the ultramarine standard sample. For each Tempol measurement shown in (a), the ultramarine standard in the second sample chamber of the resonator was measured under the same tuning conditions to account for changes in the Q-factor.

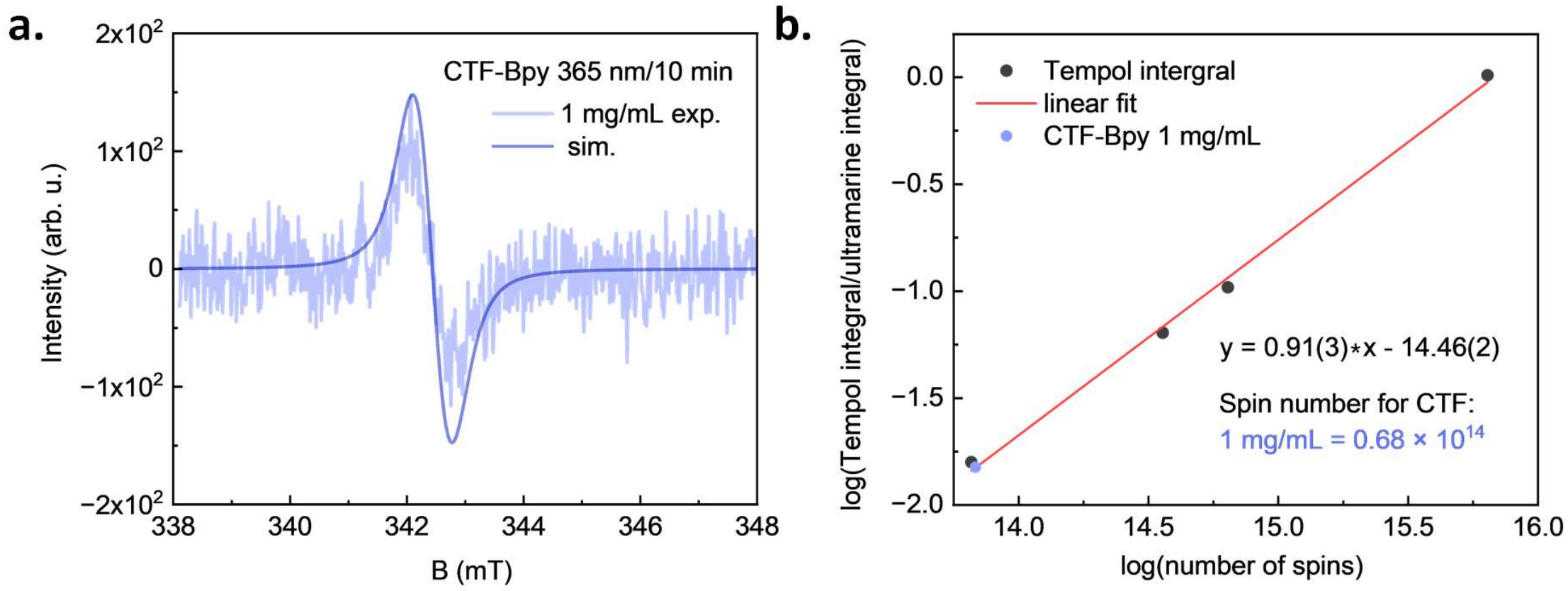

Figure S62. (a-c) (a) Experimental and corresponding simulated EPR spectra of photocharged CTF-Bpy aqueous suspension at a concentration of 1 mg/mL. Photocharging was performed with 365 nm UV irradiation for 10 min in the presence of 20 mM 4-MBA. Simulation parameters for the CTF-Bpy radical signal: g-value=2.0033, Gaussian-linewidth=0.45 mT, Lorentzian-linewidth = 0.37 mT. (b) Linear fit of the logarithm of the double integration of the simulated EPR signal of Tempol radical corrected by the ultramarine standard as a function of the number of spins (black points). The double integration of the simulated EPR signal of CTF-Bpy, also corrected by the ultramarine standard, at concentrations of 1 mg/mL, was fitted to the Tempol calibration curve to determine the spin concentrations.

Based on the fitting in (b), the number of spins for 1 mg/mL CTF-Bpy aqueous suspensions is determined to be 0.68×10^{14}. Using Avogadro's constant and the mass of CTF-Bpy used during the measurement (0.0159 mg for 1 mg/mL suspension), the spin concentration results in 0.072 mmol/g. The same experiments for the photocharged CTF-Bpy suspension were repeated two more times, resulting in a spin concentration of 0.213 and 0.013 mmol/g. The average spin concentration obtained from the three measurements (0.10 mmol/g) was used for comparison in **Table 5**.

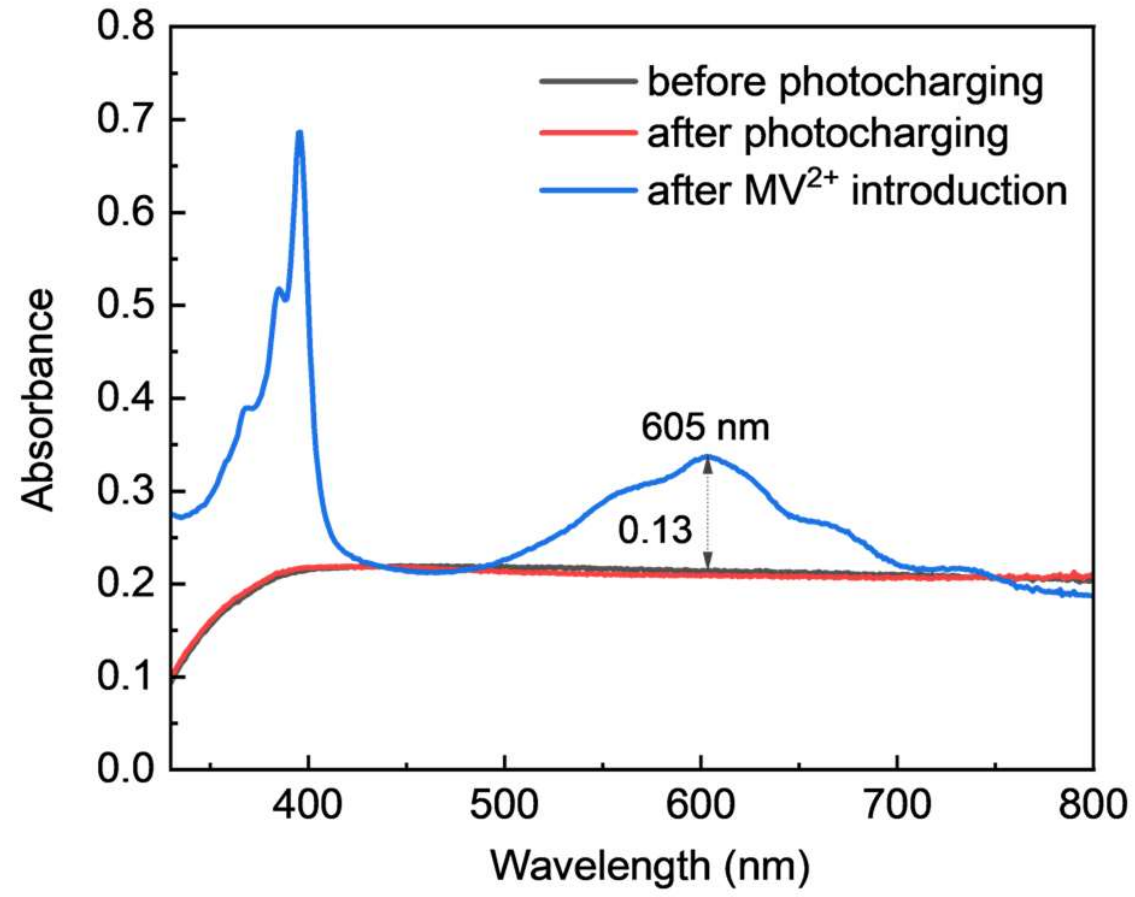

Figure S63. UV-vis spectra of CTF-Bpy before photocharging (100 μg CTF-Bpy in 2.75 mL 20 mM 4-MBA aqueous suspension), after photocharging (365 nm/10 min) and after the addition of Ar purged methyl viologen (MV^{2+}) (100 mM in water, 0.25 mL). Upon the addition of MV^{2+}, the suspension quickly changed to blue color, suggesting the reduction of MV^{2+} to the one-electron reduced methyl viologen radical cation, $MV^{\bullet+}$.

The absorbance difference value (ΔA) is marked by a black dashed line. From ΔA after MV^{2+} addition and the mass of CTF-Bpy, the accumulated electrons per gram of CTF were calculated to amount to 0.285 mmol g^{-1}, according to the Beer-Lambert law: $n(e^-) = \frac{\Delta A \cdot \boldsymbol{V}}{\mathcal{E} \cdot \boldsymbol{l}} \cdot \frac{1}{m}$, with V = 3.0 mL, Ɛ (the extinction coefficients) for $MV^{\bullet+}$ at 605 nm in water = 13 700 M^{-1} cm^{-1},[27] $\boldsymbol{l}$ (the light path) = 1 cm.

Table S5. Comparison of the number of accumulated electrons in CTF-Bpy determined by different spectroscopic methods and under different application conditions, together with the theoretical maximum electron-storage capacity.

**Quantification method**	**Number of e^- accumulated** (mmol g^{-1})	**Ratio to the theoretical maximum**	**Figure**
Theoretical maxium (130 mAh g^{-1})	4.85	-	Figure S39
Quantitative EPR	0.10	2.06 %	Figure S62
MV^{2+} reduction	0.285	5.88 %	Figure S63
Solar battery discharge (8.2 mAh g^{-1})	0.306	6.31 %	Figure 5a/5b
Dark H_2O_2 production (1100 µmol g^{-1})	2.2	45.36 %	Figure 6a
Dark H_2 evolution (68.1 µmol g^{-1} H_2 + 0.443 mmol g^{-1} for H_2PtCl_6 reduction to Pt^0)*	0.136 + 0.443 = 0.579	11.94 %	Figure 6b

*For the calculation of the number of accumulated electrons during the dark H_2 evolution experiment, we summed the number of electrons involved in the reduction of H_2PtCl_6 to Pt^0, as determined by ICP analysis of CTF-Bpy post dark H_2 evolution experiment, and the number of electrons involved in H_2 production.

9. Structure modelling

The structural model of CTFs was constructed using the zeo++ code[28, 29] based-on the 2D **hcb** topology. Geometry optimization of the generated model was performed with the Drieding force field, implemented in the Forcite module of the BIOVIA Materials Studio software. PXRD simulation of the CTF models was calculated in Mercury, a software from the Cambridge Structural Database (CSD). As the experimental PXRD of CTF-Bpy[6] and CTF-Bph[7] fits the reported patterns in AA stacking mode, we therefore only considered the AA-stacking here. Pawley refinement was performed for CTF-Bpy in the BIOVIA Materials Studio software.

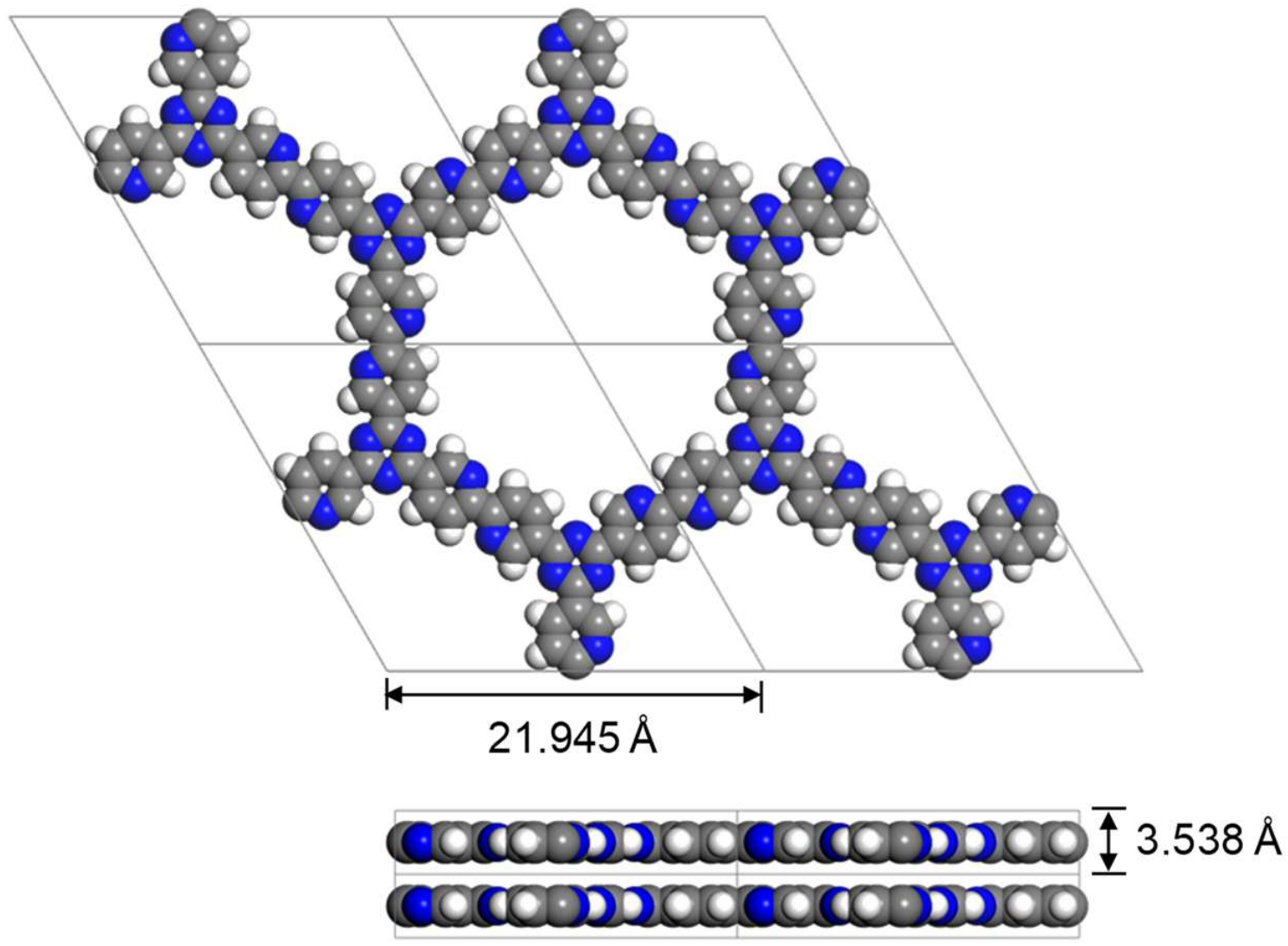

Figure S64. Crystal structure of CTF-Bpy in perfectly eclipsed AA stacking patterns. Gray, white and blue atoms represent carbon, hydrogen and nitrogen, respectively. Gray lines indicate the unit cell.

Table S6. Atomic coordinates and unit cell parameters for CTF-Bpy in AA-stacking mode.

CTF-Bpy, AA-stacking mode				
Space group: *P*6/*m* (No.175)<br>*a* = *b* = 21.9449 Å, *c* = 3.5378 Å				
Atom Name	x	y	z	Occupancy
C	0.48002	0.51717	0.5	1
C	0.4045	0.59142	0.5	1
C	0.3684	0.62955	0.5	1
N	0.51269	0.58869	0.5	1
C	0.47874	0.62593	0.5	1
H	0.50847	0.67949	0.5	1
C	0.59431	0.51979	0.5	1
C	0.63138	0.48298	0.5	1
H	0.6218	0.57352	0.5	1
H	0.68512	0.51123	0.5	1
N	0.40345	0.70076	0.5	1

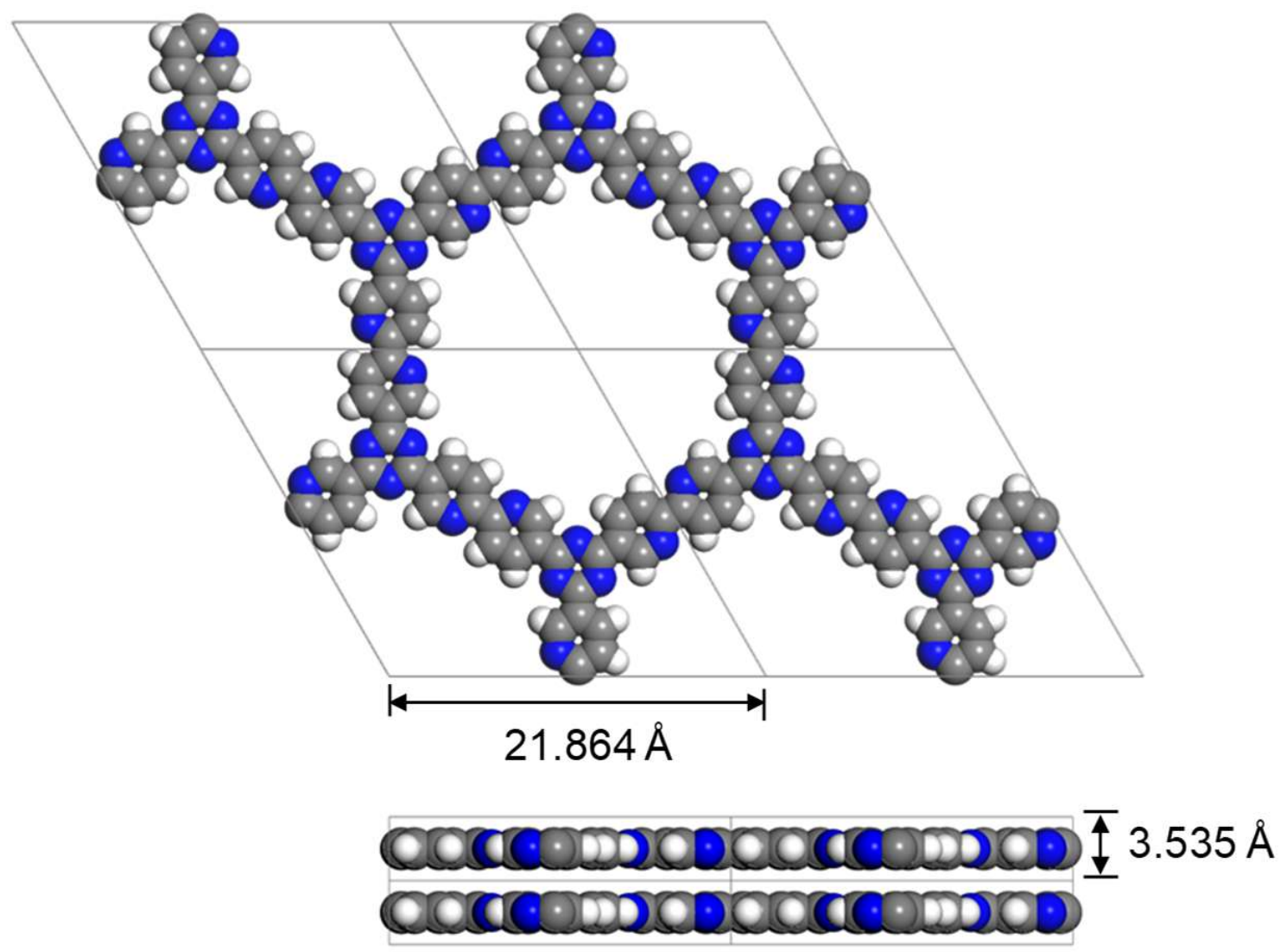

Figure S65. Crystal structure of the photocharged CTF-Bpy. Structural model was constructed based-on the one-electron reduction of each 2,2'-bipyridine (bpy) unit within CTF-Bpy. Gray, white and blue atoms represent carbon, hydrogen and nitrogen, respectively. Gray lines indicate the unit cell.

Table S7. Coordinates and unit cell parameters for the photocharged CTF-Bpy in AA-stacking mode. *Here the photocharged state corresponds to the one-electron reduced state of bpy.

photocharged CTF-Bpy, AA-stacking mode				
Space group: $P6/m$ (No.175)<br>a = b = 21.8636 Å, c = 3.5352 Å				
Atom Name	x	y	z	Occupancy
C	0.48312	0.51912	0.5	1
C	0.40888	0.59523	0.5	1
C	0.37057	0.63144	0.5	1
N	0.41127	0.48638	0.5	1
C	0.37416	0.52066	0.5	1
H	0.32038	0.49078	0.5	1
C	0.47963	0.40618	0.5	1
C	0.5164	0.36872	0.5	1
H	0.42548	0.37829	0.5	1
H	0.48803	0.31473	0.5	1
N	0.29908	0.5962	0.5	1

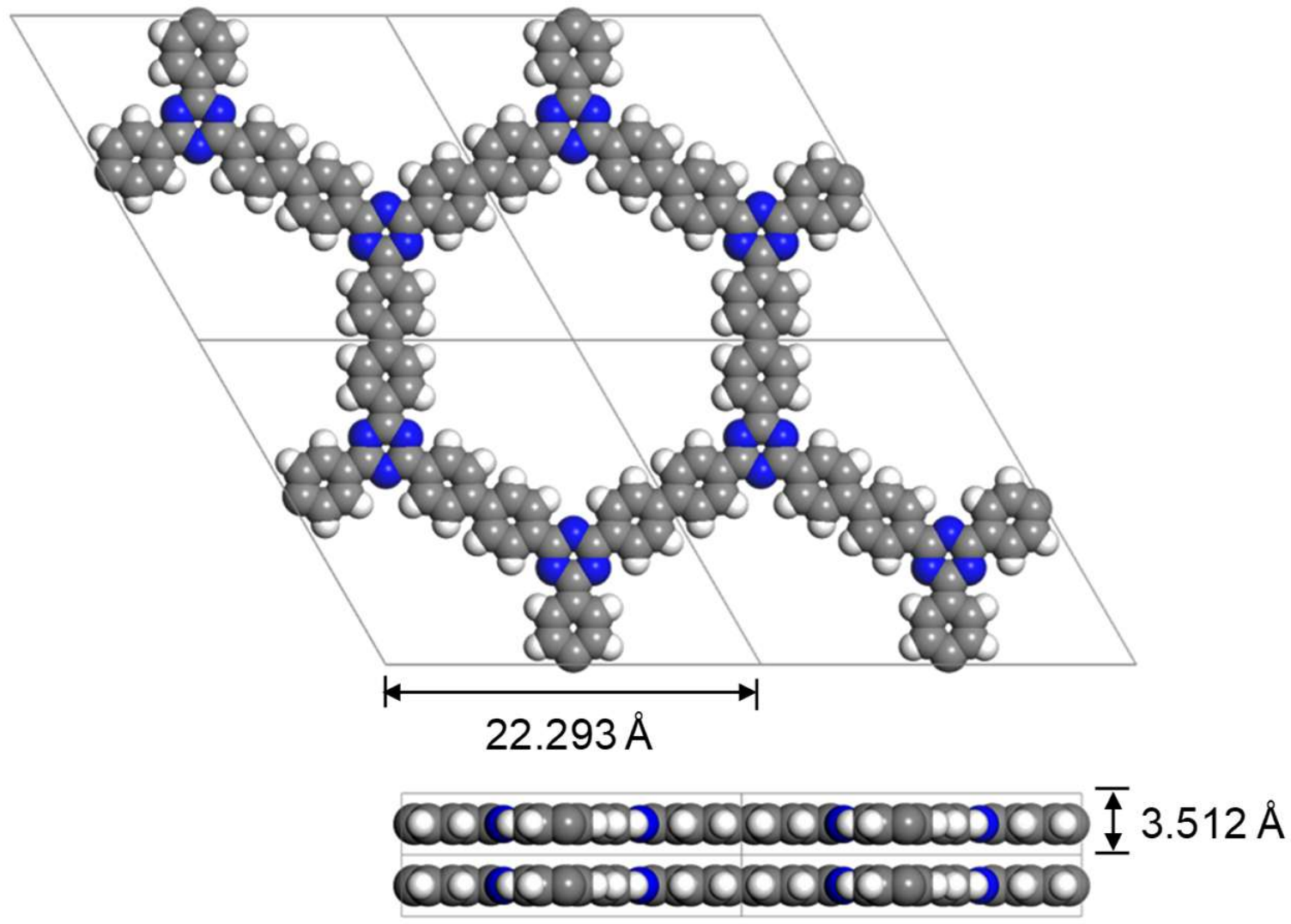

Figure S66. Crystal structure of CTF-Bph in perfectly eclipsed AA stacking patterns. Gray, white and blue atoms represent carbon, hydrogen and nitrogen, respectively. Gray lines indicate the unit cell.

Table S8. Atomic coordinates and unit cell parameters for CTF-Bph in AA-stacking mode.

CTF-Bph, AA-stacking mode				
Space group: *P6/mmm* (No.191)<br>*a* = *b* = 22.2934 Å, *c* = 3.5121 Å				
Atom Name	x	y	z	Occupancy
C	-0.51866	-0.48134	1.5	1
C	-0.59434	-0.40566	1.5	1
C	-0.63103	-0.36897	1.5	1
C	-0.48495	-0.40759	1.5	1
C	-0.52121	-0.37094	1.5	1
H	-0.43251	-0.37757	1.5	1
H	-0.49321	-0.31795	1.5	1
N	-0.59751	-0.29875	1.5	1

10. References

(1) Osterrieth, J. W.; Rampersad, J.; Madden, D.; Rampal, N.; Skoric, L.; Connolly, B.; Allendorf, M. D.; Stavila, V.; Snider, J. L.; Ameloot, R.; et al. How reproducible are surface areas calculated from the BET equation? *Adv. Mater.* **2022**, *34* (27), 2201502.
(2) Stoll, S.; Schweiger, A. EasySpin, a comprehensive software package for spectral simulation and analysis in EPR. *J. Magn. Reson.* **2006**, *178* (1), 42–55.
(3) Lau, V. W.-h.; Klose, D.; Kasap, H.; Podjaski, F.; Pignié, M.-C.; Reisner, E.; Jeschke, G.; Lotsch, B. V. Dark Photocatalysis: Storage of Solar Energy in Carbon Nitride for Time-Delayed Hydrogen Generation. *Angew. Chem. Int. Ed.* **2017**, *56* (2), 510–514.
(4) Wei, Z.; Liu, M.; Zhang, Z.; Yao, W.; Tan, H.; Zhu, Y. Efficient visible-light-driven selective oxygen reduction to hydrogen peroxide by oxygen-enriched graphitic carbon nitride polymers. *Energy Environ. Sci.* **2018**, *11* (9), 2581–2589.
(5) Rodríguez-Camargo, A.; Terban, M. W.; Paetsch, M.; Rico, E. A.; Graf, D.; Hirpara, R.; Duppel, V.; Moudrakovski, I.; Etter, M.; Guijarro, N.; et al. Cyclopalladation of a covalent organic framework for near-infrared-light-driven photocatalytic hydrogen peroxide production. *Nat. Synth.* **2025**, *4* (6), 710–719.
(6) Hu, X.; Zheng, L.; Wang, S.; Wang, X.; Tan, B. Integrating single Co sites into crystalline covalent triazine frameworks for photoreduction of CO2. *Chem. Commun.* **2022**, *58* (58), 8121–8124.
(7) Sun, R.; Hu, X.; Shu, C.; Guo, Y.; Wang, X.; Tan, B. Covalent triazine frameworks with Ru molecular catalyst for efficient photocatalytic oxygen evolution reaction. *Sci. China Mater.* **2024**, *67* (2), 642–649.
(8) Streater, D. H.; Kennehan, E. R.; Wang, D.; Fiankor, C.; Chen, L.; Yang, C.; Li, B.; Liu, D.; Ibrahim, F.; Hermans, I.; et al. Control over charge separation by imine structural isomerization in covalent organic frameworks with implications on CO2 photoreduction. *J. Am. Chem. Soc.* **2024**, *146* (7), 4489–4499.
(9) Scarborough, C. C.; Wieghardt, K. Electronic Structure of 2,2′-Bipyridine Organotransition-Metal Complexes. Establishing the Ligand Oxidation Level by Density Functional Theoretical Calculations. *Inorg. Chem.* **2011**, *50* (20), 9773–9793.
(10) Creutz, C. Bipyridine radical ions. *Comments Inorg. Chem.* **1982**, *1* (5), 293–311.
(11) Buntinx, G.; Poizat, O.; Valat, P.; Wintgens, V.; Righini, R.; Foggi, P. Transient absorption and resonance Raman studies of the photoreduction of 2, 2'-bipyridine by amines. *J. Chim. Phys.* **1993**, *90*, 1733–1748.
(12) Socrates, G. *Infrared and Raman characteristic group frequencies: tables and charts*; John Wiley & Sons, 2004.
(13) Parker, J. F.; Ko, J. S.; Rolison, D. R.; Long, J. W. Translating Materials-Level Performance into Device-Relevant Metrics for Zinc-Based Batteries. *Joule* **2018**, *2* (12), 2519–2527.
(14) Gao, H.; Neale, A. R.; Zhu, Q.; Bahri, M.; Wang, X.; Yang, H.; Xu, Y.; Clowes, R.; Browning, N. D.; Little, M. A.; et al. A pyrene-4, 5, 9, 10-tetraone-based covalent organic framework delivers high specific capacity as a Li-ion positive electrode. *J. Am. Chem. Soc.* **2022**, *144* (21), 9434–9442.
(15) Zhao, W.; Yan, P.; Li, B.; Bahri, M.; Liu, L.; Zhou, X.; Clowes, R.; Browning, N. D.; Wu, Y.; Ward, J. W.; et al. Accelerated synthesis and discovery of covalent organic framework photocatalysts for hydrogen peroxide production. *J. Am. Chem. Soc.* **2022**, *144* (22), 9902–9909.
(16) Wilke, T.; Schneider, M.; Kleinermanns, K. 1, 4-Hydroquinone is a hydrogen reservoir for fuel cells and recyclable via photocatalytic water splitting. *Open J. Phys. Chem.* **2013**, *3* (02), 97–102.
(17) Zeng, Z.; Quan, X.; Yu, H.; Chen, S.; Zhang, Y.; Zhao, H.; Zhang, S. Carbon nitride with electron storage property: Enhanced exciton dissociation for high-efficient photocatalysis. *Appl. Catal. B Environ.* **2018**, *236*, 99–106.
(18) Schlomberg, H.; Kröger, J.; Savasci, G. k.; Terban, M. W.; Bette, S.; Moudrakovski, I.; Duppel, V.; Podjaski, F.; Siegel, R.; Senker, J.; et al. Structural insights into poly (heptazine imides): a light-storing carbon nitride material for dark photocatalysis. *Chem. Mater.* **2019**, *31* (18), 7478–7486.
(19) Luo, L.; Wang, S.; Zhang, L.; Xiao, X.; Wu, B.; Jaroniec, M.; Jiang, B. Unveiling enhanced dark photocatalysis: Electron storage-enabled hydrogen production in polymeric carbon nitride. *Appl. Catal. B Environ.* **2024**, *343*, 123475.
(20) Amthor, S.; Knoll, S.; Heiland, M.; Zedler, L.; Li, C.; Nauroozi, D.; Tobaschus, W.; Mengele, A. K.; Anjass, M.; Schubert, U. S.; et al. A photosensitizer–polyoxometalate dyad that enables the decoupling of light and dark reactions for delayed on-demand solar hydrogen production. *Nat. Chem.* **2022**, *14* (3), 321–327.
(21) Pan, Y.; Wang, J.; Chen, S.; Yang, W.; Ding, C.; Waseem, A.; Jiang, H.-L. Linker engineering in metal–organic frameworks for dark photocatalysis. *Chem. Sci.* **2022**, *13* (22), 6696–6703.
(22) Stanley, P. M.; Sixt, F.; Warnan, J. Decoupled solar energy storage and dark photocatalysis in a 3D metal–organic framework. *Adv. Mater.* **2023**, *35* (1), 2207280.

(23) Wu, S.; Stanley, P. M.; Deger, S. N.; Hussain, M. Z.; Jentys, A.; Warnan, J. Photochargeable Mn-Based Metal–Organic Framework and Decoupled Photocatalysis. *Angew. Chem. Int. Ed.* **2024**, *63* (37), e202406385.
(24) Yao, S.; Heinzerling, K.; Hillman, S. A.; Podjaski, F.; He, T.; García-Baldoví, A.; Baghdadi, Y.; Dassouki, K.; García, H.; Eslava, S.; et al. Photoaccumulation of Long-Lived Reactive Electrons in a Microporous Ti (IV) Oxocluster-Based Metal–Organic Framework for Light and Dark Photocatalysis. *Adv. Mater.* **2026**, *38* (8), e17595.
(25) Knapp, J. E.; Rath, B. B.; Ortín-Rubio, B.; Bauder, L.; Gjorgjevikj, K.; Das, S.; Tesi, L.; Baumgartner, B.; Krause, S.; Lotsch, B. V. Charge Storage and Dark Photocatalysis in Multivariate Ti-Based Metal-Organic Frameworks. *ChemRxiv* **2026**, *2026* (0520).
(26) Wang, Y.; Chan, Y.-T.; Oshima, T.; Duppel, V.; Bette, S.; Küster, K.; Gouder, A.; Scheurer, C.; Lotsch, B. V. Decoupling of light and dark reactions in a 2D niobium tungstate for light-induced charge storage and on-demand hydrogen evolution. *J. Am. Chem. Soc.* **2024**, *146* (37), 25467–25476.
(27) Hare, P. M.; Price, E. A.; Bartels, D. M. Hydrated electron extinction coefficient revisited. *J. Phys. Chem. A.* **2008**, *112* (30), 6800–6802.
(28) Willems, T. F.; Rycroft, C. H.; Kazi, M.; Meza, J. C.; Haranczyk, M. Algorithms and tools for high-throughput geometry-based analysis of crystalline porous materials. *Microporous Mesoporous Mater.* **2012**, *149* (1), 134–141.
(29) Martin, R. L.; Haranczyk, M. Construction and Characterization of Structure Models of Crystalline Porous Polymers. *Cryst. Growth Des.* **2014**, *14* (5), 2431–2440.
